# A systematic review and meta-analysis of comprehensive interventions for pre-school children with autism spectrum disorder (ASD)

**DOI:** 10.1371/journal.pone.0186502

**Published:** 2017-12-06

**Authors:** Yoshiyuki Tachibana, Celine Miyazaki, Erika Ota, Rintaro Mori, Yeonhee Hwang, Eriko Kobayashi, Akiko Terasaka, Julian Tang, Yoko Kamio

**Affiliations:** 1 Division of Infant and Toddler Mental Health, Department of Psychosocial Medicine, National Centre for Child Health and Development, Tokyo, Japan; 2 Smart Aging International Research Center, IDAC, Tohoku University, Sendai, Japan; 3 Department of Child and Adolescent Mental Health, National Center for Neurology and Psychiatry, Tokyo, Japan; 4 Department of Health Policy, National Research Institute for Child Health and Development, Tokyo, Japan; 5 Department of Education, Tohoku Fukushi University, Sendai, Japan; 6 Department of Educational Collaboration, Osaka Kyoiku University, Osaka, Japan; 7 Department of Education for Clinical Research, National Centre for Child Health and Development, Tokyo, Japan; TNO, NETHERLANDS

## Abstract

**Background:**

There has an increasing number of published trials on psychosocial intervention programmes for pre-school children with autism spectrum disorder (ASD). To achieve better quality of unbiased evidence for the effectiveness of ASD interventions, it is necessary to conduct a comprehensive review that covers studies with adequate quality standards, such as randomised controlled trials (RCTs), and different types of intervention In this study, we categorize interventions for ASD as behavioural, social-communication focused, and multimodal developmental based on Howlin’s classification of early interventions for children with ASD. The aim of this study was to compare these three models and investigate the strengths and weaknesses of each type of intervention and to identify the approaches that contribute to a successful outcome for children with autism.

**Methods:**

We performed a systematic review and meta-analysis. We included RCTs targeting children with ASD 6 years old or younger. A random effects model was used to present the effect estimate for the outcomes. This study also performed combined meta-analyses of all the three models to investigate the overall effectiveness of the intervention programmes.

**Results:**

32 randomized controlled studies were found to be eligible for inclusion. The synthesized data included 594 children from 14 RCTs. There was no statistically significant difference in the effects on autism general symptoms between the social-communication-focused model and the multimodal developmental model (p = 0.83). The results suggest that there is evidence of an effect on ‘reciprocity of social interaction towards others’ (standard mean difference [95% confidential interval] = 0.53[0.29,0.78], p<0.01) and ‘parental synchrony’ (SMD = 0.99[0.70,1.29], p<0.01).

**Conclusion:**

The small number of studies included in the present study limited the ability to make inferences when comparing the three models and investigating the strengths and weaknesses of each type of intervention with respect to important outcomes. Since the outcome of ‘reciprocity of social interaction towards others’ could be a dependent variable that might be context-bound to interactions with the child’s parent, we cannot conclude the interventions for pre-school children with ASD have significant effects on a generalized skill to engage in reciprocal interactions with others. However, the outcomes of ‘reciprocity of social interaction towards others’ and ‘parental synchrony’ may be promising targets for interventions involving pre-school children with ASD.

**Trial registration:**

Prospero CRD42011001349

## Introduction

Autism spectrum disorder (ASD) is a persistent disabling neurodevelopmental disorder [1] that begins to present symptoms early in life. In recent years, many countries have given greater public attention to ASD and allocated more public funds to promoting research and implementing community services for ASD patients [2–5]^,^. Early intervention is a key issue for public health policy for ASD [6, 7]. There is growing evidence that early intervention programmes improve the developmental functioning, maladaptive behaviour, and symptom severity of children with ASD [8–10]. In addition, early interventions might also affect outcomes in later years for many individuals [11].

There has been an increasing number of published trials on psychosocial intervention programmes for pre-school children with ASD. A growing number of these studies has followed the Consolidated Standards of Reporting Trials (CONSORT) guidelines [12], and some meta-analyses and systematic reviews of intervention programmes for pre-school children with ASD have been published; e.g. [13–15]. However, the reported intervention effects have not been comparable due to the different approaches taken by the interventions, variety of outcomes measured and specific terms used for autism symptoms. In the case of autism symptom outcomes, these could be categorized into non-autism-specific outcomes (e.g. IQ) or intermediate outcomes related to aspects of development that link to later autism symptoms (e.g. changes in joint attention or parent–child interaction). The heterogeneous investigation into the effectiveness of different types of interventions for specific autism symptoms has hindered the ability of clinicians and policymakers to make necessary decisions. To achieve better quality of unbiased evidence for the effectiveness of ASD interventions, it is necessary to conduct a comprehensive review that covers studies with adequate quality standards, such as randomised controlled trials (RCTs), and different types of interventions [16]. Howlin *et al*. asserted three main strands of early interventions for children with ASD)[17]: i) programmes with a particular emphasis on the use of behavioural principles to improve learning and behaviour (e.g. [18]); ii) those that have a specific focus on communication (e.g. [19]); and iii) those in which developmental/educational strategies have been employed (e.g. [20]). In this study, we named these strands as behavioural, social-communication focused, and multimodal developmental interventions, respectively [21]. Comparing the three models and investigating each type of intervention’s strengths and weaknesses with respect to important outcomes will help to identify the approaches that support successful intervention effects. Such an analysis will also reveal how evidence-based assessments of effective methods can be introduced as treatments for children with ASD. The findings will contribute to determining the appropriate choice of intervention for children with ASD for their families, clinicians, and policymakers.

The objective of our study was to conduct a systematic review and meta-analysis of methodologically adequate studies in accordance with the Cochrane Handbook [22], which allowed, for the first time, a comparison of different approaches to interventions on different outcome measures.

## Methods

The methods used to conduct this study were in accordance with the Cochrane Handbook for Systematic Reviews [23]. The PRISMA guideline [24] was used to prepare this review [21] (See Supporting Information 1 and 2).

### Selection criteria

#### Types of studies

We included randomised controlled trials (RCTs). We excluded quasi-RCTs and crossover trials.

#### Types of participants

Participants were children aged six years old or younger, with a diagnosis of ASD as below.

Diagnostic and Statistical Manual of Mental Disorders Third Edition—Revised (DSM-III-R) [25]

Diagnostic and Statistical Manual of Mental Disorders Fourth Edition (DSM-IV) [26]

Diagnostic and Statistical Manual of Mental Disorders-Ⅳ-Text Revision (DSM-Ⅳ-TR) [27]

- Autistic disorder
- Asperger disorder
- Pervasive developmental disorder not otherwise specified (PDD-NOS)

International Classification of Diseases-10 (ICD-10) [28]

- Childhood autism
- Asperger syndrome, atypical autism
- Other pervasive developmental disorders
- Pervasive developmental disorders, unspecified.

Diagnostic and Statistical Manual of Mental Disorders-5 (DSM-5)[29].

- Autism spectrum disorder

#### Types of interventions

Three types of interventions were targeted in this study; i) behavioural interventions–based essentially on learning theory and on applied behaviour analysis (ABA) (not limited to only early intensive behavioural intervention [30, 31], but also included ABA programmes derived from it; ii) social-communication focused interventions, targeting social communication impairment, as the core symptom of autism; iii) multimodal developmental interventions targeting a comprehensive range of children’s development. Studies with interventions delivered to the parents/guardians and/or directly to the child, by special educators, teachers, speech pathologists, psychologists, or other allied health professional students were included. We classified those three types of interventions as behavioural model, social-communication focused model, and multimodal developmental model, respectively. We limited the analysis to pre-school children aged six years old or younger.

The studies whose interventions were not classified into behavioural, social-communication focused or multimodal developmental were not included. The studies whose interventions were pharmacological, alternative or complementary medicine were not included as well. The studies in which the control group received a specific early intervention for children with ASD, which was not considered treatment as usual provided by their local services, were excluded.

#### Types of outcomes

A feature of this review is that we systematically classified the various outcome measures used within recent intervention trials into the following categories. Each type of intervention measured different outcomes. For example, behavioural model interventions tended to focus on IQ, adaptive behaviour and language development. Social-communication focused model interventions tended to address language, social interaction, and autism symptoms. Multimodal developmental model interventions tended to measure developmental quotient, language, social interactions, and autism symptoms. Among many outcomes, we prioritized autism symptoms (as primary outcomes) (I). In addition, we also investigated other relevant outcomes as below (II) which are important for children’s daily life and prognosis. We chose the outcomes that had been investigated in previous studies [32–38].

**I**. **Primary outcomes**

1.1. Autism general symptoms

This outcome indicated the severity of autism symptoms related to the definitional symptoms of autistic disorder in DSM-IV-TR (e.g. the comparison score of the autism Diagnostic Observation Schedule-Generic [39] was used for these outcomes.).

**II**. **Secondary outcomes**

Non-specific developmental outcomes, which are not required for definition to autism diagnosis but are used in some studies were used for the syntheses. Here, we defined “Developmental quotient” integrating developmental quotient and intelligence quotient. In this paper, we report five outcomes as below. “Reciprocity of social interaction towards others” was defined as a child’s reciprocal social interactions with his or her parents or examiners.

2.1. Developmental quotient

2.2. Expressive language

2.3. Receptive language

2.4. Reciprocity of social interaction towards others

2.5. Adaptive behaviour

We also report other outcomes as follows in the appendices. Parental synchrony (3.9) was defined as parental synchronous response to his or her child.

See Appendices regarding the outcomes as below.

3.1. Qualitative impairment in social interaction

3.2. Qualitative impairment in communication

3.3. Restricted repetitive and stereotyped patterns of behaviour, interests, and activities (RRB)

3.4. Initiating joint attention

3.5. Responding to joint attention

3.6. Imitation

3.7. Symbolic play

3.8. Functional play

3.9. Parental synchrony

3.10 Parenting stress

Regarding autism symptom outcomes 1.1, 3.1, 3.2, and 3.3, studies without objective coding with child observation (e.g. only using parent questionnaire or parent interview) were excluded in the analyses.

### Electronic searches (See S1 Appendix)

We searched the following databases: PsycINFO, Medline via Ovid, ERIC, CINHAL, and the Cochrane Central Register of Controlled Trials (CENTRAL) without any language restriction in 1^st^ October 2014.

We used the following search terms to search all trials registers and databases: “autism”, “autism spectrum disorder”, “ASD”, “high function autism”, “high function ASD”, “Asperger syndrome”, “pervasive developmental disorder”, “PDDNOS”, “intervention”, “treatment”, “therapy”, “communication”, “interpersonal”, “speech”, “interaction”, “synchrony”, “relationship”, “language”, “social”, “development”, “behavior”, “intensive behavioral intervention”, “trial”, and “outcome”. Their search were limited by age group from zero to six years old and “randomised controlled trial.” This search strategy has been peer-reviewed by librarians of University of Manchester and the National Research Centre for Child Health and Development. Other relevant studies were also searched from the reference lists to identified trials and review articles. The ClinicalTrials.gov and the Cochrane Library website were also searched for randomised trials that were registered as completed but not yet published.

### Searching other resources

Reference lists from identified trials and review articles were manually scanned to identify any other relevant studies. The ClinicalTrials.gov and the Cochrane Central Register of Controlled Trials (CENTRAL) were also searched for randomised trials that were registered as completed but not yet published.

### Data collection and analysis

All references found by the search strategy were gathered by the reference management programme EndNote X6. Two of the authors, A.T. and E.K. independently reviewed the abstracts of the potentially relevant studies. All citations sourced from the search strategy were transferred to EndNoteX6 (Thomson Reuters, New York City, USA), a reference management database software. Initial screening of titles and abstracts by E.K. eliminated all those citations obviously irrelevant to the topic, for example, prevalence studies, studies not relating to autism spectrum disorders, and single case studies. Thereafter, two out of five review authors (YT, YH, EK, CM, and AT) assessed and select studies for inclusion from the group of potentially relevant studies. In the event of a disagreement, resolutions were reached in discussion with the third referees (EK or YT), if necessary following inspection of the full paper.

### Data extraction and management

EK and CM independently extracted data from selected trials using a specially designed data extraction form. Extracted data consisted of methods (dose and frequency of intervention); diagnostic description of participants, and type of intervention, including target, intensity, duration and method of application (parent-mediated, therapist, school-based etc.). Data were extracted independently by two review authors (EK and CM) and disagreements were resolved by negotiation with a third author (YT).

### Assessment of risk of bias in included studies

Risks of bias were assessed by five independent review authors (YT, YH, EK, AT, and CM) and disagreements were resolved by negotiation with a third review author (YT or EK). We used the Cochrane Collaboration’s tool for assessing risk of bias for each included studies [40]. The tool included the following domains: sequence generation; allocation concealment; blinding of participants and personnel; blinding of outcome assessment; complete outcome data; selective outcome reporting; other sources of bias. The process involved recording the appropriate information for each study (for example describing the method used to conceal allocation in detail) and evaluating whether there is risk of bias in that area (for example, was allocation adequately concealed). We allocated studies to the three categories according to our judgment of each area or potential risk of bias: “low risk of bias”, “unclear risk of bias”, and “high risk of bias”. Whether the studies should be included for the meta-analyses were judged individually based on the results of the risk of bias assessments and studies judged to be at high risk of bias were excluded. Final inclusion of articles for the meta-analyses was judged by YT and EK, and supervised by OE based on the results of the risk of bias assessments.

### Dealing with missing data

For each study, missing data were handled by considering when there is a significant loss of quantities of participant data in the report, such that the review authors agree the conclusions of the study were compromised, the trial authors were contacted. If no reply was forthcoming or full data were not made available, these studies were excluded from the final analysis. We reported the number of participants in the final analysis from the proportion of those participants who began the intervention as reporting drop-out rates in the included studies. We also reported reasons for missing data provided by the included studies. We assessed the extent to which the results of the review could be altered by the missing data using sensitivity analyses, which included the studies that had been excluded due to “incomplete outcome data,” as well as other exclusion criteria.

### Assessment of reporting biases

When there is a sufficient number of studies (10 or more), funnel plots were drawn to detect the distribution of the studies by their effect and sample sizes. Such a relationship could be the result of publication or related biases, or due to systematic differences between small and large studies. Every attempt was made to obtain unpublished data and data from conference proceedings.

### Data synthesis

Data syntheses were performed using Review Manager version 5.3 (Cochrane Collaboration software). We assessed continuous data. Continuous data were analysed on the basis that the means and standard deviations were available and that there was no clear evidence of skew in the distribution. Assuming that two or more studies that were suitable for inclusion were found, and that the studies were considered to be satisfactory, a meta-analysis was performed on the results. To compare the three types of interventions, we categorised the studies into subgroups based on the three intervention models described earlier. Since the studies measured several outcomes in a nonuniform manner, outcome data was synthesized using standardised mean difference (mean/standard deviation post-intervention) for both intervention and control groups. The syntheses were based on previous meta-analysis studies of interventions for children with ASD [41, 42]. We synthesized the various categories of outcome measures using a standardised mean difference (mean/standard deviation at the post-intervention) for both groups. We tested the three subgroup interactions (i.e. behaviour interventions versus social-communication focused interventions versus multimodal developmental interventions) by an inverse variance method in a random effects model [40]. We used the I^2^ statistic to assess the rationale of data synthesis based on the degree to which there was heterogeneity in the types of measurement in the included studies [40]. We also performed an overall synthesis of the included studies for the three models on each outcome. We analysed these studies in the same way in which the previous meta-analysis amalgamated the studies of children with ASD, which had different modalities of interventions and different measurements [41, 42].

### Main analysis

#### Analysis I (analysis excluding studies at a high risk of bias using a random effects model)

We performed data syntheses excluding the studies that were assessed to have a ‘high’ or ‘unclear’ risk of bias in both ‘Random sequence generation (selection bias)’ or ‘Allocation concealment (selection bias)', and studies with a ‘high’ risk of bias in ‘Incomplete outcome data (attrition bias)’).

We also performed sensitivity analyses as below. To interpret the present study’s results, we prioritized Analysis I. If there was a discrepancy in the results of Analysis I and the sensitivity analyses, we carefully assessed the reasons for these discrepancies in our interpretation.

### Sensitivity analysis

#### Analysis II (analysis of all the included studies using a random effects model)

We undertook a sensitivity analysis with all the included studies to explore the extent to which studies excluded in Analysis I could affect the results.

#### Analysis III (analysis excluding studies at a high risk of bias using a fixed effects model) and Analysis IV (analysis of all the included studies using a fixed effects model)

We employed a random effects model for Analysis I and II. As part of our sensitivity analysis, we also, however, examined all the results with a fixed effects model to see if they changed, and reported any discrepancies as Analysis III and IV for Analysis I and II, respectively.

If a study included in a meta-analysis had a significant baseline imbalance, we also performed a sensitivity analysis removing the study.

### ‘Summary of findings for the main outcomes’ table (Table 1)

We created a ‘Summary of findings for the main outcomes’ table (Table 1), which shows the review’s main outcomes that may be important to parents, clinicians, and decision makers. We used the Grades of Recommendation, Assessment, Development and Evaluation (GRADE) system [43] to describe the quality of the evidence and the strength of recommendation, and used GRADEpro software [44] to construct the tables. We expressed the quality of evidence on a four-point adjectival scale; ‘high’, ‘moderate’, ‘low’, ‘very low’.

**Table 1:** Summary of findings for the main outcomes (Analysis I: random effects model, 14 studies). Comprehensive interventions for pre-school children with autism spectrum disorder (ASD).**Population:** Pre-school children aged 0 to 6 with a diagnosis of ASD. **Settings:** Australia, Canada, Japan, Norway, UK, and USA. **Intervention:** Behavioural interventions, social-communication focused interventions, multimodal developmental interventions.

Outcomes	Illustrative comparative risks* (95% CI)		Relative effect	No of Participants	Quality of evidence	Comments
			(95% CI)	(studies)	(GRADE)	
	Assumed risk	Corresponding risk				
	Control	Autism general symptoms				
**Autism general symptoms**	The mean ‘autism general symptoms’ was 0	The mean ‘autism general symptoms’ in the intervention groups was	SMD	227	⊕⊕⊕⊝	
		**0.31 standard deviations lower**	-0.31 (-0.63. to 0.01)	(3 studies)	**moderate**^2^	
		(-0.63 to 0.01 higher)				
**Developmental quotient**	The mean ‘developmental quotient’ was **0**	The mean ‘developmental quotient’ in the intervention groups was	SMD	208*	⊕⊕⊕⊝	These are the results of the sensitivity analysis excluding studies with a significant baseline imbalance.
		**0.31 standard deviations higher**	0.31 (-0.02 to 0.65)*	(4 studies)	**moderate**^2^	
		(-0.02 to 0.65 higher)				
**Expressive language**	The mean ‘expressive language’ was 0	The mean ‘expressive language’ in the intervention groups was	SMD	457	⊕⊕⊕⊝	
		**0.11 standard deviations higher**	0.11 (-0.07 to 0.3)	(8 studies)	**moderate**^1^	
		(-0.07 to 0.3 higher)				
**Receptive language**	The mean ‘receptive language’ was 0	The mean ‘receptive language’ in the intervention groups was	SMD	457	⊕⊕⊕⊝	
		**0.12 standard deviations higher**	0.12(-0.11 to 0.34)	(8 studies)	**moderate**^1^	
		(-0.11 to 0.34 higher)				
**Reciprocity of social interaction towards others**	The mean ‘reciprocity of social interaction towards others’ was **0**	The mean ‘reciprocity of social interaction towards others’ in the intervention group was **0.53 standard deviations higher** (0.29 more to 0.78 more)	SMD	380	⊕⊕⊕⊕	
			0.53 (0.29 to 0.78)	(8 studies)	**High**	
**Adaptive behaviour**	The mean ‘adaptive behaviour’ was **0**	The mean ‘adaptive behaviour’ in the intervention group was **-0.04 standard deviations higher** (-0.23 more to 0.15 more)	SMD	414	⊕⊕⊕⊝	
			-0.04 (-0.23 to 0.15)	(7 studies)	**moderate**^1^	

CI: confidence interval, SMD: standard mean difference. GRADE Working Group grades of evidence High quality: Further research is very unlikely to change our confidence in the estimate of effect. Moderate quality: Further research is likely to have an important impact on our confidence in the estimate of effect and may change the estimate. Low quality: Further research is very likely to have an important impact on our confidence in the estimate of effect and is likely to change the estimate. Very low quality: We are very uncertain about the estimate.

*The basis for the assumed risk (e.g. the median control group risk across studies) is provided in footnotes. The corresponding risk (and its 95% confidence interval) is based on the assumed risk in the comparison group and the relative effect of the intervention (and its 95% CI).

^1^ Small sample size with wide confidence interval crossing the line of no effect.

^2^ Estimate based on small sample size.

## Results

### Search results

**Fig 1:**
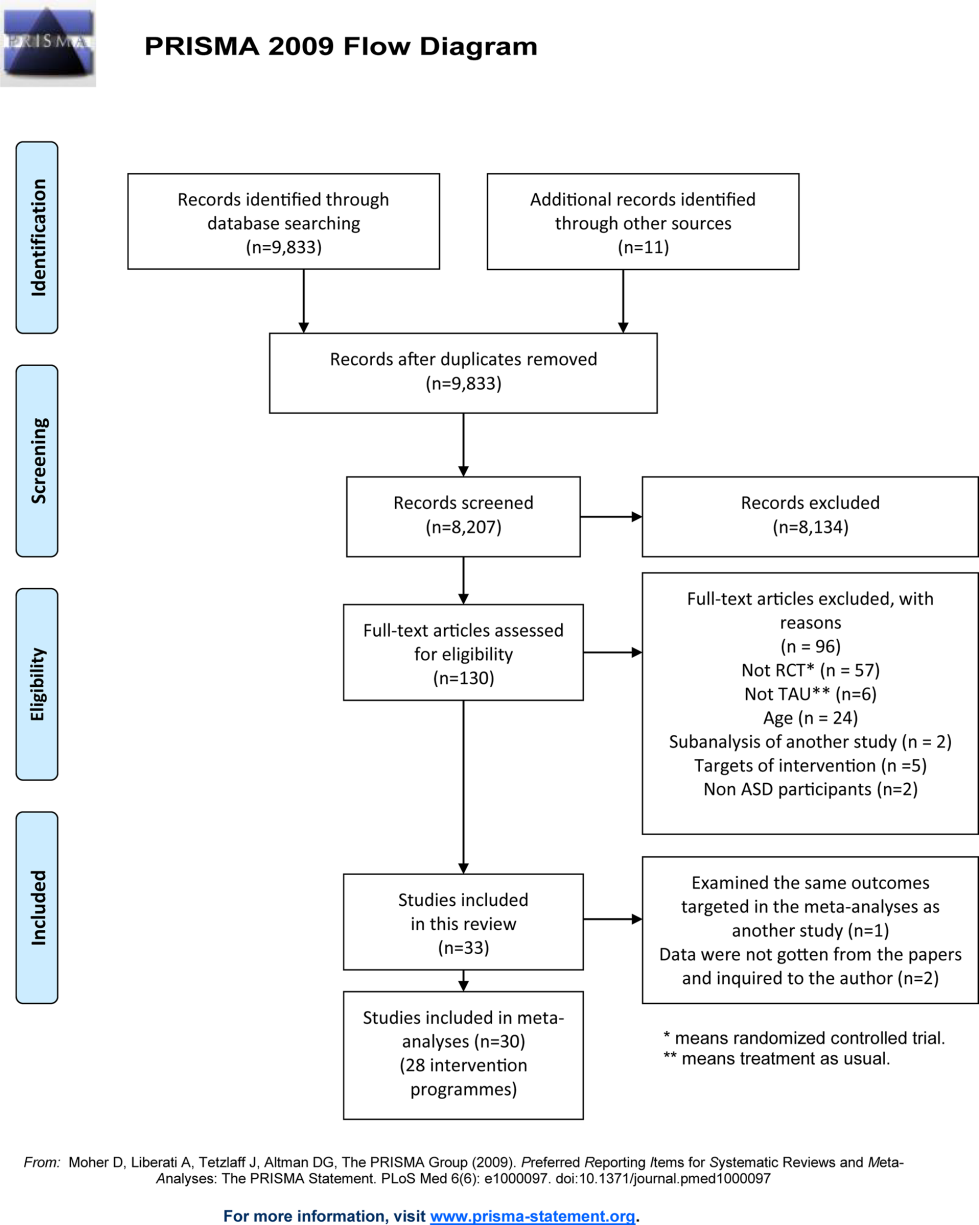
PRISMA 2009 flow diagram of this study.

Flow diagram of the search results is shown in Fig 1. We found 9833 citations using the search strategy run in October 2014 of which 8207 were abstracts of potential interests. In cases where clarification or further data were needed, the trial authors were contacted. We contacted authors of 10 trials, and four replied with the relevant data. Examination of the abstracts, full texts of reports and hand searching of articles and reference lists resulted in 96 excluded studies for the following reasons (see S1 Table). 57 studies were not RCTs. Six studies did not have “treatment as usual” control conditions. 24 studies did not match the inclusion criteria of age. Two studies were sub-analysis of their previous studies. Five studies’ did not fit the three models which are the target of this review. Two studies included children with non-ASD disabilities. 33 studies [18–20, 45–74] were included in this review. Four studies [54] among them reported other analysis results from the original studies ([54] from [55], [58] from [57], [70] from [69], [67] from [66], respectively), and one of them [58] examined the same outcomes targeted in this review’s meta-analyses as the original paper [57]. Two studies [73, 74] were excluded for the meta-analyses because required data for the analyses were not supplied from their papers and inquiries to the author. One study [63] investigated the effectiveness of two programmes compared to a comparison group. Thus, 30 papers [18–20, 45–57, 59, 61–72, 75] (28 intervention programmes [18–20, 45–53, 55–57, 59, 61–66, 68, 69, 71, 72, 75]) were included in the meta-analyses.

### Description of the included studies

See: Characteristics of included studies (S2 Table); Excluded studies (S1 Table)

All studies were RCTs and the active controls or the psychological placebo groups consisted of treatment as usual in local services for children with ASD.

### Intervention content

There were two included studies categorised into behavioural model [18, 62]. Eighteen studies were categorised into social-communication focused models [19, 45, 46, 49, 51, 52, 54–59, 65–68, 71, 75], which focused on communication, joint attention, and reciprocity of social interaction towards others. Nine studies were categorised into multimodal developmental models [20, 47, 50, 53, 61, 63, 64, 70, 72].

### Control condition

Our meta-analyses limited the targets only for the studies whose control condition were treatment as usual.

### Risk of bias

**Fig 2:**
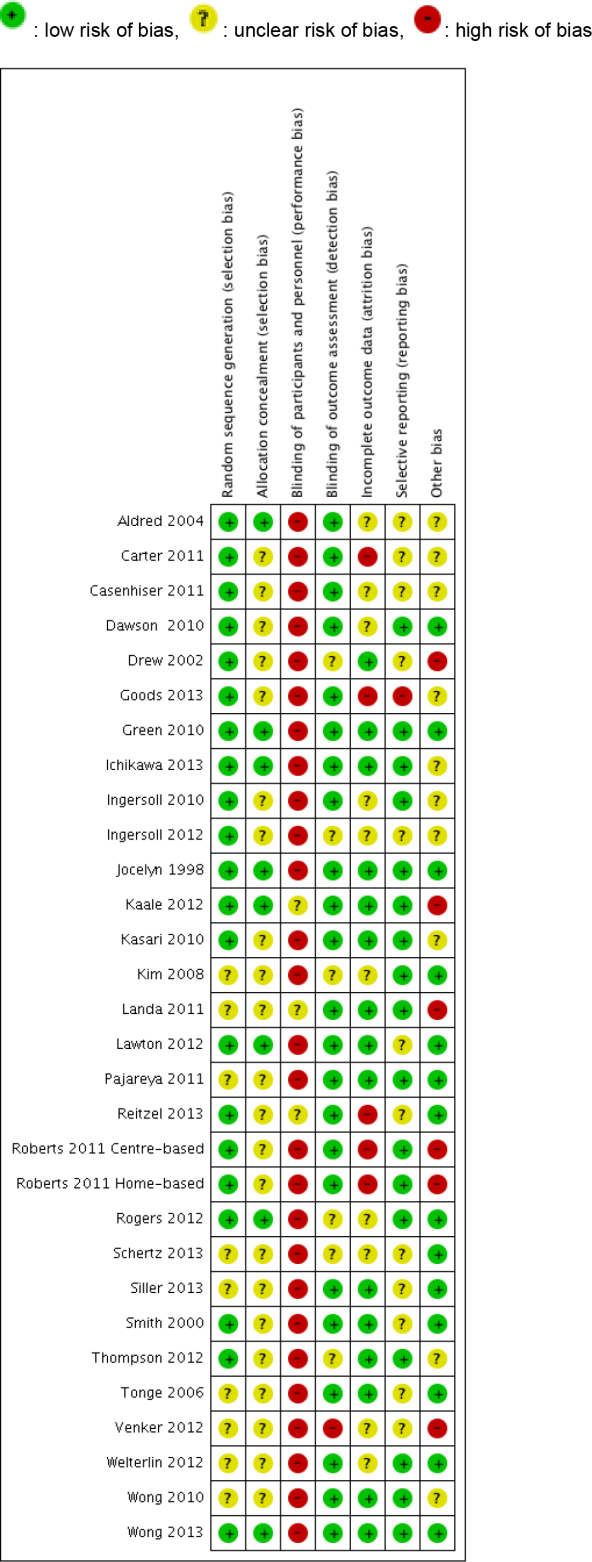
'Risk of bias' summary.

**Fig 3:**
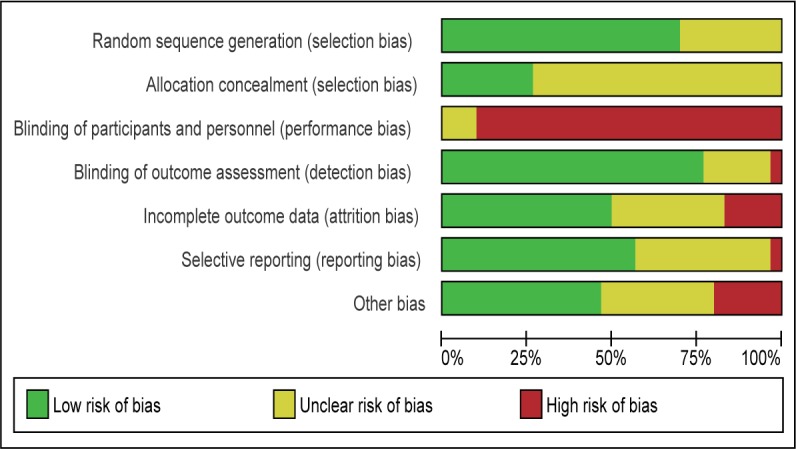
'Risk of bias' graph.

The summaries of “Risk of bias” judgements are shown in Fig 2 and Fig 3.

#### Summary

In summary, no study was found to have low risk of bias in all domains.

#### Random sequence generation

No trials were at high risk of bias for sequence generation (not truly random), however, the method of randomisation was unclear (not reported) in two trials. One study did not mention the detail of random sequence generation [76]. Another study [63] had three arms, and the two intervention arms were randomised with computer, but wait-list control group were not allocated with random sequence generation.

#### Allocation (selection bias)

Nine studies [19, 45, 50, 53–55, 64, 73, 75] mentioned allocation concealment, and the rest did not. Those which did not mention allocation concealment were rated as “unclear risk” of bias.

#### Blinding (performance bias)

No studies were rated as ‘low risk’ (four studies [54, 55, 59, 62] were rated as ‘unclear risk’ and the other twenty six studies [12–14, 42, 43, 45–49, 51–60, 63, 65–69, 71] were rated as ‘high risk’). This was because, for the interventions in this area, it is by definition impossible to blind parents and interveners from the intervention being performed.

#### Blinding (detection bias)

Detection bias was not found to be a major influence in most of the studies. One study was rated as “high risk” [71]. In the study, given staffing constraints, observational coding and reliability coding were conducted by two of the graduate student clinicians; thus it was not possible to maintain blindness to treatment group assignment.

#### Incomplete outcome data (attrition bias)

26 of the included studies addressed attrition in ways judged to be at ‘low risk’ or ‘unclear risk’ of bias. Four studies were rated as at high risk of bias because of high attrition rate (more than 20%) [49, 62, 63] and missing data [46].

#### Selective reporting (reporting bias)

One study were rated as at high risk of bias because discussion of a measure was left out of discussion [49].

### Effects of intervention

#### Quantitative data synthesis

We conducted meta-analyses for the 16 outcomes. Here, we regarded the studies with “high risk of bias” which were excluded in the quantitative data syntheses as below:

1. Studies with “unclear bias” in both of random sequence generation and allocation concealment
2. Studies with “high risk of bias” in incomplete outcome data

14 studies [18–20, 45–56, 60, 62, 64, 68, 75] (13 programmes) were included in Analysis I. The forest plots of Analysis I are shown in S1 Fig. As for the sensitivity analyses—analysis II, III, and IV—although Tonge *et al*. 2006 [69] and Tonge *et al*. 2014 [70] had two intervention conditions (i.e. parent education and behaviour management (PEBM) and parent education and counselling (PEC)), PEC condition was aimed at controlling for nonspecific therapeutic effects compared to the target intervention, PEBM. Thus, we did not use the PEC data, and included PEBM data for the data-syntheses.

#### Participants

There were a total of 594 children across the included studies. The smallest study in the review had 11 participants [50], while the largest [19] had 152 participants. Children were aged between one and six.

This systematic review and meta-analysis targeted children with ASD that complied with the international typical diagnostic standards. All child participants in the studies had a diagnosis of autism or ASD made by an assessing clinician or psychologist based on DSM-Ⅳ-TR, DSM-Ⅳ, DSM Ⅲ-R, or ICD-10 classification. No study was included in which children with ASD were diagnosed by DSM-5. Several of the studies used Autism Diagnostic Interview-Revised (ADI-R) or Autism Diagnostic Observation Schedule (ADOS), or both, to confirm diagnosis. None of the children had any comorbid or debilitating illness such as cerebral palsy, genetic syndromes, diagnosed hearing impairment, diagnosed visual impairment or seizures, or severe psychiatric disorders. Children’ ethnicities were white, Latino, Caribbean, Hispanic, African, Asian, and other mixed races. The nationalities of the children were UK, USA, Canada, Australia, Thailand, Korea, and Japan.

### Sensitivity analysis

#### Analysis II and IV

The results of Analysis II (random effects model) and IV (fixed effects model) involved 30 included studies [18–20, 45–57, 59, 61, 62, 64–72, 75]. The analyses involved 1220 children. The forest plots of Analysis II and IV are presented in S2 Fig.

Primary outcomes (See Table 2, S1 and S2 Figs, and S4 Table)

**Table 2:** The result of the meta-analysis on each outcome.

		Random effects model			
			Analysis I		
	Outcome		p value	SMD (95%CI)	I^2^(%)
Primary outcome	Autism general symptoms	〇	0.06	-0.31[-0.63,0.01]	21
Secondary outcomes	Developmental quotient	◎	0.02*	0.36[0.05,0.66]	20
	Developmental quotient (sensitivity analysis)	〇	0.07	0.31[-0.02,0.65]	26
	Expressive language	〇	0.23	0.11[-0.07,0.30]	0
	Expressive language (sensitivity analysis)	〇	0.18	0.13[-0.06,0.33]	0
	Receptive language		0.30	0.12[-0.11,034]	24
	Receptive language (sensitivity analysis)		0.30	0.12[-0.11,034]	24
	Reciprocity of social intercation towards others	◎	<0.01*	0.53[0.29,0.78]	18
	Reciprocity of social intercation towards others (Sensitivity analysis)		<0.01*	0.53[0.29,0.78]	18
	Adaptive behaviour		0.69	-0.04[-0.23,0.15]	0
	Adaptive behaviour (Sensitivity analysis)		0.69	-0.04[-0.23,0.15]	0
Other outcomes	Qualitative impairment in social interaction		0.16	-0.15[-0.40,0.10]	0
	Qualitative impairment in communication		0.85	-0.03[-0.35,0.29]	N/A
	Restricted repetitive and stereotyped patterns of behaviour, interests, and activities		0.47	-0.13[-0.50,0.23]	51
	Initiating joint attention	〇	0.08	0.40[-0.04,0.84]	68
	Responding to joint attention		0.10	0.63[-0.14,1.39]	97
	Imitation	〇	0.18	0.54[-0.25,1.33]	62
	Symbolic play		N/A		
	Functional play	〇	N/A		
	Parental synchrony	◎	<0.01*	0.99[0.70,1.29]	0
	Parenting stress		0.15	-0.30[-0.69,0.10]	0

p value indicates the value of the test of overall synthesis. SMD indicates standard mean difference of the overall synthesis effect. 95% CI indicates the 95% confidence interval of the standard mean difference of the overall synthesis.

*, **, and *** indicate statistically significant effectiveness (p<0.05, p<0.01, and P<0.001, respectively) in the analysis. 〇 indicates the outcome did not show significant effectiveness in the overall synthesis, but showed significant effectiveness in the sensitivity analysis. ◎ indicates the outcome showed significant effectiveness in both the overall synthesis and its sensitivity analysis (i.e. Analysis I and II, Analysis III and IV). N/A indicates the analysis with overall synthesis could not be performed because only one study measured the outcome. "With excluded studies" indicates the results of sensitivitty anslyses in which the excluded in the data analyses were included.

1.1. Autism general symptoms

We rated the overall quality of the evidence as ‘moderate’ (See Table 1).

Three studies, two social-communication focused studies [19, 45] and a multimodal developmental model study [20] (227 children), were included. There was no behavioural model study with outcome measures for ‘autism general symptoms’. There was no significant difference in the effectiveness of interventions between the communication-focus model and multimodal developmental model (p = 0.83). The overall effect of the two intervention models did not show a significant improvement in the intervention groups compared to the control groups (Standard Mean Difference (SMD) = -0.31, 95% confidential interval (CI) = -0.63 to 0.01, p value = 0.06). There was no significant heterogeneity in the intervention effect indicated (I^2^ = 21%).

**Secondary outcomes** (See Table 2, S1 and S2 Figs, and S4 and S6 Tables)

2.1. Developmental quotient (See Table 2 and S1 Fig, 2.1)

We rated the overall quality of the evidence as ‘moderate’ (See Table 1).

Five studies (one behavioural model study [18], one social-communication focused model study [48], three multimodal developmental model studies [20, 53, 64]; 232 children) were included. These showed a significant improvement in the intervention groups, compared to the control groups in the overall analyses (p = 0.02, SMD = 0.36, 95%CI = 0.05 to 0.66). There was no significant difference (p = 0.34) among the three models. Its heterogeneity was insignificant (I^2^ = 20%).

2.2. Expressive language (See S1 Fig, 2.2 and Table 2)

We rated the overall quality of the evidence as ‘moderate’ (See Table 1).

Eight studies (one behavioural model study [18], five social-communication focused model studies [19, 45, 48, 55, 68], two multimodal developmental model studies [20, 64]; 457 children) were included. There was no significant difference among the models (p = 0.80). A significant improvement was not shown in the overall synthesis (p = 0.23, SMD = 0.11, 95% CI = -0.07 to 0.30). Its heterogeneity was insignificant (I^2^ = 0%).

2.3 Receptive language (See Table 2 and S1 Fig, 2.3)

We rated the overall quality of the evidence as ‘moderate’ (See Table 1).

Eight studies (one behavioural model study [18], five social-communication focused studies [19, 45, 48, 55, 68], two multimodal developmental studies [20, 64]; 457 children) were included. There was no significant differences in effect among the models (p = 0.63). A significant improvement was not shown in the overall synthesis (p = 0.30, SMD = 0.12, 95% CI = -0.11 to 0.34). Its heterogeneity was not significant (I^2^ = 24%).

2.4 Reciprocity of social interaction towards others (See Table 2 and S1 Fig, 2.4)

We rated the overall quality of the evidence as ‘high’ (See Table 1).

Eight studies (no behavioural model study, seven social-communication focused model studies [19, 45, 50, 55, 60, 68, 77], one multimodal developmental model study [47]; 380 children) were included. Significant improvement was shown in the overall synthesis (p < 0.001, SMD = 0.53, 95% CI = 0.29 to 0.78). There was no significant difference in effect between social-communication focused and multimodal developmental models (p = 0.12). The heterogeneity was insignificant (I^2^ = 18%).

2.5 Adaptive behaviour (See Table 2 and S1 Fig, 2.5)

We rated the overall quality of the evidence as ‘moderate’ (See Table 1).

Seven studies (seven programmes) (one behavioural model study [18] (two programmes), two social-communication focused model studies [19, 52] (two programmes), four multimodal developmental model studies (four programmes); 414 children were included. There was no significant difference in effect among the models (p = 0.95). A significant improvement was not shown in the overall synthesis (p = 0.69). Its heterogeneity was not significant (I^2^ = 0%).

See Table 2, S1 and S2 Figs, S4 and S5 Tables for the results of the other outcomes: 3.1–3.10.

There were no significant differences among the models for the outcomes: 3.1–3.10. ‘Parental synchrony’ showed significant improvement in the overall syntheses (p < 0.01). ‘Initiating joint attention’ did not show significant effectiveness (p = 0.08). ‘Responding to joint attention’ did not show significant effectiveness (p = 0.10). ‘Imitation’ also did not show significant effectiveness (p = 0.18). Functional play showed significant effectiveness (p < 0.01), but meta-analyses for functional play could not be performed because only one study measured the outcome.

### Sensitivity analysis

Autism general symptoms (See S2 Fig and S5, S6 and S7 Tables)

Seven studies, three social-communication focused model studies [19, 45, 48] and four multimodal developmental model studies [20, 53, 61, 69], were included in Analysis II and IV.

The sensitivity analyses of Analysis II, III, and IV showed significant effects although Analysis I did not. (Analysis II: SMD = -0.30, 95%CI = -0.50 to -0.09, p value<0.01, Analysis III: SMD = -0.27, 95%CI = -0.53 to -0.01, p value = 0.04, Analysis IV: SMD = -0.30, 95%CI = -0.50 to -0.09, p value<0.01).

2.1. Developmental quotient (See S2 Fig and S5, S6 and S7 Tables)

Nine studies (one behavioural model study [18], two social-communication focused studies [48, 59], six multimodal developmental model studies [20, 53, 61, 64, 70, 72]) were included in Analysis III. The results of the sensitivity analyses, Analysis II, III, and IV, showed significant effects (Analysis II: p = 0.02, SMD = 0.22, 95%CI = 0.04 to 0.41; Analysis III: p = 0.01, SMD = 0.33, 95%CI = 0.07 to 0.52; Analysis IV: p = 0.02, SMD = 0.22, 95%CI = 0.04 to 0.41), which were congruent with the results of Analysis I.

[Analyses excluding studies with significant baseline imbalance]

Since Drew *et al*.’s study [48] had significant baseline imbalance (Mean (SD): Experimental group (Exp.) = 88.1 (11.2), Control group (Cont.) = 66.0 (16.5)), we performed sensitivity analyses removing the study in Analysis I, II, III, and IV. The sensitivity analyses altered the results, in terms of statistical significance on this outcome for Analysis I (p = 0.07, SMD = 0.31, 95%CI = -0.02 to 0.65), but did not alter the results for Analysis II, III, and IV (Analysis II: p = 0.04, SMD = 0.20, 95%CI = 0.00 to 0.39; Analysis III: p = 0.04, SMD = 0.29, 95%CI = 0.01 to 0.56; Analysis IV: p = 0.04, SMD = 0.20, 95%CI = 0.00 to 0.39) (See S6 and S7 Tables).

2.2. Expressive language (See S2 Fig and S5, S6 and S7 Tables)

There was no significant difference among the models (Analysis II: p = 0.79, Analysis III: p = 0.80, Analysis IV: p = 0.79). Sensitivity analyses, which comprised all included studies using both a random and fixed effects model (Analysis II: p = 0.03, SMD = 0.15, 95% CI = 0.01 to 0.29; Analysis IV: p = 0.03, SMD = 0.15, 95% CI = 0.01 to 0.29), showed significant effects on this outcome, although analyses excluded studies at a high risk of bias using both models (Analysis III: p = 0.23, SMD = 0.11, 95% CI = -0.07 to 0.30). The heterogeneity was insignificant (I^2^ = 0% in Analysis II, III, and IV).

[Analyses excluding studies with significant baseline imbalance]

Since there was significant baseline imbalance in Kaale *et al*. 2012’s study [55] (Mean (Min-Max): Experimental group (Exp.) = 14.2 (9.17–19.3), Control group (Cont.) = 20.1 (13.5–26.8)) and the home-based program (Mean (SD): Exp. = 3.4 (8.3), Cont. = 6.0 (10.9)) and the centre-based programme (Mean (SD): Exp. = 8.2 (16.6), Cont. = 6.0 (10.9)) of Roberts *et al*. 2011’s study [63], we performed sensitivity analyses for Analysis I, II, III, and IV by removing those studies. The sensitivity analyses did not alter the results in terms of statistical significance (Analysis I: p = 0.18, Analysis II: p = 0.02, Analysis III: p = 0.18, Analysis IV: p = 0.02) (See S6 and S7 Tables).

2.3 Receptive language (See S2 Fig and S5, S6 and S7 Tables)

15 studies (fifteen programmes) (one behavioural model study [18] (one programme), eight social-communication focused model studies [19, 45, 46, 48, 49, 55, 65, 68] (eight programmes), five multimodal developmental model studies [20, 63, 64, 70, 72] (six programmes)) were included in Analysis II and IV. There was no significant differences in effect among the models (Analysis II: p = 0.52, Analysis III: p = 0.55, Analysis IV: p = 0.40). The sensitivity analyses for the overall syntheses did not alter the results, in terms of significant effect on this outcome (Analysis II: p = 0.55, SMD = 0.05, 95% CI = -0.11 to 0.21; Analysis III: p = 0.33, SMD = 0.09, 95% CI = -0.09 to 0.28; Analysis IV: p = 0.55, SMD = 0.04, 95% CI = -0.11 to 0.19). The heterogeneity was not significant (I^2^ = 11% in Analysis II and IV; 24% in Analysis III).

[Analyses excluding studies with significant baseline imbalance]

Since there was significant baseline imbalance in the home-based programme of Roberts *et al*. 2011’s study [63] (Mean (SD): Exp. = 4.2 (9.2), Cont. = 7.2 (15.2)), Tonge *et al*. 2014 [70] (Mean (SD): Exp. = 9.80 (15.72), Cont. = 14.47 (21.32)), and Welterlin *et al*. 2012 [72] (Mean (SD): Exp. = 9.9 (4.4), Cont. = 7.3 (4.2))’ studies, we performed sensitivity analyses for Analysis I, II, III, and IV by removing those studies. The sensitivity analyses did not alter the results in terms of statistical significance (Analysis I: p = 0.30, Analysis II: p = 0.25, Analysis III: p = 0.33, Analysis IV: p = 0.27,) (See S6 and S7 Tables).

2.4 Reciprocity of social interaction towards others (See S2 Fig and S5, S6 and S7 Tables)

14 studies (no behavioural model study, 11 social-communication focused model studies [19, 45, 50, 55–57, 59, 66, 68, 71, 75], three multimodal developmental model study [47, 61, 72]) were included in Analysis II and IV. There was no significant difference in effect between social-communication focused and multimodal developmental models (Analysis II: p = 0.33, Analysis III: p = 0.12, Analysis IV: p = 0.35). The results of the sensitivity analyses, Analysis II, III, and IV for the overall synthesis were congruent with that of Analysis I in terms of statistical significance of effects on this outcome (Analysis II: p < 0.001, SMD = 0.44, 95% CI = 0.24 to 0.64; Analysis III: p < 0.001, SMD = 0.51, 95% CI = 0.31 to 0.72; Analysis IV: p < 0.001, SMD = 0.43, 95% CI = 0.26 to 0.61). The heterogeneity was insignificant (I^2^ = 18% in Analysis II, III, and IV).

[Analyses excluding studies with significant baseline imbalance]

Since there was significant baseline imbalance in Landa *et al*. 2011 [59] (Mean (SD): Exp. = 2.42 (2.93), Cont. = 3.54 (3.56)) and Venker *et al*. 2012 [71] (Mean (SD): Exp. = 0.29 (0.49), Cont. = 2.00 (2.24))’ studies, we performed sensitivity analyses for Analysis I, II, III, and IV by removing those studies. The sensitivity analyses did not alter the results in terms of statistical significance (Analysis I: p < 0.01, Analysis II: p < 0.001, Analysis III: p < 0.01, Analysis IV: p < 0.001) (See Appendix 9).

2.5 Adaptive behaviour (See S2 Fig and S5, S6 and S7 Tables)

Nine studies (ten programmes) (two behavioural model studies [18, 62] (two programmes), two social-communication focused model studies [19, 52] (two programmes), five multimodal developmental model studies [20, 47, 50, 63, 64] (six programmes)) were included in Analysis II, and IV. There was no significant difference in effect among the models (Analysis II: p = 0.88, Analysis III: p = 0.89, Analysis IV: p = 0.76). The results of the sensitivity analyses, Analysis II, III, and IV, for the overall syntheses were congruent with that of Analysis I, in terms of no statistical significance. (Analysis II: p = 0.60, Analysis III: p = 0.69, Analysis IV: p = 1.00). The heterogeneity was not significant (I^2^ = 0% in Analysis II, III, and IV).

[Analyses excluding studies with significant baseline imbalance]

Since there was significant baseline imbalance in the centre-based program of Roberts *et al*. 2011’s study [63] (Mean (SD): Exp. = 58.5 (20.4), Cont. = 43.9 (21.9)), we performed sensitivity analyses for Analysis I, II, III, and IV by removing those studies. The sensitivity analyses did not alter the results in terms of statistical significance (Analysis I: p = 0.69, Analysis II: p = 0.45, Analysis III: p = 0.69, Analysis IV: p = 0.78) (See S6 and S7 Tables).

See S2 Fig and S5, S6 and S7 Tables for the results of the other outcomes: 3.1–3.10. There was no significant difference among the models for the outcomes: 3.1–3.10. ‘Parental synchrony’ showed significant improvement in all of the overall syntheses, Analysis I to IV (Analysis II: p < 0.01, Analysis III: p < 0.001, Analysis IV: p < 0.001). ‘Initiating joint attention’ showed significant effectiveness in Analysis II, III, and IV (p < 0.01), although Analysis I did not. ‘Responding to joint attention’ showed significant effectiveness in the fixed model analyses (Analysis III and IV: p < 0.001), but did not show significant effectiveness in the random effects model analyses (Analysis II: p = 0.11). ‘Imitation’ showed significant effectiveness in the analyses of all the included studies, both random and fixed effects models (Analysis II: p = 0.02, Analysis IV: p < 0.01), but did not in the analyses excluding studies at a high risk of bias using both random and fixed effects models (Analysis III: p = 0.05). Functional play also showed significant effectiveness in the sensitivity analyses with including studies using both random and fixed effects models (Analysis II and IV: p < 0.01), but a meta-analysis could not be performed in Analysis III because only one study measured the outcome.

## Discussion

### Principal findings

The present study compared three intervention models (behavioural model, social-communication focused model, and multimodal developmental model) for pre-school children with ASD. The present study did not suggest that the social-communication focused model and multimodal developmental model have significant differences in effects on autism general symptoms (there was no available study in the behaviour model category to include in the analysis). The results also did not suggest that the three models have significant differences in effects on the other secondary outcomes (‘developmental quotient’, ‘expressive language’, ‘receptive language’, ‘reciprocity of social interaction towards others’, and ‘adaptive behaviour’).

This study also performed a combined meta-analysis of all three models to investigate the overall effectiveness of the intervention programmes for pre-school children with ASD, and observed no significant effects for the outcomes of ‘autism general symptoms’, ‘developmental quotient’, ‘expressive language’, ‘receptive language’, and ‘adaptive behaviour’. However, significant effects were shown on ‘reciprocity of social interaction towards others’, and ‘parental synchrony’.

### Strengths and weaknesses of the study

As far as we know, this study is the largest meta-analysis conducted to date, in terms of the number of included RCTs of intervention programmes for children with ASD. Although several meta-analyses have assessed intervention programmes for children with ASD [14, 41, 42, 78–80], most of these studies [14, 41, 78–80] included non-randomized controlled trials, which can introduce significant bias in the data analysis. Additionally, we applied rigorous exclusion criteria for the RCTs analysed in our study. Thus, the quality of evidence for all outcomes listed in the ‘Summary of findings table’ were ‘moderate’ or ‘high’, resulting in more reliable evidence than that produced by previous studies [41, 42] (See Table 1).

The outcomes measured vary according to the intervention model, which means that there are only a limited number of studies in which the outcomes can be compared across the three models. Behavioural models in particular could not be included in the meta-analysis for many outcomes because very few RCTs exist for behavioural models.

The small number of available studies has limited the ability to make inferences comparing the three models and investigating each type of intervention’s strengths and weaknesses with respect to important outcomes.

### Comparison with other studies

This is the first systematic review and meta-analysis to compare different models of early interventions for pre-school children with ASD. One systematic review conducted a meta-analysis only on parent-mediated intervention programmes [42], but in our study, we looked at therapist-implemented, parent-mediated, teacher-implemented, or combined intervention types. In the combined meta-analysis of all intervention types, we also paid attention to heterogeneity in the analyses, which was found to be insignificant for many of the outcomes (except for the outcomes of ‘responding to joint attention’ and ‘imitation’). Another previous systematic review analysed the efficacy of interventions based on the Theory of Mind cognitive model for ASD [41] but contained none of the multimodal developmental model studies. Since interventions designed based on the Theory of Mind cognitive model use techniques that overlap with those used in joint attention and social communication [49, 55, 56, 59, 65, 73, 74] as well as in imitation training intervention programmes [52], the Theory of Mind cognitive model was defined as a social-communication focused model in our study.

With regard to the primary outcome of ‘autism general symptoms’ in this study, we conclude that there is not enough evidence to support the effectiveness of early interventions for treating ASD. However, the results of the sensitivity analyses, Analysis II, III, and IV, suggest that there is a possibility of effectiveness. The discrepancy in the results between Analysis II and Analysis I, III, and IV could be due to a lack of power in detecting effectiveness in a small number of studies in Analysis II. A previous systematic review suggested that there was low quality evidence for the effectiveness of the intervention programmes [81]. The review suggested the possibility of the interventions having an effect on ‘autism general symptoms’ based on seven studies [20, 53, 61, 70, 82–84]. Four [20, 53, 61, 70] of the studies were included in the current review. The other three studies were not eligible for this review because they did not focus on pre-school children [83], were not RCTs [82], or the control conditions were not treatment as usual [84]. The previous review also did not perform an overall synthesis of the studies. The present study suggests that the intervention programmes have an effect on ‘autism general symptoms’ based on an assessment of a larger number of studies and using more rigorous analyses, including overall syntheses and sensitivity analyses. However, due to the possibility of Analysis II lacking sufficient power, further research is needed using autism specific measures to accumulate evidence of the effect on ‘autism general symptoms’.

Regarding the discrepancy between the results in the overall synthesis and the sensitivity analyses for ‘developmental quotient’, we believe that the results of the sensitivity analysis that excluded Drew *et al*.’s study [48], which showed a non-significant effect on this outcome, offers more robust evidence than the results of Analysis I due to its bias of baseline imbalance. The results of previous meta-analysis studies on the outcome of ‘developmental quotient’ have not been consistent. A previous meta-analysis [85] classified early intervention studies for children with ASD into four types to investigate their effectiveness on IQ and academic skills. These four categories were: behavioural interventions [18, 20, 64], educational interventions [84], parent training [42, 61, 64, 83], and social-communication intervention [46, 86]. Two of the studies [86, 87] were not included in our meta-analysis because their control conditions were not treatment as usual [84]. The study observed no evidence of a statistically significant effect of early intensive behavioural interventions on child development. The meta-analysis suggested that educational interventions had a statistically significant effect based on the results from a single study. It also mentions that parent training [48, 69, 72, 87] and social-communication interventions did not show a statistically significant effect. Although the study investigated the effectiveness of each type of intervention separately, it did not perform an overall synthesis of all four types of studies. Other previous meta-analysis studies on behavioural models (most of which were of non-RCT studies) suggested their significant effectiveness in improving IQ [15, 79, 80, 88], but one study did not [14]. One meta-analysis of controlled studies of the TEACCH programme, which can be classified as a multimodal developmental model, also revealed significant effectiveness in improving IQ [89]. Our study used the outcome ‘developmental quotient’, which is not limited to IQ but instead covers a wide range of factors related to child development. From the results of the overall synthesis and the sensitivity analyses, we conclude that there is not enough evidence of an effect on ‘developmental quotient’, but there could still be the possibility of an effect on this outcome.

The results on ‘expressive language’ did not show significant effectiveness of early interventions on this outcome. However, the results of Analyses II and IV suggest that early interventions could enhance ‘expressive language’. These significant results are consistent with a previous study [79]. Some of the studies included in the analysis suggest that children with baseline lower language abilities might show larger gains following an intervention [66, 69, 70]. The baseline language level was not considered in the present analysis, but it might have had some effects on ‘expressive language’ outcome. Subsequent meta-analyses into language abilities should consider a child’s baseline language levels. We suggest there is a possibility of effectiveness for early interventions on ‘expressive language’.

The results on ‘reciprocity of social interaction towards others’ throughout Analysis I to IV suggest early interventions may have significant effectiveness on this outcome. The results are consistent with the previous meta-analyses on the social-communication focused model [41, 42]. The interventions in Aldred *et al*. [45] and Green *et al*. [19] focused on enhancing parent-child interaction through parental sensitivity and parental synchrony. They also demonstrated that parental synchrony and sensitivity play a role in helping mediators enhance the communication and social interaction of children with ASD [90]. Parental synchrony and sensitivity can be thought to have played an important role in the effectiveness of enhancing children’s ‘reciprocity of social interaction toward others’ not only in Aldred *et al*. [45] and Green *et al*. [19], but also in the other studies (Analyses I and III [47, 50, 55, 56, 60, 68], Analyses I and IV [47, 50, 55–57, 59–61, 66–72]). However, many of the included studies’ programmes were parent-implemented treatments. The outcome, “reciprocity of social interaction toward others” is usually measured in parent-child sessions, which can be a dependent variable. The effectiveness of this outcome can be explained in a measurement procedure led by the interventionist. The outcome had been measured in parent-child sessions in many of the included studies of the meta-analyses. Parents participating in the intervention group of those studies would have been taught to intensively elicit the child’s part of the reciprocal exchange, and some in the control group did not. Since this outcome could be a dependent variable that might be context-bound to interactions with the child’s parent, we cannot conclude the children of the intervention group had gained a generalized skill to engage in reciprocal interactions with others. Thus, further studies are needed for parent-mediated intervention studies, which measured the effects of “reciprocity of social interaction toward others” via parent-child dyad settings, and sought to discover whether the effects can really be generalized to include both parents and others, using validated measurements and settings of third person-child dyad.

The present study also demonstrated the significant effectiveness of early interventions for ‘parental synchrony’, which was consistent with the previous study [42]. We suggest that ‘parental synchrony’ should be considered as an essential and promising effective target for early interventions for children with ASD.

The results did not suggest significant effectiveness of early interventions for pre-school children with ASD on ‘initiating joint attention’. However, there was a discrepancy in the results between Analysis I and its sensitivity analyses (Analysis II, III, and IV). The sensitivity analyses showed significant effectiveness on ‘initiating joint attention’. One previous meta-analysis made a similar conclusion [41]. Since the meta-analyses in Analysis I and III had high heterogeneity, we consider the results to be unreliable. In Analysis I on ‘initiating joint attention’, two studies [52, 55] used the Early Social Communication Scales (ESCS) measure, whereas the other studies relied on original measurements [47, 56, 60, 64]. Thus, we conclude that there is not enough evidence of the effectiveness of early interventions on ‘initiating joint attention’, but there is a possibility of its effectiveness if an analysis is performed on more RCTs, which use common measurements for the outcome.

The present study did not show effectiveness on ‘responding to joint attention’. There were also discrepancies in the results for ‘responding to joint attention’ between the random effects model analyses (Analysis I and II) and the fixed effects model analyses (Analysis III and IV). However, all of these analyses had very high heterogeneity. We regard the results of the significant effect in Analysis III and IV as unreliable. In Analysis II and IV, the ESCS was used in one study [57], and the others [56, 62, 64, 65] used their original measurements. The effectiveness of interventions on ‘responding to joint attention’ should be assessed in further meta-analyses on studies that use common measurements.

Regarding ‘imitation’, the present study did not show the effectiveness of early interventions, but this could be due to a lack of a sufficient number of studies and due to the high heterogeneity of included studies in the analysis. In one previous study, a meta-analysis was not performed because of measurement heterogeneity [41]. The meta-analysis for the outcome in this study also had high heterogeneity [22]. Children with ASD show significant impairment in their ‘imitation’ abilities [91, 92]. Enhancing these skills is expected to assist in improving a child’s social development [93]. The effectiveness of early intervention on ‘imitation’ also should be assessed by further meta-analyses on studies that use common measurements.

The lack of a significant effect on ‘autism general symptoms’, ‘developmental quotient’, ‘expressive language’, ‘receptive language’, and ‘adaptive behaviour’ observed in the analysis suggests that early interventions for ASD may not have an effect on those outcomes in the short term, i.e. in the intervention periods assessed by the RCTs. In Green *et al*.’s study [19], early interventions did not show significant effectiveness for the reduction of autism symptoms, but showed a clear benefit for parent-child dyadic social communication (reciprocity of social interaction towards parent and parental synchrony). Their long-term follow-up study, however, demonstrated that the early intervention program for ASD was significantly effective in reducing autism symptoms [94]. Further meta-analyses looking at long-term follow-up studies of early interventions for ASD are needed to investigate the long-term effects on outcomes that did not show a significant change in the overall syntheses of this study.

Since many of the studies comprised multiple dependent variables that were analyzed, there was non-independence of effect sizes, which can affect confidence interval around the summary effect sizes. This can result in Type I error rate inflation. Type I errors are also common in underpowered meta-analyses [95]. These are limitations of our study. However, as the precision decreases with increasing heterogeneity, confidential interval will widen correspondingly [96]. The random effects model can be thought to be appropriate to control the estimations of the average effects for the outcomes under the conditions, such as multiple dependent variables, sparse data, small number of included studies, and small sample sizes [97, 98]. Thus, we prioritized the random effects model analysis, rather than the fixed effects model for the main analysis in this study.

### Implications for clinicians and policymakers

The lack of power to detect clinically meaningful differences in treatment outcomes among the three categorical models (behavioural model, social-communication focused model, and multimodal developmental model) means that we cannot single out any of the three models as being most effective. There is currently not enough evidence early interventions for children with ASD reduce their autism severity. Nonetheless, the results of the present study suggest that there is potential for their effectiveness. We suggest clinicians consider, at least, the outcomes of ‘reciprocity of social interaction towards others’, and ‘parental synchrony’, as promising targets for interventions, and should work to enhance these abilities in pre-school children with ASD. The present study also suggested that intervention programmes in general can enhance parent–child interactions (shown as improvements in those two outcomes). We recommend that parents are involved in intervention programmes, to enhance not only their child’s development but also parent-child interactions.

### Unanswered questions and future research

Further research is needed into the classification of intervention programmes based on important components of effectiveness. In addition, we still do not know which type of intervention programmes is most effective. Further research is needed to demonstrate the comparative benefits of different approaches, possibly using different frameworks or measuring different outcomes to those in the present study.

This study further demonstrated that only a few studies assessed common outcomes, reflecting differences in the targets and background theories of intervention studies. No uniform standard of measurements exist to assess the effects of interventions on children with ASD, which confounds the problem of comparing and appraising intervention programmes and their outcomes. Future research into intervention programmes for children with ASD should use comprehensive estimation using common standard measurements with appropriate sensitivity and specificity for assessing the effectiveness of interventions for ASD. In addition, none of the behavioural model studies assessed ‘autism general symptoms’. Further research is needed into the effectiveness of behavioural model interventions for treating children with ASD using rigorous RCT methods. Comprehensive assessments [17], which can be applied to various types of ASD intervention studies, should also be developed.

Our analyses were performed based on Howlin’s classification (i.e. behavioural model, social-communication focused model, and multimodal developmental model). There was no significant difference in the intervention effectiveness among the three models. However, a more valid classification might exist that would be able to elucidate the differences in intervention effectiveness.

This study did not distinguish between “core autism” and “spectrum condition” in the analyses. Further studies will need to investigate the characteristics of children who will most benefit from different intervention types.

This study only investigated pre-school children and it is unclear whether intervention programmes that target children older than six years of age could also enhance their cognitive development.

Among the included studies in the meta-analysis, some of the studies were parent-mediated, whereas others directly approach children with ASD. Since the intervention approaches were different, the parents’ abilities might have influenced the outcomes. Further studies targeting therapy approaches (parent-mediated approach vs. direct approach) are needed. Some short-duration intervention programmes, such as Nedft *et al*.’s study [99], which used DVDs in the intervention programme, were excluded and their long-term effects should be investigated.

There is a possibility that combined therapies are effective for treating children with ASD. In fact, interventions under the multimodal developmental model contain many targets and combined concepts of previous studies. Lim *et al*. demonstrated that music therapy in addition to Applied Behaviour Analysis Verbal Behaviour (ABA VB) is superior to only ABA VB to enhance expressive language[100]. Due to our exclusion criteria, we did not include these studies in our meta-analysis. Further research is needed into the effectiveness of combined therapies.

## Conclusion

The small number of studies included in the present study limited the ability to make inferences when comparing behavioural, social-communication focused, and multimodal developmental models of interventions for children with ASD, and investigating the strengths and weaknesses of each type of intervention with respect to important outcomes. Since the outcome of ‘reciprocity of social interaction towards others’ can be a dependent variable which is usually measured in a context-bound setting with the child’s parent, we cannot conclude the interventions for pre-school children with ASD have significant effects on a generalized skill to engage in reciprocal interactions with others. However, the outcomes of ‘reciprocity of social interaction towards others’ and ‘parental synchrony’ may be promising targets for interventions involving pre-school children with ASD.

## Supporting information

S1 Fig Forest plots of Analysis I, which used random effects model with 14 studies. (PDF)

S2 Fig Forest plots of Analysis I, which used random effects model with 29 studies. (PDF)

S1 Table Excluded studies. (PDF)

S2 Table Characteristics of included studies. (PDF)

S3 Table Outcome measure list used in the included studies for the data synthesis. (PDF)

S4 Table Comparisons of the results of the meta-analyses with those of the sensitivity analyses. (PDF)

S5 Table Comparisons of each model condition versus control condition. (PDF)

S6 Table Sensitivity analyses of the outcomes that were with significant baseline imbalances. (PDF)

S7 Table Comparison of the effect among Analysis I, II, III, and IV on each outcome in terms of statistical significance. (PDF)

S8 Table PRISMA 2009 checklist. (PDF)

S1 Appendix Search strategy. (PDF)

S2 Appendix Study protocol. (PDF)
