# Supplementary material for: A systematic review and meta-analysis of comprehensive interventions for pre-school children with autism spectrum disorder (ASD)
Source: PLoS One. 2017 Dec 6;12(12):e0186502. doi: 10.1371/journal.pone.0186502 (PMC5718481; doi:10.1371/journal.pone.0186502)
Supplement: S2 Fig — (PDF) [file pone.0186502.s002.pdf]

S2 Fig. Forest plots of Analysis II, which used random effects model with the 29 studies

: low risk of bias, : unclear risk of bias, : high risk of bias

1.1. Autism general symptoms (Analysis II)

Study or Subgroup	Experimental Mean	Experimental SD	Experimental Total	Control Mean	Control SD	Control Total	Weight	Std. Mean Difference IV, Random, 95% CI	Random sequence generation (selection bias)
**1.2.1 Behavioural intervention versus TAU**									
**Subtotal (95% CI)**			**0**			**0**		**Not estimable**	
Heterogeneity: Not applicable									
Test for overall effect: Not applicable									
**1.2.2 Social-communication focused intervention versus TAU**									
Drew 2002	18.3	4.9	12	20.1	4.3	12	6.2%	-0.38 [-1.19, 0.43]	Low risk
Green 2010	15.7	6	77	16.5	5.7	75	40.0%	-0.14 [-0.45, 0.18]	Low risk
Aldred 2004	11.8	6.4	14	16.1	4.2	14	6.8%	-0.77 [-1.54, 0.00]	Low risk
**Subtotal (95% CI)**			**103**			**101**	**53.0%**	**-0.28 [-0.62, 0.05]**	
Heterogeneity: Tau^2 = 0.02; Chi^2 = 2.34, df = 2 (P = 0.31); I^2 = 14%									
Test for overall effect: Z = 1.65 (P = 0.10)									
**1.2.3 Miltimodal developmental intervention versus TAU**									
Jocelyn 1998	48.5	18.4	16	56.5	20.1	19	9.0%	-0.40 [-1.08, 0.27]	Low risk
Dawson 2010	7	1.9	24	7.3	1.8	21	11.8%	-0.16 [-0.75, 0.43]	Low risk
Tonge 2006	35.86	6.14	35	38.73	6.73	35	18.0%	-0.44 [-0.92, 0.03]	Unclear risk
Pajareya 2011	34.5	6.01	16	37.18	7.42	16	8.3%	-0.39 [-1.09, 0.31]	Unclear risk
**Subtotal (95% CI)**			**91**			**91**	**47.0%**	**-0.35 [-0.65, -0.06]**	
Heterogeneity: Tau^2 = 0.00; Chi^2 = 0.58, df = 3 (P = 0.90); I^2 = 0%									
Test for overall effect: Z = 2.36 (P = 0.02)									
**Total (95% CI)**			**194**			**192**	**100.0%**	**-0.30 [-0.50, -0.10]**	
Heterogeneity: Tau^2 = 0.00; Chi^2 = 3.19, df = 6 (P = 0.78); I^2 = 0%									
Test for overall effect: Z = 2.89 (P = 0.004)									
Test for subgroup differences: Chi^2 = 0.10, df = 1 (P = 0.75), I^2 = 0%									

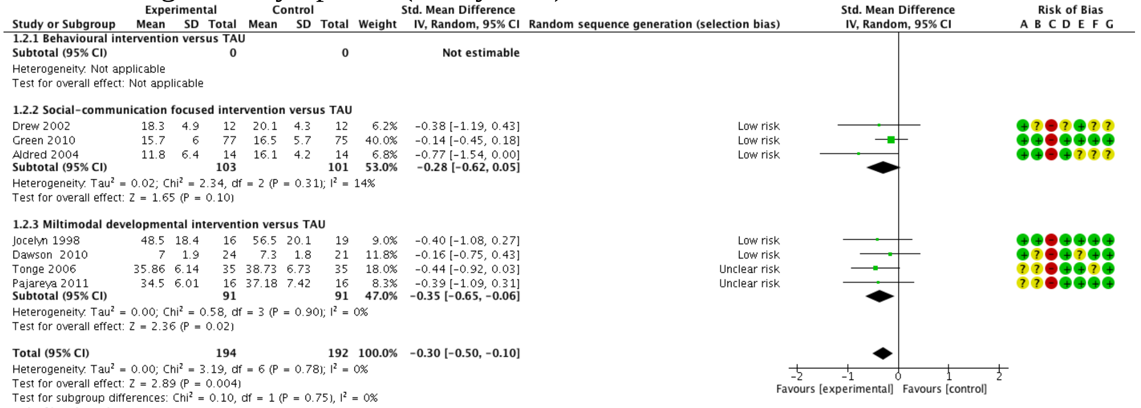

Risk of bias legend
(A) Random sequence generation (selection bias)
(B) Allocation concealment (selection bias)
(C) Blinding of participants and personnel (performance bias)
(D) Blinding of outcome assessment (detection bias)
(E) Incomplete outcome data (attrition bias)
(F) Selective reporting (reporting bias)
(G) Other bias

(Minus direction indicates positive effectiveness.)

2.1 Developmental quotient (Analysis II)

Study or Subgroup	Experimental Mean	Experimental SD	Experimental Total	Control Mean	Control SD	Control Total	Weight	Std. Mean Difference IV, Random, 95% CI	Random sequence generation (selection bias)
**6.1.1 Behavioural intervention versus TAU**									
Smith 2000	66.49	24.08	15	49.67	19.74	13	5.8%	0.74 [-0.04, 1.51]	Low risk
**Subtotal (95% CI)**			**15**			**13**	**5.8%**	**0.74 [-0.04, 1.51]**	
Heterogeneity: Not applicable									
Test for overall effect: Z = 1.87 (P = 0.06)									
**6.1.2 Social-communication focused intervention versus TAU**									
Drew 2002	77.9	14.8	12	66.1	17.1	12	5.0%	0.71 [-0.12, 1.54]	Low risk
Carter 2011	63.88	18.41	25	64.88	13.94	25	11.3%	-0.06 [-0.61, 0.49]	Low risk
Landa 2011	34.44	16.67	24	30.28	16.62	24	10.8%	0.25 [-0.32, 0.81]	Unclear risk
**Subtotal (95% CI)**			**61**			**61**	**27.1%**	**0.22 [-0.18, 0.61]**	
Heterogeneity: Tau^2 = 0.02; Chi^2 = 2.34, df = 2 (P = 0.31); I^2 = 14%									
Test for overall effect: Z = 1.08 (P = 0.28)									
**6.1.3 Multimodal developmental intervention versus TAU**									
Rogers 2012	69.82	17.9	49	67.92	17.93	49	22.1%	0.11 [-0.29, 0.50]	Low risk
Dawson 2010	78.6	24.2	24	66.3	15.3	21	9.7%	0.59 [-0.01, 1.19]	Low risk
Jocelyn 1998	27.2	10.8	16	27	10.4	19	7.8%	0.02 [-0.65, 0.68]	Low risk
Welterlin 2012	63.7	17.4	10	58.1	25	10	4.5%	0.25 [-0.63, 1.13]	Unclear risk
Pajareya 2011	44.81	13.4	16	44	12.88	16	7.2%	0.06 [-0.63, 0.75]	Unclear risk
Tonge 2006	72.18	24.77	35	67.72	28.14	35	15.8%	0.17 [-0.30, 0.64]	Unclear risk
**Subtotal (95% CI)**			**150**			**150**	**67.1%**	**0.18 [-0.04, 0.41]**	
Heterogeneity: Tau^2 = 0.00; Chi^2 = 2.29, df = 5 (P = 0.81); I^2 = 0%									
Test for overall effect: Z = 1.58 (P = 0.11)									
**Total (95% CI)**			**226**			**224**	**100.0%**	**0.22 [0.04, 0.41]**	
Heterogeneity: Tau^2 = 0.00; Chi^2 = 6.45, df = 9 (P = 0.69); I^2 = 0%									
Test for overall effect: Z = 2.33 (P = 0.02)									
Test for subgroup differences: Chi^2 = 1.82, df = 2 (P = 0.40), I^2 = 0%									

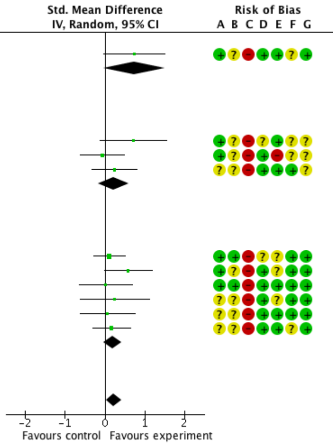

Risk of bias legend
(A) Random sequence generation (selection bias)
(B) Allocation concealment (selection bias)
(C) Blinding of participants and personnel (performance bias)
(D) Blinding of outcome assessment (detection bias)
(E) Incomplete outcome data (attrition bias)
(F) Selective reporting (reporting bias)
(G) Other bias

(Plus direction indicates positive effectiveness.)

2.2. Expressive language (Analysis II)

Study or Subgroup	Experimental Mean	Experimental SD	Experimental Total	Control Mean	Control SD	Control Total	Weight	Std. Mean Difference IV, Random, 95% CI	Random sequence generation (selection bias)
**7.5.1 Behavioural intervention versus TAU**									
Smith 2000	44.53	23.48	15	36.23	21.19	13	3.4%	0.36 [−0.39, 1.11]	Low risk
**Subtotal (95% CI)**			**15**			**13**	**3.4%**	**0.36 [−0.39, 1.11]**	
Heterogeneity: Not applicable									
Test for overall effect: Z = 0.94 (P = 0.35)									
**7.5.2 Social-communication focused intervention versus TAU**									
Kaale 2012	13.7	10.8	32	14	10.87	27	7.3%	−0.03 [−0.54, 0.48]	Low risk
Carter 2011	16.2	7.23	25	16.68	7.88	25	6.2%	−0.06 [−0.62, 0.49]	Low risk
Green 2010	20	11.2	77	20	11.3	75	19.0%	0.00 [−0.32, 0.32]	Low risk
Aldred 2004	199.4	25,606	14	33.1	683	14	3.5%	0.01 [−0.73, 0.75]	Low risk
Goods 2013	14.52	5.38	5	11.95	0.16	6	1.3%	0.65 [−0.58, 1.89]	Low risk
Thompson 2012	51.91	62.88	11	35.4	52.43	10	2.6%	0.27 [−0.59, 1.13]	Low risk
Drew 2002	96.6	118.8	12	44	50.2	12	2.9%	0.56 [−0.26, 1.38]	Low risk
Schertz 2013	33.27	15.79	11	27.17	11.21	12	2.8%	0.43 [−0.40, 1.26]	Unclear risk
Siller 2013	4.02	0.16	31	3.9	0.17	30	7.1%	0.72 [0.20, 1.24]	Unclear risk
Landa 2011	34.08	14.59	24	31.92	13.67	24	6.0%	0.15 [−0.42, 0.72]	Unclear risk
**Subtotal (95% CI)**			**242**			**235**	**58.7%**	**0.17 [−0.01, 0.35]**	
Heterogeneity: $Tau^2 = 0.00$; $Chi^2 = 8.70$, df = 9 (P = 0.47); $I^2 = 0\%$									
Test for overall effect: Z = 1.81 (P = 0.07)									
**7.5.3 Multimodal developmental intervention versus TAU**									
Roberts 2011 Centre-based	7	15.1	26	4.4	8.7	14	4.5%	0.19 [−0.46, 0.84]	Low risk
Roberts 2011 Home-based	2.87	15.1	26	4.4	8.7	14	4.5%	−0.11 [−0.76, 0.54]	Low risk
Rogers 2012	42.27	61.99	49	38.87	73.71	49	12.2%	0.05 [−0.35, 0.45]	Low risk
Dawson 2010	36.6	13.6	24	30	9.2	21	5.4%	0.55 [−0.05, 1.15]	Low risk
Welterlin 2012	16.2	7.1	10	14.2	7.1	10	2.5%	0.27 [−0.61, 1.15]	Unclear risk
Tonge 2006	17.17	17.07	35	18.24	20.65	35	8.7%	−0.06 [−0.52, 0.41]	Unclear risk
**Subtotal (95% CI)**			**170**			**143**	**37.9%**	**0.11 [−0.12, 0.33]**	
Heterogeneity: $Tau^2 = 0.00$; $Chi^2 = 3.30$, df = 5 (P = 0.65); $I^2 = 0\%$									
Test for overall effect: Z = 0.94 (P = 0.35)									
**Total (95% CI)**			**427**			**391**	**100.0%**	**0.15 [0.01, 0.29]**	
Heterogeneity: $Tau^2 = 0.00$; $Chi^2 = 12.47$, df = 16 (P = 0.71); $I^2 = 0\%$									
Test for overall effect: Z = 2.14 (P = 0.03)									
Test for subgroup differences: $Chi^2 = 0.46$, df = 2 (P = 0.79), $I^2 = 0\%$									

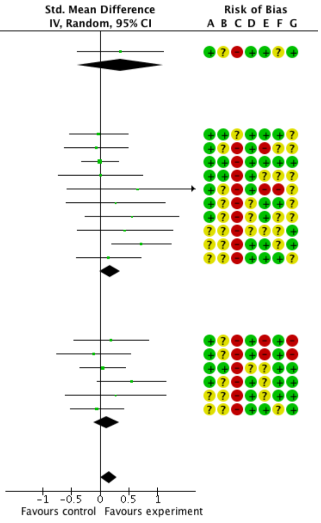

Risk of bias legend
(**A**) Random sequence generation (selection bias)
(**B**) Allocation concealment (selection bias)
(**C**) Blinding of participants and personnel (performance bias)
(**D**) Blinding of outcome assessment (detection bias)
(**E**) Incomplete outcome data (attrition bias)
(**F**) Selective reporting (reporting bias)
(**G**) Other bias

(Plus direction indicates positive effectiveness.)

2.3. Receptive language (Analysis II)

Study or Subgroup	Experimental Mean	Experimental SD	Experimental Total	Control Mean	Control SD	Control Total	Weight	Std. Mean Difference IV, Random, 95% CI
**12.1.1 Behavioural intervention versus TAU**								
Smith 2000	42.87	22.29	15	33	16.86	13	4.2%	0.48 [−0.28, 1.23]
**Subtotal (95% CI)**			**15**			**13**	**4.2%**	**0.48 [−0.28, 1.23]**
Heterogeneity: Not applicable								
Test for overall effect: Z = 1.25 (P = 0.21)								
**12.1.2 Social-communication focused intervention versus TAU**								
Aldred 2004	222.7	40,431	14	146.8	11,426	14	4.4%	0.00 [−0.74, 0.74]
Carter 2011	15.52	6.93	25	17.48	8.33	25	7.4%	−0.25 [−0.81, 0.30]
Drew 2002	176.1	121.9	12	100.3	80.2	12	3.5%	0.71 [−0.12, 1.54]
Goods 2013	14.59	5.36	5	12.05	0.38	6	1.6%	0.65 [−0.59, 1.88]
Green 2010	21.5	13	77	20.3	12.8	75	19.1%	0.09 [−0.23, 0.41]
Kaale 2012	12.9	10.0405	32	14.3	9.606	27	8.6%	−0.14 [−0.65, 0.37]
Schertz 2013	28.27	11.35	11	25.33	8.52	12	3.6%	0.28 [−0.54, 1.11]
Thompson 2012	16.64	7.5	11	15.1	7.25	10	3.3%	0.20 [−0.66, 1.06]
**Subtotal (95% CI)**			**187**			**181**	**51.6%**	**0.07 [−0.13, 0.28]**
Heterogeneity: $Tau^2 = 0.00$; $Chi^2 = 5.45$, df = 7 (P = 0.61); $I^2 = 0\%$								
Test for overall effect: Z = 0.69 (P = 0.49)								
**12.1.3 Multimodal developmental intervention versus TAU**								
Dawson 2010	40	16.3	24	31.5	10.6	21	6.5%	0.60 [−0.00, 1.20]
Roberts 2011 Centre-based	10.5	17.4	26	5.7	12.1	14	5.5%	0.30 [−0.36, 0.95]
Roberts 2011 Home-based	2.6	8.4	27	5.7	12.1	14	5.6%	−0.31 [−0.96, 0.34]
Rogers 2012	12.73	9.11	49	14.77	8.14	49	13.4%	−0.23 [−0.63, 0.16]
Tonge 2006	14.06	19.67	35	19.18	22.17	35	10.0%	−0.24 [−0.71, 0.23]
Welterlin 2012	12	4.7	10	10.9	4.8	10	3.2%	0.22 [−0.66, 1.10]
**Subtotal (95% CI)**			**171**			**143**	**44.2%**	**0.00 [−0.29, 0.30]**
Heterogeneity: $Tau^2 = 0.05$; $Chi^2 = 8.02$, df = 5 (P = 0.16); $I^2 = 38\%$								
Test for overall effect: Z = 0.02 (P = 0.98)								
**Total (95% CI)**			**373**			**337**	**100.0%**	**0.05 [−0.11, 0.21]**
Heterogeneity: $Tau^2 = 0.01$; $Chi^2 = 15.28$, df = 14 (P = 0.36); $I^2 = 8\%$								
Test for overall effect: Z = 0.60 (P = 0.55)								
Test for subgroup differences: $Chi^2 = 1.33$, df = 2 (P = 0.52), $I^2 = 0\%$								

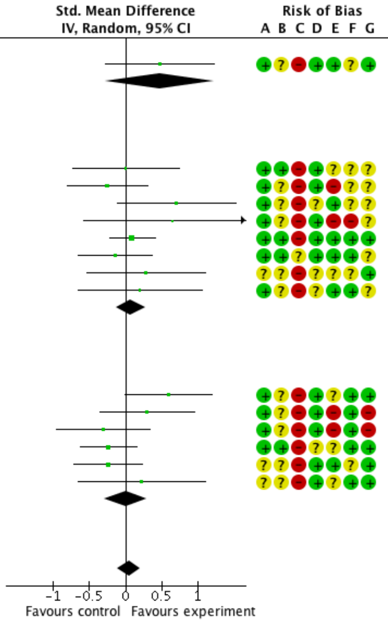

Risk of bias legend
(**A**) Random sequence generation (selection bias)
(**B**) Allocation concealment (selection bias)
(**C**) Blinding of participants and personnel (performance bias)
(**D**) Blinding of outcome assessment (detection bias)
(**E**) Incomplete outcome data (attrition bias)
(**F**) Selective reporting (reporting bias)
(**G**) Other bias

(Plus direction indicates positive effectiveness.)

2.4. Reciprocity of social interaction towards others (Analysis II)

Study or Subgroup	Experimental Mean	Experimental SD	Experimental Total	Control Mean	Control SD	Control Total	Weight	Std. Mean Difference IV, Random, 95% CI	Random sequence generation (selection bias)
**16.5.1 Behavioural intervention versus TAU**									
**Subtotal (95% CI)**			**0**			**0**		**Not estimable**	
Heterogeneity: Not applicable									
Test for overall effect: Not applicable									
**16.5.2 Social-communication focused intervention versus TAU**									
Ichikawa 2013	40.2	5.1	5	39.7	6	6	2.7%	0.08 [−1.11, 1.27]	Low risk
Lawton 2012	5.58	0.81	9	4.11	1.68	7	3.2%	1.10 [0.02, 2.19]	Low risk
Kasari 2010	42.85	19.96	19	27.87	14.01	19	7.6%	0.85 [0.18, 1.52]	Low risk
Aldred 2004	37.6	10.1	14	27.6	16.5	14	6.0%	0.71 [−0.06, 1.48]	Low risk
Green 2010	34.9	19.7	77	26	17.5	75	20.3%	0.47 [0.15, 0.80]	Low risk
Kaale 2012	57.3	22.8	34	49.2	19.9	27	11.5%	0.37 [−0.14, 0.88]	Low risk
Thompson 2012	34.455	7.34	12	23.6	8.06	11	4.3%	1.36 [0.44, 2.29]	Low risk
Venker 2012	9.71	14.08	7	1.86	2.67	7	3.2%	0.73 [−0.37, 1.82]	Unclear risk
Siller 2013	3.83	1.67	31	3.1	1.64	28	11.2%	0.44 [−0.08, 0.95]	Unclear risk
Kim 2008	1.91	3.45	5	0.38	1.01	5	2.4%	0.54 [−0.73, 1.82]	Unclear risk
Landa 2011	4.96	5.48	24	7.57	9.76	24	9.7%	−0.32 [−0.89, 0.25]	Unclear risk
**Subtotal (95% CI)**			**237**			**223**	**82.0%**	**0.50 [0.24, 0.75]**	
Heterogeneity: $Tau^2 = 0.05$; $Chi^2 = 14.76$, df = 10 (P = 0.14); $I^2 = 32\%$									
Test for overall effect: Z = 3.85 (P = 0.0001)									
**16.5.3 Multimodal developmental intervention versus TAU**									
Casenhiser 2011	3.28	0.458	16	3.23	0.43	13	6.5%	0.11 [−0.62, 0.84]	Low risk
Welterlin 2012	18.4	7.3	10	16	5	10	4.6%	0.37 [−0.52, 1.25]	Unclear risk
Pajareya 2011	29.9	16.2	15	25.6	13.3	16	6.8%	0.28 [−0.42, 0.99]	Unclear risk
**Subtotal (95% CI)**			**41**			**39**	**18.0%**	**0.24 [−0.20, 0.68]**	
Heterogeneity: $Tau^2 = 0.00$; $Chi^2 = 0.22$, df = 2 (P = 0.90); $I^2 = 0\%$									
Test for overall effect: Z = 1.07 (P = 0.28)									
**Total (95% CI)**			**278**			**262**	**100.0%**	**0.44 [0.24, 0.64]**	
Heterogeneity: $Tau^2 = 0.03$; $Chi^2 = 15.83$, df = 13 (P = 0.26); $I^2 = 18\%$									
Test for overall effect: Z = 4.25 (P < 0.0001)									
Test for subgroup differences: $Chi^2 = 0.96$, df = 1 (P = 0.33), $I^2 = 0\%$									

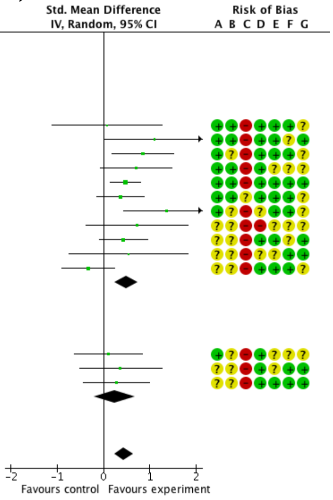

Risk of bias legend
(**A**) Random sequence generation (selection bias)
(**B**) Allocation concealment (selection bias)
(**C**) Blinding of participants and personnel (performance bias)
(**D**) Blinding of outcome assessment (detection bias)
(**E**) Incomplete outcome data (attrition bias)
(**F**) Selective reporting (reporting bias)
(**G**) Other bias

(Plus direction indicates positive effectiveness.)

2.5. Adaptive behaviour (Analysis II)

Study or Subgroup	Experimental Mean	Experimental SD	Experimental Total	Control Mean	Control SD	Control Total	Weight	Std. Mean Difference IV, Random, 95% CI	Random sequence generation (selection bias)
**3.2.1 Behavioural intervention versus TAU**									
Smith 2000	61.19	29.72	15	58.5	16.58	13	8.3%	0.11 [−0.64, 0.85]	Low risk
Reitzel 2013	62.7	6.8	4	63.3	5.7	4	2.9%	−0.08 [−1.47, 1.30]	Low risk
**Subtotal (95% CI)**			**19**			**17**	**11.2%**	**0.06 [−0.59, 0.72]**	
Heterogeneity: $Tau^2 = 0.00$; $Chi^2 = 0.06$, df = 1 (P = 0.81); $I^2 = 0\%$									
Test for overall effect: Z = 0.19 (P = 0.85)									
**3.2.2 Social-communication focused intervention versus TAU**									
Green 2010	60.3	15.2	77	62.8	14.8	75	21.5%	−0.17 [−0.48, 0.15]	Low risk
Ingersoll 2012	101.14	27.98	14	91.08	18.73	13	7.9%	0.41 [−0.36, 1.17]	Low risk
**Subtotal (95% CI)**			**91**			**88**	**29.5%**	**0.01 [−0.51, 0.53]**	
Heterogeneity: $Tau^2 = 0.07$; $Chi^2 = 1.84$, df = 1 (P = 0.18); $I^2 = 46\%$									
Test for overall effect: Z = 0.04 (P = 0.97)									
**3.2.3 Multimodal developmental intervention versus TAU**									
Ichikawa 2013	14.4	4.7	5	12.5	3.2	6	3.7%	0.44 [−0.77, 1.65]	Low risk
Roberts 2011 Centre-based	55.7	19.5	22	42.9	24.3	13	9.0%	0.59 [−0.12, 1.29]	Low risk
Casenhiser 2011	3.48	0.963	16	3.35	0.797	13	8.4%	0.14 [−0.59, 0.87]	Low risk
Roberts 2011 Home-based	52.9	29.3	22	42.9	24.3	13	9.2%	0.35 [−0.34, 1.05]	Low risk
Dawson 2010	68.7	15.9	24	59.1	8.8	21	11.1%	0.72 [0.11, 1.33]	Low risk
Rogers 2012	77.43	9.59	49	80.33	11.34	49	17.9%	−0.27 [−0.67, 0.12]	Low risk
**Subtotal (95% CI)**			**138**			**115**	**59.4%**	**0.27 [−0.11, 0.65]**	
Heterogeneity: $Tau^2 = 0.10$; $Chi^2 = 9.77$, df = 5 (P = 0.08); $I^2 = 49\%$									
Test for overall effect: Z = 1.41 (P = 0.16)									
**Total (95% CI)**			**248**			**220**	**100.0%**	**0.15 [−0.10, 0.39]**	
Heterogeneity: $Tau^2 = 0.05$; $Chi^2 = 13.33$, df = 9 (P = 0.15); $I^2 = 32\%$									
Test for overall effect: Z = 1.17 (P = 0.24)									
Test for subgroup differences: $Chi^2 = 0.74$, df = 2 (P = 0.69), $I^2 = 0\%$									

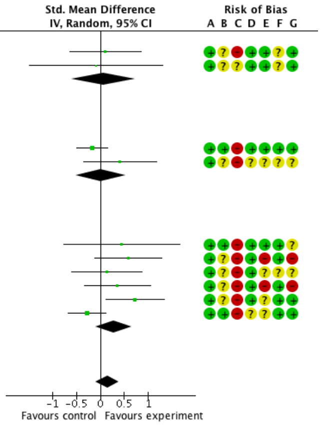

Risk of bias legend
(**A**) Random sequence generation (selection bias)
(**B**) Allocation concealment (selection bias)
(**C**) Blinding of participants and personnel (performance bias)
(**D**) Blinding of outcome assessment (detection bias)
(**E**) Incomplete outcome data (attrition bias)
(**F**) Selective reporting (reporting bias)
(**G**) Other bias

(Plus direction indicates positive effectiveness.)

3.1. Autism symptom: qualitative impairments in social interaction (Analysis II)

Study or Subgroup	Experimental Mean	Experimental SD	Experimental Total	Control Mean	Control SD	Control Total	Weight	Std. Mean Difference IV, Random, 95% CI	Random sequence generation (selection bias)	A	B	C	D	E	F	G
**2.5.1 Behavioural intervention versus TAU**																
**Subtotal (95% CI)**			**0**			**0**		**Not estimable**								
Heterogeneity: Not applicable																
Test for overall effect: Not applicable																
**2.5.2 Social-communication focused intervention versus TAU**																
Drew 2002	18.3	4.9	12	20.1	4.3	12	9.8%	−0.38 [−1.19, 0.43]	Low risk	+	?	−	?	+	?	?
Carter 2011	15.56	4.56	25	13.6	4.89	25	18.4%	0.41 [−0.15, 0.97]	Low risk	+	?	−	+	−	?	?
Green 2010	9.2	3	77	9.8	2.9	75	40.8%	−0.20 [−0.52, 0.12]	Low risk	+	+	−	+	+	+	+
**Subtotal (95% CI)**			**114**			**112**	**69.0%**	**−0.05 [−0.48, 0.39]**								
Heterogeneity: $Tau^2 = 0.07$; $Chi^2 = 3.99$, df = 2 (P = 0.14); $I^2 = 50\%$																
Test for overall effect: Z = 0.21 (P = 0.84)																
**2.5.3 Multimodal developmental intervention versus TAU**																
Rogers 2012	26.61	10.14	49	27.33	10.62	49	31.0%	−0.07 [−0.46, 0.33]	Low risk	+	+	−	?	?	+	+
**Subtotal (95% CI)**			**49**			**49**	**31.0%**	**−0.07 [−0.46, 0.33]**								
Heterogeneity: Not applicable																
Test for overall effect: Z = 0.34 (P = 0.73)																
**Total (95% CI)**			**163**			**161**	**100.0%**	**−0.07 [−0.33, 0.20]**								
Heterogeneity: $Tau^2 = 0.02$; $Chi^2 = 4.00$, df = 3 (P = 0.26); $I^2 = 25\%$																
Test for overall effect: Z = 0.48 (P = 0.63)																
Test for subgroup differences: $Chi^2 = 0.01$, df = 1 (P = 0.94), $I^2 = 0\%$																

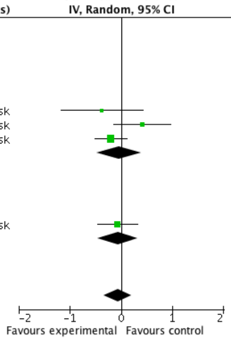

Risk of bias legend
(**A**) Random sequence generation (selection bias)
(**B**) Allocation concealment (selection bias)
(**C**) Blinding of participants and personnel (performance bias)
(**D**) Blinding of outcome assessment (detection bias)
(**E**) Incomplete outcome data (attrition bias)
(**F**) Selective reporting (reporting bias)
(**G**) Other bias

(Minus direction indicates positive effectiveness.)

3.2. Autism symptom: qualitative impairments in communication (Analysis II)

Study or Subgroup	Experimental Mean	Experimental SD	Experimental Total	Control Mean	Control SD	Control Total	Weight	Std. Mean Difference IV, Random, 95% CI	Random sequence generation (selection bias)	A	B	C	D	E	F	G
**3.1.1 Behavioral intervention:autism symptoms versus TAU**																
**Subtotal (95% CI)**			**0**			**0**		**Not estimable**								
Heterogeneity: Not applicable																
Test for overall effect: Not applicable																
**3.1.2 Social-communication focused intervention versus TAU**																
Green 2010	6.6	3.3	77	6.7	3.2	75	68.3%	−0.03 [−0.35, 0.29]	Low risk	+	+	−	+	+	+	+
Carter 2011	2.89	0.67	23	2.92	0.65	24	21.1%	−0.04 [−0.62, 0.53]	Low risk	+	?	−	+	−	?	?
Drew 2002	11	2.8	12	11.9	1.8	12	10.6%	−0.37 [−1.18, 0.44]	Low risk	+	?	−	?	+	?	?
**Subtotal (95% CI)**			**112**			**111**	**100.0%**	**−0.07 [−0.33, 0.19]**								
Heterogeneity: $Tau^2 = 0.00$; $Chi^2 = 0.59$, df = 2 (P = 0.74); $I^2 = 0\%$																
Test for overall effect: Z = 0.52 (P = 0.60)																
**3.1.3 Multimodal developmental intervention versus TAU**																
**Subtotal (95% CI)**			**0**			**0**		**Not estimable**								
Heterogeneity: Not applicable																
Test for overall effect: Not applicable																
**Total (95% CI)**			**112**			**111**	**100.0%**	**−0.07 [−0.33, 0.19]**								
Heterogeneity: $Tau^2 = 0.00$; $Chi^2 = 0.59$, df = 2 (P = 0.74); $I^2 = 0\%$																
Test for overall effect: Z = 0.52 (P = 0.60)																
Test for subgroup differences: Not applicable																

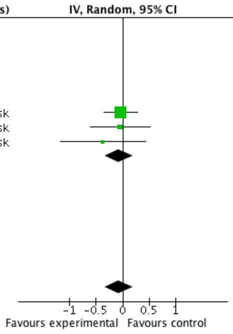

Risk of bias legend
(**A**) Random sequence generation (selection bias)
(**B**) Allocation concealment (selection bias)
(**C**) Blinding of participants and personnel (performance bias)
(**D**) Blinding of outcome assessment (detection bias)
(**E**) Incomplete outcome data (attrition bias)
(**F**) Selective reporting (reporting bias)
(**G**) Other bias

(Minus direction indicates positive effectiveness.)

3.3. Autism symptom: restricted repetitive and stereotyped patterns of behaviour, interests, and activities (Analysis II)

Study or Subgroup	Experimental Mean	Experimental SD	Experimental Total	Control Mean	Control SD	Control Total	Weight	Std. Mean Difference IV, Random, 95% CI	Risk of Bias A B C D E F G
**4.5.1 Behavioural intervention versus TAU**									
**Subtotal (95% CI)**			**0**			**0**		**Not estimable**	
Heterogeneity: Not applicable									
Test for overall effect: Not applicable									
**4.5.2 Social-communication focused intervention versus TAU**									
Drew 2002	3.9	1.8	12	4.2	2	12	7.6%	-0.15 [-0.95, 0.65]	+ ? - ? + ? ?
Green 2010	3	1.7	77	3.5	1.6	75	47.5%	-0.30 [-0.62, 0.02]	+ + - + + + +
**Subtotal (95% CI)**			**89**			**87**	**55.1%**	**-0.28 [-0.58, 0.02]**	
Heterogeneity: $Tau^2 = 0.00$; $Chi^2 = 0.11$, df = 1 (P = 0.74); $I^2 = 0\%$									
Test for overall effect: Z = 1.85 (P = 0.06)									
**4.5.3 Multimodal intervention versus TAU**									
Dawson 2010	16.7	13.1	24	22	16.3	21	13.9%	-0.35 [-0.95, 0.24]	+ ? - + ? + +
Rogers 2012	3.96	1.86	49	3.82	2.04	49	31.0%	0.07 [-0.32, 0.47]	+ + - ? ? + +
**Subtotal (95% CI)**			**73**			**70**	**44.9%**	**-0.08 [-0.48, 0.32]**	
Heterogeneity: $Tau^2 = 0.02$; $Chi^2 = 1.38$, df = 1 (P = 0.24); $I^2 = 27\%$									
Test for overall effect: Z = 0.41 (P = 0.68)									
**Total (95% CI)**			**162**			**157**	**100.0%**	**-0.18 [-0.40, 0.04]**	
Heterogeneity: $Tau^2 = 0.00$; $Chi^2 = 2.44$, df = 3 (P = 0.49); $I^2 = 0\%$									
Test for overall effect: Z = 1.62 (P = 0.11)									
Test for subgroup differences: $Chi^2 = 0.60$, df = 1 (P = 0.44), $I^2 = 0\%$									

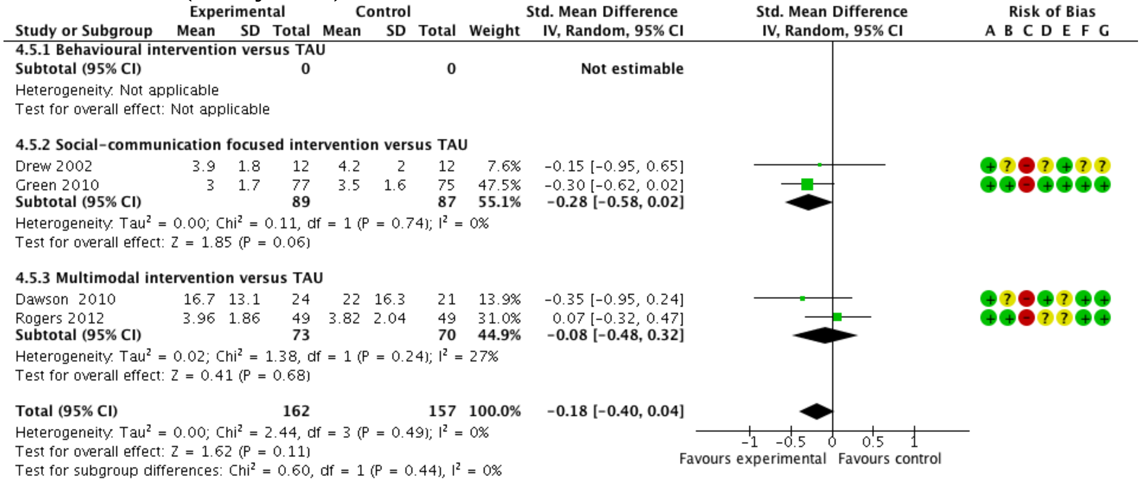

Risk of bias legend
(A) Random sequence generation (selection bias)
(B) Allocation concealment (selection bias)
(C) Blinding of participants and personnel (performance bias)
(D) Blinding of outcome assessment (detection bias)
(E) Incomplete outcome data (attrition bias)
(F) Selective reporting (reporting bias)
(G) Other bias

(Minus direction indicates positive effectiveness.)

3.4. Initiating joint attention (Analysis II)

Study or Subgroup	Experimental Mean	Experimental SD	Experimental Total	Control Mean	Control SD	Control Total	Weight	Std. Mean Difference IV, Random, 95% CI	Risk of Bias A B C D E F G
**8.1.1 Behavioural Intervention versus TAU**									
**Subtotal (95% CI)**			**0**			**0**		**Not estimable**	
Heterogeneity: Not applicable									
Test for overall effect: Not applicable									
**8.1.2 Social-communication focused intervention versus TAU**									
Carter 2011	10.33	9.82	24	8.68	9.26	25	12.2%	0.17 [-0.39, 0.73]	+ ? - + - ? ?
Goods 2013	0.4	0.89	5	1	1.73	6	3.9%	-0.39 [-1.59, 0.82]	+ ? - + - - ?
Ingersoll 2012	5.93	6.72	14	1.46	2.11	13	7.7%	0.86 [0.06, 1.65]	+ ? - ? ? ? ?
Kaale 2012	1.6	2.6	34	1.6	2.6	27	13.7%	0.00 [-0.51, 0.51]	+ + ? + + + ?
Kasari 2010	3.11	3.41	19	3.77	3.76	19	10.4%	-0.18 [-0.82, 0.46]	+ ? - + + + ?
Kim 2008	9	6.28	5	5.8	6.22	5	3.6%	0.46 [-0.80, 1.73]	? ? - ? ? + +
Landa 2011	8.83	13.22	24	4.42	4.82	24	11.9%	0.44 [-0.14, 1.01]	? ? - + + + ?
Lawton 2012	15.33	10.89	9	9	7.23	7	5.2%	0.63 [-0.39, 1.65]	+ + - + + ? +
Schertz 2013	3.27	3.17	11	0.86	0.77	12	6.6%	1.03 [0.15, 1.91]	? ? - ? ? ? +
**Subtotal (95% CI)**			**145**			**138**	**75.2%**	**0.29 [0.01, 0.56]**	
Heterogeneity: $Tau^2 = 0.04$; $Chi^2 = 10.11$, df = 8 (P = 0.26); $I^2 = 21\%$									
Test for overall effect: Z = 2.04 (P = 0.04)									
**8.1.3 Multimodal developmental intervention versus TAU**									
Casenhiser 2011	1.84	0.549	16	1.23	0.43	13	7.6%	1.19 [0.38, 1.99]	+ ? - + ? ? ?
Rogers 2012	4.58	3.45	49	3.76	3.44	49	17.2%	0.24 [-0.16, 0.63]	+ + - ? ? + +
**Subtotal (95% CI)**			**65**			**62**	**24.8%**	**0.64 [-0.28, 1.57]**	
Heterogeneity: $Tau^2 = 0.35$; $Chi^2 = 4.33$, df = 1 (P = 0.04); $I^2 = 77\%$									
Test for overall effect: Z = 1.37 (P = 0.17)									
**Total (95% CI)**			**210**			**200**	**100.0%**	**0.35 [0.10, 0.61]**	
Heterogeneity: $Tau^2 = 0.06$; $Chi^2 = 14.96$, df = 10 (P = 0.13); $I^2 = 33\%$									
Test for overall effect: Z = 2.69 (P = 0.007)									
Test for subgroup differences: $Chi^2 = 0.53$, df = 1 (P = 0.47), $I^2 = 0\%$									

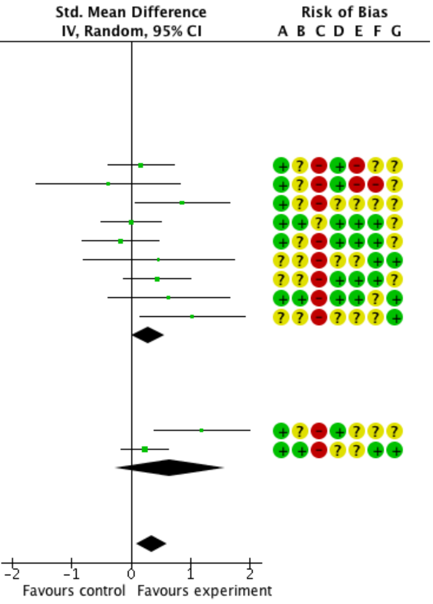

Risk of bias legend
(A) Random sequence generation (selection bias)
(B) Allocation concealment (selection bias)
(C) Blinding of participants and personnel (performance bias)
(D) Blinding of outcome assessment (detection bias)
(E) Incomplete outcome data (attrition bias)
(F) Selective reporting (reporting bias)
(G) Other bias

(Plus direction indicates positive effectiveness.)

3.5. Responding to joint attention (Analysis II)

Study or Subgroup	Experimental Mean	Experimental SD	Experimental Total	Control Mean	Control SD	Control Total	Weight	Mean Difference IV, Random, 95% CI	Random sequence generation (selection bias)
**8.6.1 Behavioural intervention versus TAU**									
Reitzel 2013	1.7	1.3	7	1.6	2.1	7	11.0%	0.10 [-1.73, 1.93]	Low risk
**Subtotal (95% CI)**			**7**			**7**	**11.0%**	**0.10 [-1.73, 1.93]**	
Heterogeneity: Not applicable									
Test for overall effect: Z = 0.11 (P = 0.91)									
**8.6.2 Social-communication focused intervention versus TAU**									
Kasari 2010	0.79	0.23	19	0.05	0.23	19	41.8%	0.74 [0.59, 0.89]	Low risk
Kim 2008	9.4	3.78	5	7.2	6.46	5	1.1%	2.20 [-4.36, 8.76]	Unclear risk
Schertz 2013	6.29	5.53	11	0.58	0.68	12	4.1%	5.71 [2.42, 9.00]	Unclear risk
**Subtotal (95% CI)**			**35**			**36**	**47.0%**	**2.72 [-1.02, 6.47]**	
Heterogeneity: $Tau^2 = 7.82$; $Chi^2 = 8.93$, df = 2 (P = 0.01); $I^2 = 78\%$									
Test for overall effect: Z = 1.43 (P = 0.15)									
**8.6.3 Multimodal developmental intervention versus TAU**									
Rogers 2012	0.34	0.29	49	0.34	0.34	49	42.0%	0.00 [-0.13, 0.13]	Low risk
**Subtotal (95% CI)**			**49**			**49**	**42.0%**	**0.00 [-0.13, 0.13]**	
Heterogeneity: Not applicable									
Test for overall effect: Z = 0.00 (P = 1.00)									
**Total (95% CI)**			**91**			**92**	**100.0%**	**0.58 [-0.12, 1.28]**	

Heterogeneity: $Tau^2 = 0.30$; $Chi^2 = 67.48$, df = 4 (P < 0.00001); $I^2 = 94\%$
Test for overall effect: Z = 1.62 (P = 0.11)
Test for subgroup differences: $Chi^2 = 2.04$, df = 2 (P = 0.36), $I^2 = 2.0\%$

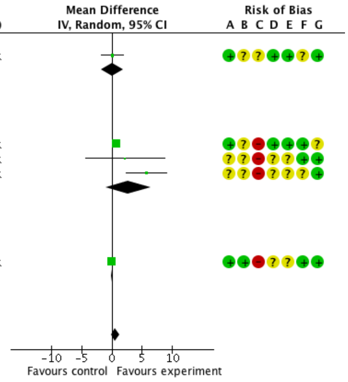

Risk of bias legend
(**A**) Random sequence generation (selection bias)
(**B**) Allocation concealment (selection bias)
(**C**) Blinding of participants and personnel (performance bias)
(**D**) Blinding of outcome assessment (detection bias)
(**E**) Incomplete outcome data (attrition bias)
(**F**) Selective reporting (reporting bias)
(**G**) Other bias

(Plus direction indicates positive effectiveness.)

3.6. Imitation (Analysis II)

Study or Subgroup	Experimental Mean	Experimental SD	Experimental Total	Control Mean	Control SD	Control Total	Weight	Std. Mean Difference IV, Random, 95% CI	Random sequence generation (selection bias)
**9.1.1 Behavioural intervention versus TAU**									
**Subtotal (95% CI)**			**0**			**0**		**Not estimable**	
Heterogeneity: Not applicable									
Test for overall effect: Not applicable									
**9.1.2 Social-communication focused intervention versus TAU**									
Ingersoll 2010	14.93	11.54	11	4.92	4.7	10	18.8%	1.07 [0.14, 2.00]	Low risk
Landa 2011	0.44	0.22	24	0.28	0.21	24	33.8%	0.73 [0.15, 1.32]	Unclear risk
**Subtotal (95% CI)**			**35**			**34**	**52.6%**	**0.83 [0.33, 1.32]**	
Heterogeneity: $Tau^2 = 0.00$; $Chi^2 = 0.37$, df = 1 (P = 0.55); $I^2 = 0\%$									
Test for overall effect: Z = 3.28 (P = 0.001)									
**9.1.3 Multimodal developmental intervention versus TAU**									
Rogers 2012	4.58	3.45	49	3.76	3.44	49	47.4%	0.24 [-0.16, 0.63]	Low risk
**Subtotal (95% CI)**			**49**			**49**	**47.4%**	**0.24 [-0.16, 0.63]**	
Heterogeneity: Not applicable									
Test for overall effect: Z = 1.16 (P = 0.24)									
**Total (95% CI)**			**84**			**83**	**100.0%**	**0.56 [0.09, 1.03]**	

Heterogeneity: $Tau^2 = 0.08$; $Chi^2 = 3.70$, df = 2 (P = 0.16); $I^2 = 46\%$
Test for overall effect: Z = 2.35 (P = 0.02)
Test for subgroup differences: $Chi^2 = 3.34$, df = 1 (P = 0.07), $I^2 = 70.0\%$

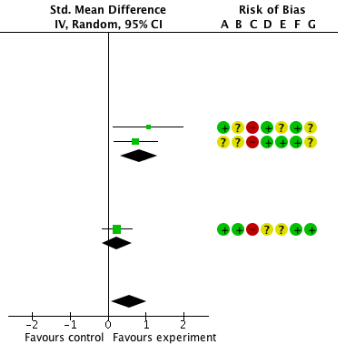

Risk of bias legend
(**A**) Random sequence generation (selection bias)
(**B**) Allocation concealment (selection bias)
(**C**) Blinding of participants and personnel (performance bias)
(**D**) Blinding of outcome assessment (detection bias)
(**E**) Incomplete outcome data (attrition bias)
(**F**) Selective reporting (reporting bias)
(**G**) Other bias

(Plus direction indicates positive effectiveness.)

3.7. Symbolic play (Analysis II)

Study or Subgroup	Experimental Mean	Experimental SD	Experimental Total	Control Mean	Control SD	Control Total	Weight	Std. Mean Difference IV, Random, 95% CI	Random sequence generation (selection bias)
**13.1.1 Behavioural intervention versus TAU**									
**Subtotal (95% CI)**			**0**			**0**		**Not estimable**	
Heterogeneity: Not applicable									
Test for overall effect: Not applicable									
**13.1.2 Social-communication focused intervention versus TAU**									
Goods 2013	22	10.17	5	14.33	9.69	6	33.5%	0.71 [-0.54, 1.95]	Low risk
Kasari 2010	0.26	0.65	19	0.53	1.43	19	66.5%	-0.24 [-0.88, 0.40]	Low risk
**Subtotal (95% CI)**			**24**			**25**	**100.0%**	**0.08 [-0.80, 0.95]**	
Heterogeneity: $Tau^2 = 0.19$; $Chi^2 = 1.76$, df = 1 (P = 0.18); $I^2 = 43\%$									
Test for overall effect: Z = 0.18 (P = 0.86)									
**13.1.3 Multimodal interventions versus TAU**									
**Subtotal (95% CI)**			**0**			**0**		**Not estimable**	
Heterogeneity: Not applicable									
Test for overall effect: Not applicable									
**Total (95% CI)**			**24**			**25**	**100.0%**	**0.08 [-0.80, 0.95]**	
Heterogeneity: $Tau^2 = 0.19$; $Chi^2 = 1.76$, df = 1 (P = 0.18); $I^2 = 43\%$									
Test for overall effect: Z = 0.18 (P = 0.86)									
Test for subgroup differences: Not applicable									

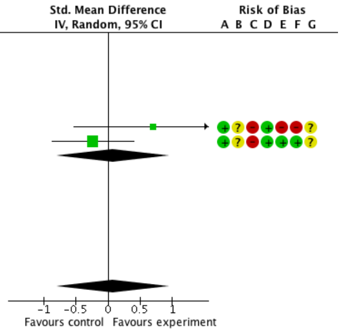

Risk of bias legend
(**A**) Random sequence generation (selection bias)
(**B**) Allocation concealment (selection bias)
(**C**) Blinding of participants and personnel (performance bias)
(**D**) Blinding of outcome assessment (detection bias)
(**E**) Incomplete outcome data (attrition bias)
(**F**) Selective reporting (reporting bias)
(**G**) Other bias

(Plus direction indicates positive effectiveness.)

3.8. Functional play (Analysis II)

Study or Subgroup	Experimental Mean	Experimental SD	Experimental Total	Control Mean	Control SD	Control Total	Weight	Std. Mean Difference IV, Random, 95% CI	Random sequence generation (selection bias)
**13.2.1 Behavioural intervention versus TAU**									
**Subtotal (95% CI)**			**0**			**0**		**Not estimable**	
Heterogeneity: Not applicable									
Test for overall effect: Not applicable									
**13.2.2 Social-communication focused intervention versus TAU**									
Goods 2013	22	10.17	5	14.33	9.69	6	22.3%	0.71 [-0.54, 1.95]	Low risk
Kasari 2010	5.29	2.37	19	3.29	2.3	19	77.7%	0.84 [0.17, 1.50]	Low risk
**Subtotal (95% CI)**			**24**			**25**	**100.0%**	**0.81 [0.22, 1.40]**	
Heterogeneity: $Tau^2 = 0.00$; $Chi^2 = 0.03$, df = 1 (P = 0.86); $I^2 = 0\%$									
Test for overall effect: Z = 2.70 (P = 0.007)									
**13.2.3 Multimodal intervention versus TAU**									
**Subtotal (95% CI)**			**0**			**0**		**Not estimable**	
Heterogeneity: Not applicable									
Test for overall effect: Not applicable									
**Total (95% CI)**			**24**			**25**	**100.0%**	**0.81 [0.22, 1.40]**	
Heterogeneity: $Tau^2 = 0.00$; $Chi^2 = 0.03$, df = 1 (P = 0.86); $I^2 = 0\%$									
Test for overall effect: Z = 2.70 (P = 0.007)									
Test for subgroup differences: Not applicable									

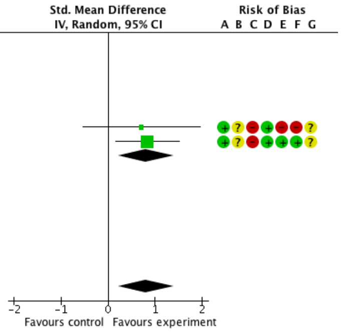

Risk of bias legend
(**A**) Random sequence generation (selection bias)
(**B**) Allocation concealment (selection bias)
(**C**) Blinding of participants and personnel (performance bias)
(**D**) Blinding of outcome assessment (detection bias)
(**E**) Incomplete outcome data (attrition bias)
(**F**) Selective reporting (reporting bias)
(**G**) Other bias

(Plus direction indicates positive effectiveness.)

3.9. Parental synchrony (Analysis II)

Study or Subgroup	Experimental Mean	Experimental SD	Experimental Total	Control Mean	Control SD	Control Total	Weight	Std. Mean Difference IV, Random, 95% CI	Random sequence generation (selection bias)
**16.4.1 Behavioural intervention versus TAU**									
**Subtotal (95% CI)**			**0**			**0**		**Not estimable**	
Heterogeneity: Not applicable									
Test for overall effect: Not applicable									
**16.4.2 Social-communication focused intervention versus TAU**									
Carter 2011	0.34	0.07	23	0.3	0.1	24	18.0%	0.45 [−0.13, 1.03]	Low risk
Green 2010	51.3	19.6	77	32.6	14	75	19.7%	1.09 [0.75, 1.43]	Low risk
Aldred 2004	65.1	14.3	14	49.5	18.9	14	16.2%	0.90 [0.12, 1.69]	Low risk
Thompson 2012	201.6	24.82	10	191.33	21.93	9	15.1%	0.42 [−0.50, 1.33]	Low risk
Venker 2012	74.57	33.51	7	73	33.51	7	13.9%	0.04 [−1.00, 1.09]	Unclear risk
Siller 2013	0.72	0.04	34	0.61	0.04	30	17.1%	2.72 [2.03, 3.41]	Unclear risk
**Subtotal (95% CI)**			**165**			**159**	**100.0%**	**0.98 [0.30, 1.66]**	
Heterogeneity: $Tau^2 = 0.58; Chi^2 = 31.99, df = 5 (P < 0.00001); I^2 = 84\%$									
Test for overall effect: $Z = 2.82 (P = 0.005)$									
**16.4.3 Multimodal developmental intervention versus TAU**									
**Subtotal (95% CI)**			**0**			**0**		**Not estimable**	
Heterogeneity: Not applicable									
Test for overall effect: Not applicable									
**Total (95% CI)**			**165**			**159**	**100.0%**	**0.98 [0.30, 1.66]**	

Heterogeneity: $Tau^2 = 0.58; Chi^2 = 31.99, df = 5 (P < 0.00001); I^2 = 84\%$
Test for overall effect: $Z = 2.82 (P = 0.005)$
Test for subgroup differences: Not applicable

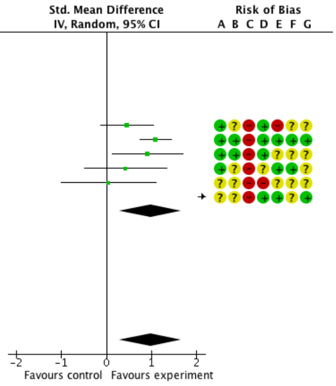

Risk of bias legend
(**A**) Random sequence generation (selection bias)
(**B**) Allocation concealment (selection bias)
(**C**) Blinding of participants and personnel (performance bias)
(**D**) Blinding of outcome assessment (detection bias)
(**E**) Incomplete outcome data (attrition bias)
(**F**) Selective reporting (reporting bias)
(**G**) Other bias

(Plus direction indicates positive effectiveness.)

3.10. Parenting stress (Analysis II)

Study or Subgroup	Experimental Mean	Experimental SD	Experimental Total	Control Mean	Control SD	Control Total	Weight	Std. Mean Difference IV, Random, 95% CI	Random sequence generation (selection bias)
**15.1.1 Behavioural intervention versus TAU**									
**Subtotal (95% CI)**			**0**			**0**		**Not estimable**	
Heterogeneity: Not applicable									
Test for overall effect: Not applicable									
**15.1.2 Social-communication focused intervention versus TAU**									
Drew 2002	104.3	20	10	112.1	20.1	10	12.8%	−0.37 [−1.26, 0.51]	Low risk
**Subtotal (95% CI)**			**10**			**10**	**12.8%**	**−0.37 [−1.26, 0.51]**	
Heterogeneity: Not applicable									
Test for overall effect: $Z = 0.82 (P = 0.41)$									
**15.1.3 Multimodal developmental intervention versus TAU**									
Roberts 2011 Home-based	92.7	20.9	20	90.4	22.9	11	18.5%	0.10 [−0.63, 0.84]	Low risk
Ichikawa 2013	213	34.8	5	204.3	36.3	6	7.1%	0.22 [−0.97, 1.42]	Low risk
Roberts 2011 Centre-based	98.2	20.1	20	90.4	22.9	11	18.2%	0.36 [−0.38, 1.10]	Low risk
Welterlin 2012	242.4	41.5	10	256.2	70	10	13.0%	−0.23 [−1.11, 0.65]	Unclear risk
Tonge 2006	1.86	0.88	35	2.26	1.09	18	30.4%	−0.41 [−0.99, 0.16]	Unclear risk
**Subtotal (95% CI)**			**90**			**56**	**87.2%**	**−0.06 [−0.40, 0.28]**	
Heterogeneity: $Tau^2 = 0.00; Chi^2 = 3.23, df = 4 (P = 0.52); I^2 = 0\%$									
Test for overall effect: $Z = 0.36 (P = 0.72)$									
**Total (95% CI)**			**100**			**66**	**100.0%**	**−0.10 [−0.42, 0.21]**	

Heterogeneity: $Tau^2 = 0.00; Chi^2 = 3.64, df = 5 (P = 0.60); I^2 = 0\%$
Test for overall effect: $Z = 0.63 (P = 0.53)$
Test for subgroup differences: $Chi^2 = 0.41, df = 1 (P = 0.52), I^2 = 0\%$

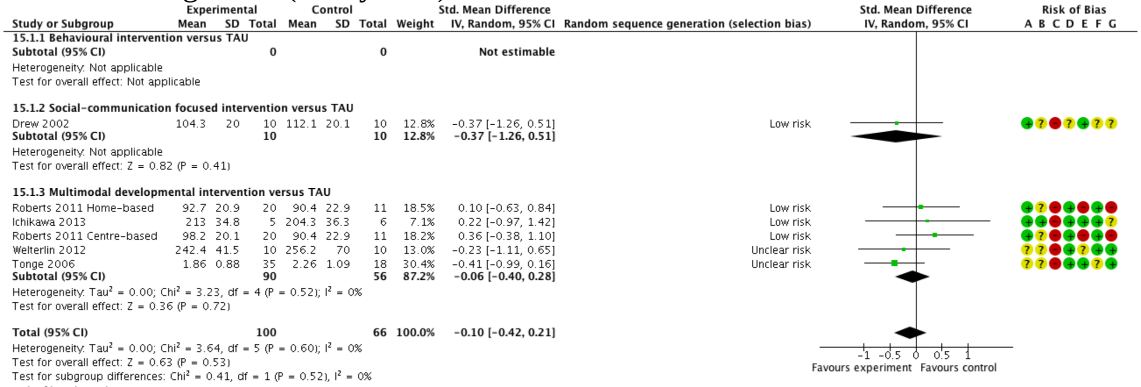

Risk of bias legend
(**A**) Random sequence generation (selection bias)
(**B**) Allocation concealment (selection bias)
(**C**) Blinding of participants and personnel (performance bias)
(**D**) Blinding of outcome assessment (detection bias)
(**E**) Incomplete outcome data (attrition bias)
(**F**) Selective reporting (reporting bias)
(**G**) Other bias

(Minus direction indicates positive effectiveness.)
