# Supplementary material for: A systematic review and meta-analysis of comprehensive interventions for pre-school children with autism spectrum disorder (ASD)
Source: PLoS One. 2017 Dec 6;12(12):e0186502. doi: 10.1371/journal.pone.0186502 (PMC5718481; doi:10.1371/journal.pone.0186502)
Supplement: S1 Table — (PDF) [file pone.0186502.s003.pdf]

S1 Table. Excluded studies

No.	Study	Reason for exclusion
1	Aldred, C., et al. (2012). "Brief report: mediation of treatment effect in a communication intervention for pre-school children with autism." Journal of Autism & Developmental Disorders	Subanalysis of the previous study
2	Aliee, Z. S., et al. (2013). "The effectiveness of managing split attention among autistic children using computer based intervention." TOJET: The Turkish Online Journal of Educational	Age 5-18
3	Andrews, L., et al. (2013). "Increasing the appropriate demonstration of affectionate behavior, in children with Asperger syndrome, high functioning autism, and PDD-NOS: A randomized controlled trial." Research in Autism Spectrum Disorders **7**(12):	Age 7-12
4	Asghari Nekah, S. M., et al. (2013). "The effect of puppet play therapy intervention on the communicative skills of autistic children." Boutros, Nash [Ed] **2**(pp. 199-215): 199-215.	Quasi-experimental - not RCT
5	Begeer, S., et al. (2011). "Theory of Mind training in children with autism: a randomized controlled trial." Journal of Autism & Developmental Disorders **41**(8): 997-1006.	Age 8-13
6	Berquist, K. L. and M. H. Charlop (2014). "Teaching parents of children with autism to evaluate interventions." Journal of Developmental and Physical Disabilities **26**(4): 451-472.	The focus was to evaluate parents skills.
7	Boyd, B. A., et al. (2013). "Feasibility of exposure response prevention to treat repetitive behaviors of children with autism and an intellectual disability: a brief report." Autism **17**(2): 196-204.	Quasi-experimental - not RCT
8	Bressel, E., et al. (2011). "Effect of whole body vibration on stereotypy of young children with autism." BMJ Case Reports.	Not RCT, pre/post with 3 children
9	Cardon, T. A. and M. Wilcox (2011). "Promoting imitation in young children with autism: A comparison of reciprocal imitation training and video modeling." Journal of Autism and	Compare two experimental groups
10	Causin, K. G., et al. (2013). "The role of joint control in teaching listener responding to children with autism and other developmental disabilities." Research in Autism Spectrum	multiple probe design
11	Dawson, G., et al. (2012). "Early behavioral intervention is associated with normalized brain activity in young children with autism." Journal of the American Academy of Child & Adolescent	Outcome is EEG - behavioral outcome was previously published in
12	D'Elia, L., et al. (2014). "A longitudinal study of the TEACCH program in different settings: The potential benefits of low intensity intervention in preschool children with autism spectrum disorder." Journal of Autism and Developmental Disorders **44**(3):	Not randomized
13	DeRosier, M. E., et al. (2011). "The efficacy of a Social Skills Group Intervention for improving social behaviors in children with High Functioning Autism Spectrum disorders." Journal of Autism & Developmental Disorders **41**(8): 1033-1043.	Age 8-12
14	Dezfoolian, L., et al. (2013). "A pilot study on the effects of Orff-based therapeutic music in children with autism spectrum disorder." Music and Medicine **5**(3): 162-168.	No control group
15	Eapen, V., et al. (2013). "Clinical outcomes of an early intervention program for preschool children with Autism Spectrum Disorder in a community group setting." BMC Pediatrics **13**(1): 3.	Pre-post comparison (No control group)
16	Eldevik, S., et al. (2012). "Outcomes of Behavioral Intervention for Children with Autism in Mainstream Pre-School Settings."	Not RCT

17	Farmer, C., et al. (2012). "Predictors and moderators of parent training efficacy in a sample of children with autism spectrum disorders and serious behavioral problems." Journal of Autism & Developmental Disorders **42**(6): 1037-1044.	Not RCT, age 4-14
18	Fernell, E., et al. (2011). "Early intervention in 208 Swedish preschoolers with autism spectrum disorder. A prospective naturalistic study." Research in Developmental Disabilities **32**(6):	Prospective naturalistic study
19	Field, T., et al. (2013). "Reciprocal imitation following adult imitation by children with autism." Infant and Child Development	Not TAU
20	Flanagan, H. E., et al. (2012). "Effectiveness of large-scale community-based intensive Behavioral Intervention: A waitlist comparison study exploring outcomes and predictors." Research in Autism Spectrum Disorders **6**(2): 673-682.	Not RCT
21	Foussier, S., et al. (2012). "Behaviour regulation, stimulation and homogenisation of social/emotional and cognitive development in children with autism: A recreational approach by 2-player-game (pun in French between first person pronoun "I" and the word "game" which sound the same)." A N A E Approche Neuropsychologique des Apprentissages chez l'Enfant **24**(4[119]:	French article<br>Not RCT
22	Fragala-Pinkham, M. A., et al. (2011). "Group swimming and aquatic exercise programme for children with autism spectrum disorders: a pilot study." Developmental neurorehabilitation **14**(4):	group swimming and aquatic exercise
23	Freitag, C. M., et al. (2012). "The Frankfurt early intervention program FFIP for preschool aged children with autism spectrum disorder: a pilot study." Journal of Neural Transmission **119**(9):	No control
24	Fujii, C., et al. (2013). "Intensive cognitive behavioral therapy for anxiety disorders in school-aged children with autism: A preliminary comparison with treatment-as-usual." School Mental	Age 7-11
25	Ganz, J. B., et al. (2013). "Effectiveness of the PECS Phase III app and choice between the app and traditional PECS among preschoolers with ASD." Research in Autism Spectrum Disorders	Single case multiple baseline
26	Gatta, M., et al. (2013). "Novelty learning in pervasive developmental disorder children: A comparison between psychomotor therapy and psychoeducational intervention." Giornale di Neuropsichiatria dell'Eta Evolutiva **33**(2): 75-86.	Two comparative study, 6 children)
27	Goh, S., et al. (2013). "Teaching non-verbal children with autistic disorder to read and write: A pilot study." International Journal of Developmental Disabilities **59**(2): 95-107.	Age 5-13
28	Gordon, K., et al. (2011). "A communication-based intervention for nonverbal children with autism: what changes? Who benefits?" Journal of Consulting & Clinical Psychology **79**(4): 447-457.	Age 4-10
29	Green, J., et al. (2013). "Intervention for infants at risk of developing autism: A case series." Journal of Autism and Developmental Disorders **43**(11): 2502-2514.	Case series
30	Gulsrud, A. C., et al. (2014). "Two to ten years: developmental trajectories of joint attention in children with ASD who received targeted social communication interventions." Autism research : Official Journal of the International Society for Autism Research	Growth model analysis of data from previous RCT
31	Hastings, R. P., et al. (2012). "Interventions for children with pervasive developmental disorders in low and middle income countries." Journal of Applied Research in Intellectual Disabilities	No random assignment

32	Hopkins, I. M., et al. (2011) Avatar assistant: Improving social skills in students with an asd through a computer-based intervention. Journal of Autism and Developmental Disorders **41**, 1543-1555 DOI: http://dx.doi.org/10.1007/s10803-011-1179-z	Age 6-15
33	Huskens, B., et al. (2013). "Promoting question-asking in school-aged children with autism spectrum disorders: effectiveness of a robot intervention compared to a human-trainer intervention." Developmental neurorehabilitation **16**(5): 345-356.	School age
34	Ingersoll, B. R. and A. L. Wainer (2013). "Pilot study of a school-based parent training program for preschoolers with ASD." Autism	Not RCT
35	Jang, J., et al. (2012). "Randomized trial of an eLearning program for training family members of children with autism in the principles and procedures of applied behavior analysis." Research in Autism Spectrum Disorders **6**(2): 852-856.	Age 3.1-11.5, not children's behavior outcome
36	Kasari, C. (2014). "Are we there yet? The state of early prediction and intervention in autism spectrum disorder." Journal of the American Academy of Child & Adolescent Psychiatry **53**(2): 133-134.	Sequential multiple assignment randomized trial, age 5-8, speech utterance is main outcome
37	Kasari, C., et al. (2014). "Caregiver-mediated intervention for low-resourced preschoolers with autism: an RCT." Pediatrics **134**(1):	Control group was not "treatment as usual".
38	Kasari, C., et al. (2012). "Longitudinal follow-up of children with autism receiving targeted interventions on joint attention and play." Journal of the American Academy of Child & Adolescent	Age 6-11
39	Kasari, C., et al. (2012). "Making the connection: randomized controlled trial of social skills at school for children with autism spectrum disorders." Journal of Child Psychology & Psychiatry &	School age (Mean age 8.14 years)
40	Kasari, C., et al. (2006). "Joint attention and symbolic play in young children with autism: a randomized controlled intervention study." Journal of Child Psychology and Psychiatry **47**(6): 611-	Not TAU
41	Kasari, C., et al. (2008). "Language outcome in autism: randomized comparison of joint attention and play interventions." Journal of Consulting & Clinical Psychology **76**(1): 125-137.	Not TAU
42	Kassardjian, A., et al. (2013). "Utilizing teaching interactions to facilitate social skills in the natural environment." Education and Training in Autism and Developmental Disabilities **48**(2): 245-	4 kids
43	Kenworthy, L., et al. (2014). "Randomized controlled effectiveness trial of executive function intervention for children on the autism spectrum." Journal of Child Psychology &	age 3-5th graders
44	Kern, J. K., et al. (2011). "Prospective trial of equine-assisted activities in autism spectrum disorder." Alternative Therapies in Health & Medicine **17**(3): 14-20.	No random assignment, age 3-12
45	Kern, J. K., et al. (2011). "A clinical trial of glutathione supplementation in autism spectrum disorders." Medical Science	Nutrition supplement study
46	Kovshoff, H., et al. (2011). "Two-year outcomes for children with autism after the cessation of early intensive behavioral intervention." Behavior Modification **35**(5): 427-450.	Not RCT
47	Kretzmann, M. (2013). "Facilitating peer engagement between children with autism and their classmates at school." Dissertation Abstracts International Section A: Humanities and Social Sciences **74**(3-A(E)): No Pagination Specified.	Elementary school kids

48	Lawton, K. and C. Kasari (2012). "Brief report: longitudinal improvements in the quality of joint attention in preschool children with autism." Journal of Autism & Developmental Disorders	Not TAU
49	Lee, R. and P. Sturmey (2014). "The effects of script-fading and a Lag-1 schedule on varied social responding in children with autism." Research in Autism Spectrum Disorders **8**(4): 440-448.	Multiple baseline across participants
50	Lepper, T. L., et al. (2013). "Effects of operant discrimination training on the vocalizations of nonverbal children with autism." Journal of Applied Behavior Analysis **46**(3): 656-661.	not RCT, 3 boys
51	Lim, H. A. and E. Draper (2011). "The effects of music therapy incorporated with applied behavior analysis verbal behavior approach for children with autism spectrum disorders." Journal of	The intervention period was too short (3 days).
52	Lim, H. (2010). "Effect of "Developmental Speech and Language Training Through Music" on Speech Procuction in Children with Autism Spectrum Disorders." Journal of Music Therapy 47(1):2-	The intervention period was too short (3 days).
53	Magiati, I., et al. (2011). "Patterns of Change in Children with Autism Spectrum Disorders Who Received Community Based Comprehensive Interventions in Their Pre-School Years: A Seven Year Follow-Up Study." **5**(3): 1016-1027.	Not RCT, naturalistic
54	Mandelberg, J., et al. (2014). "Long-term outcomes of parent-assisted social skills intervention for high-functioning children with autism spectrum disorders." Autism **18**(3): 255-263.	Age is 12.6 after 3 years
55	Mandell, D. S. (2013). "Adults with autism-A new minority." Journal of General Internal Medicine **28**(6): 751-752.	Age 5-8, kinder to 2nd grade
56	Matson, J. L. and R. L. Goldin (2014). "Early Intensive Behavioral Interventions: Selecting behaviors for treatment and assessing treatment effectiveness." Research in Autism Spectrum Disorders	Turned out to be a review article
57	McDuffie, A., et al. (2010). "Autism spectrum disorder in children and adolescents with fragile X syndrome: Within-syndrome differences and age-related changes." American Journal on Intellectual and Developmental Disabilities **115**(4): 307-326.	Compared two intervention programs
58	Minne, E. P. and M. Semrud-Clikeman (2012). "A Social Competence Intervention for Young Children with High Functioning Autism and Asperger Syndrome: A Pilot Study."	Age 6-7, 5 children, not RCT
59	Mitchell, E. S. (2013). "The effectiveness of a behavioral summer treatment program for children with high functioning autism spectrum disorder." Dissertation Abstracts International: Section B: The Sciences and Engineering **74**(1-B(E)): No Pagination	No control group
60	Mohammadzaheri, F., et al. (2014). "A randomized clinical trial comparison between pivotal response treatment (prt) and structured applied behavior analysis (aba) intervention for children with autism." Journal of Autism and Developmental Disorders	Compared 2 ABA programs with a structured ABA
61	Murdock, L. C. and J. Q. Hobbs (2011). "Picture me playing: increasing pretend play dialogue of children with autism spectrum disorders." Journal of Autism & Developmental Disorders **41**(7):	Single treatment counterbalanced design
62	Murdock, L. C., et al. (2014). "The effect of a platform swing on the independent work behaviors of children with Autism Spectrum Disorders." Focus on Autism and Other Developmental	Platform swing - not an active intervention

63	Nefdt, N., Koegel, R., Singer, G. and Gerber, M. (2010). "The Use of a Self-Directed Learning Program to Provide Introductory Training in Pivotal Response Treatment to Parents of Children With Autism Treatment to Parents of Children With Autism" Journal of Positive Behavior Interventions 2010; 12; 23	Intervention duration was too short (7 days).
64	Okuno, H., et al. (2013). "[Effectiveness of a modified parent training of smaller groups and shorter schedules for children with pervasive developmental disorders]." No to Hattatsu [Brain & Development] **45**(1): 26-32.	Pre-post comparison, included ADHD
65	Oosterling, I., et al. (2010) Randomized controlled trial of the focus parent training for toddlers with autism: 1-year outcome. Journal of Autism and Developmental Disorders **40**, 1447-1458 DOI: 10.1007/s10803-010-1004-0	Not RCT
66	Park, M. N. (2013). "Targeting social communication impairments in children with autism spectrum disorders through self-management." Dissertation Abstracts International: Section B: The Sciences and Engineering **74**(4-B(E)): No Pagination Specified.	not RCT
67	Perry, T. L. (2013). "The effects of response interruption and redirection on language skills in children with vocal stereotypy." Dissertation Abstracts International: Section B: The Sciences and Engineering **74**(4-B(E)): No Pagination Specified.	Not RCT
68	Persicke, A., et al. (2013). "Teaching children with autism to attend to socially relevant stimuli." Research in Autism Spectrum Disorders **7**(12): 1551-1557.	Not RCT - 3 participants
69	Peters-Scheffer, N., et al. (2013). "Therapist characteristics predict discrete trial teaching procedural fidelity." Intellectual & Developmental Disabilities **51**(4): 263-272.	Sample also includes intellectual disability, not just ASD
70	Pfeiffer, B. A., et al. (2011). "Effectiveness of sensory integration interventions in children with autism spectrum disorders: a pilot study." American Journal of Occupational Therapy **65**(1): 76-85.	Sensory integration
71	Radley, K. C., et al. (2014). "The feasibility and effects of a parent-facilitated social skills training program on social engagement of children with autism spectrum disorders."	Not RCT, 5 kids
72	Reaven, J., et al. (2012). "Group cognitive behavior therapy for children with high-functioning autism spectrum disorders and anxiety: a randomized trial." Journal of Child Psychology & Psychiatry & Allied Disciplines **53**(4): 410-419.	Age 7-14
73	Reichow, B. and M. Wolery (2011). "Comparison of progressive prompt delay with and without instructive feedback." Journal of Applied Behavior Analysis **44**(2): 327-340.	Not RCT, adapted alternating treatment design
74	Rickards, A., et al. (2009). "One-year follow-up of the outcome of a randomized controlled trial of a home-based intervention programme for children with autism and developmental delay and their families." Child: Care, Health and Development **35**(5): 593-	Children with developmental delay were included.
75	Roberts, J., et al. (2011). "A randomised controlled trial of two early intervention programs for young children with autism: Centre-based with parent program and home-based." Research in Autism Spectrum Disorders **5**(4): 1553-1566.	Not RCT

76	Romero, N. L. (2014). "Evaluating the effectiveness of a computer based intervention, the transporters, on both recognition and understanding of emotions in young children with autism." Dissertation Abstracts International Section A: Humanities and Social Sciences **74**(9-A(E)): No Pagination Specified.	Not RCT, 3 kids
77	Roux, G., et al. (2013). "A randomized controlled trial of group Stepping Stones Triple P: a mixed-disability trial." Family Process **52**(3): 411-424.	RCT but included children with Down syndrome and cerebral palsy.
78	Ruble, L. and J. H. McGrew (2013). "Teacher and child predictors of achieving IEP goals of children with autism." Journal of Autism & Developmental Disorders **43**(12): 2748-2763.	Age 3-9
79	Ruble, L. A., et al. (2013). "A randomized controlled trial of COMPASS web-based and face-to-face teacher coaching in autism." Journal of Consulting & Clinical Psychology **81**(3): 566-	School age children
80	Sallows, G. O. and T. D. Graupner (2005). "Intensive behavioral treatment for children with autism: four-year outcome and predictors." American Journal of Mental Retardation **110**(6): 417-	Not TAU
81	Samadi, S. A. and A. Mahmoodizadeh (2014). "Omid early intervention resource kit for children with autism spectrum disorders and their families." Early Child Development and Care	Focus was parents needs
82	Sandiford, G. A. (2013). "The efficacy of melodic based communication therapy for eliciting speech in nonverbal children with autism." Dissertation Abstracts International: Section B: The Sciences and Engineering **73**(11-B(E)): No Pagination Specified.	Outcome was solely language (e.g., number of correct words)
83	Scarpa, A. and N. M. Reyes (2011). "Improving emotion regulation with CBT in young children with high functioning autism spectrum disorders: a pilot study." Behavioural &	Emotion regulation was the main outcome, age 5-7
84	Schaaf, R. C., et al. (2014). "An intervention for sensory difficulties in children with autism: a randomized trial." Journal of Autism & Developmental Disorders **44**(7): 1493-1506.	Age 4-7:11
85	Shin, S., et al. (2012). "A Comparative Study of the Preliminary Effects in the Levels of Adaptive Behaviors: Learning Program for the Development of Children with Autism (LPDCA)." **13**(1): 6-15	Age preschool to high school
86	Schultz, T. R. (2013). "Evaluating the effectiveness of the core content of The Incredible Years with and without visual performance feedback for parents of children with autism." Dissertation Abstracts International Section A: Humanities and	Multiple baseline design
87	Schwartzberg, E. T. and M. J. Silverman (2013). "Effects of music-based social stories on comprehension and generalization of social skills in children with autism spectrum disorders: A randomized effectiveness study." The Arts in Psychotherapy **40**(3):	Age 9-21
88	Senechal, C., et al. (2013). "Parents as co-therapists: A winning solution for treating autistic children." Annales Medico-	Quasi-experimental - not RCT
89	Silva, L. M., et al. (2011). "Early intervention for autism with a parent-delivered Qigong massage program: a randomized controlled trial." American Journal of Occupational Therapy **65**(5):	Qigong massage by parents
90	Simpson, K., et al. (2013). "The use of music to engage children with autism in a receptive labelling task." Research in Autism Spectrum Disorders **7**(12): 1489-1496.	Not an active intervention to children (sung vs spoken condition)

91	Stock, R., et al. (2013). "Comparison of community-based verbal behavior and pivotal response treatment programs for young children with autism spectrum disorder." Research in Autism Spectrum Disorders **7**(9): 1168-1181.	Two comparative study
92	Strasberger, S. K. and S. J. Ferreri (2013). "The effects of peer assisted communication application training on the communicative and social behaviors of children with autism." Journal of Developmental and Physical Disabilities Oct(Pagination): No	Not RCT, 4 kids
93	Weiner, R. H. and R. L. Greene (2014). "Intention-based therapy for autism spectrum disorder: promising results of a wait-list control study in children." Explore: The Journal of Science &	Not RCT
94	Whyte, E. M., et al. (2013). "Learning of idiomatic language expressions in a group intervention for children with autism."	Age 7-12
95	Williams, B. T., et al. (2012). "Teaching emotion recognition skills to young children with autism: a randomised controlled trial of an emotion training programme." Journal of Child Psychology & Psychiatry & Allied Disciplines **53**(12): 1268-1276.	The intervention (watching DVD) was not classified into the three model.
96	Young, K. L., et al. (2012). "Evaluation of a Self-Instructional Package on Discrete-Trials Teaching to Parents of Children with Autism." **6**(4): 1321-1330.	Not RCT

RCT means randomized controlled trial. TAU means treatment as usual.
