# Supplementary material for: A systematic review and meta-analysis of comprehensive interventions for pre-school children with autism spectrum disorder (ASD)
Source: PLoS One. 2017 Dec 6;12(12):e0186502. doi: 10.1371/journal.pone.0186502 (PMC5718481; doi:10.1371/journal.pone.0186502)
Supplement: S3 Table — (PDF) [file pone.0186502.s005.pdf]

S3 Table. Outcome measure list used in the included studies for the data syntheses

			Pre (T1)		Pre (T1)		post (T2)			Post (T2)		
			Experiment		Control		Experiment			Control		
	Outcome	Measures	post-treatment outcome		Post-treatment outcome		post-treatment outcome			Post-treatment outcome		
			Mean	SD	Mean	SD	Mean	SD	n	Mean	SD	n
Aldred et al. (2004)	Autism symptoms: general	ADOS, total	16.1	4.5	15.6	4.9	11.8	6.4	14	16.1	4.4	14
	Expressive language	MCDI, expressive language	28	467	25.6	683	199.4	25606	14	33.1	683	14
	Receptive language	MCDI, language comprehension	71.7	2.383	95.4	426	222.7	40431	14	146.8	11426	14
	Maternal synchronization	Parent synchrony	57.8	15	57.1	49.5	65.1	14.3	14	49.5	18.9	14
	Joint engagement	Child communication acts	30.8	10.2	30.1	11.1	37.6	10.1	14	27.6	16.5	14
Carter et al. (2011)	Receptive language	MSEL, receptive Language	8.41	5.42	8.17	4.44	15.52	6.93	25	17.48	8.33	25
	Expressive language	MSEL, expressive Language	8.22	6.01	7.33	3.71	16.2	7.23	25	16.68	7.88	25
	Developmental quotient	MSEL, composite score					63.88	18.41	25	64.88	13.94	25
	Initiating joint attention	ESCS, frequency of initiating joint attention	5.9	5.41	5.59	6.14	10.33	9.82	24	8.68	9.26	25
	Maternal synchronization	PCFP, proportion of codable intervals with parental responsivity	0.32	0.06	0.29	0.08	0.34	0.07	23	0.3	0.1	24
Casenhiser et al. (2011)	Initiating joint attention	Modified CBRS, initiation of joint attention	1.28	0.542	1.31	0.987	1.84	0.549	16 (25-9)	1.23	0.43	13 (26-13)
Dawson et al. (2010) baseline imbalance	Developmental quotient	MSEL, early learning composite	76.4	23.4	64	13.8	78.6	24.2	24	66.3	15.3	21
	Receptive language	MSEL, receptive language	38.9	15.4	31.1	11.1	40	16.3	24	31.5	10.6	21
	Expressive language	MSEL, expressive language	36.1	14.2	33	11.5	36.6	13.6	24	30	9.2	21
	Adaptive behavior	VABS, composite	65.7	9.8	63.7	8.8	68.7	15.9	24	59.1	8.8	21
	Autism symptoms: general	ADOS, severity score	6.5	1.5	7.3	2.1	7	1.9	24	7.3	1.8	21
	Autism symptoms: restricted repetitive and stereotyped patterns of behavior, interests, and activities	RBS	15.5	12.3	23.3	17.5	16.7	13.1	24	22	16.3	21
Drew et al. (2002)	Developmental quotient	Non-verbal IQ from D and E scales of GSMD	88.1	11.2	66	16.5	77.9	14.8	12	66.1	17.1	12
	Receptive language	MCDI, words understood	52	60.5	53	63.7	176.1	121.9	12	100.3	80.2	12
	Expressive language	MCDI, words said	6.8	20.9	6.6	13.7	96.6	118.8	12	44	50.2	12
	Autism symptoms: qualitative impairments in social interaction	ADI, reciprocal social interaction	19.6	3	20.3	4.5	18.3	4.9	12	20.1	4.3	12

Drew et al. (2002)	Autism symptoms: qualitative impairments in communication	ADI, nonverbal communication	12.8	1.6	12	2.4	11	2.8	12	11.9	1.8	12
	Autism symptoms: restricted repetitve and stereotyped patterns of behavior, interests, and activities	ADI, repetitive and stereotyped behavior	3.2	1.1	3.7	1.6	3.9	1.8	12	4.2	2	12
	Parental stress	PSInv., total score	113.8	21.7	110	28.6	104.3	20	12	112.1	20.1	12
Goods et al. (2013)	Functional play	Structured Play Assessment, play types	21.14	7.58	17.13	6.83	22	10.17	5	14.33	9.69	6
	Initiating joint attention	ESCS, initiating joint attention	2.57	4.39	0.75	2.12	0.4	1.89	5	1	1.73	6
	Expressive language	RDLS, expressive language	13.63	4.57	11.93	0.09	14.52	5.38	5	11.95	0.16	6
	Receptive language	RDLS, verbal comprehension	12.14	0.41	12	0.34	14.59	5.36	5	12.05	0.38	6
Green et al. (2010)	Autism general symptom	ADOS-G, total social-communication algorithm score	19.6	4.2	19.3	4.0	15.7	6	77	16.5	5.7	75
	Autism symptoms: qualitative impairments in social interaction	ADOS-G, social domain	10.7	7.2	10.7	2.1	9.2	3	77	9.8	2.9	75
	Autism symptoms: qualitative impairments in communication	ADOS-G, communication domain	8.9	2.5	8.6	2.5	6.6	3.3	77	6.7	3.2	75
	Autism symptoms: restricted repetitve and stereotyped patterns of behavior, interests, and activities	ADOS-G, repetitive-behaviors	3.7	1.5	3.7	1.4	3	1.7	77	3.5	1.6	75
	Receptive language	PLS, receptive raw scores	15.6	9.8	15	9.7	21.5	13	77	20.3	12.8	75
	Expressive language	PLS, expressive raw scores	15	8.1	15.1	7.9	20	11.2	77	20	11.3	75
	Maternal synchronization	Parent-child interaction, parental synchrony	0.32	14.80	0.31	14.60	0.51	19.60	77	0.33	14	75
	Joint engagement	Parent-child interaction, child initiation	0.23	17.40	0.24	18.50	0.35	19.70	77	0.26	17.5	75
Ichikawa et al. (2013)	Parenting stress	PSInd.	226.2	31.5	198.2	26.8	213	34.8	5	204.3	36.3	6
	N/A	BDI	16	7.7	9.8	5.3	12.8	10.3	5	8.5	4.8	6
	Aadaptive behavior	SDQ	19	3.5	13.2	3.3	14.4	4.7	5		12.5	6
	Joint engagement	IRS	38.9	4.8	41.5	3	40.2	5.1	5	39.7	6	6
Ingersoll (2010)	Imitation	UIA					14.93	11.54	11	4.92	4.7	10
Ingersoll (2012)	Initiating joint attention	ESCS: initiating joint attention					5.93	6.72	14	1.46	2.11	13
	Adaptive behavior	SES					101.14	27.98	14	91.08	18.73	13

Kaale et al. (2012)	Initiating joint attention	Joint attention during performing ESCS	1.3	2.8	1.3	1.8	1.6	2.6	33	1.6	2.2	27
	Joint engagement	Joint engagement during mother-child play (%)	45.1	23.4	50.2	21.7	57.3	22.8	34	49.2	19.9	27
Kaale et al. (2014)	Initiating joint attention	Frequency of child initiation of joint attention during performing ESCS	5.71	6.65	5.9	6.67	8.13	6.87	34	6.17	6.87	27
	Receptive language	RDLS, receptive language	20	14.98	25.1	16.25	32.8	10.14	32	39.4	10.14	27
	Expressive language	RDLS, expressive language	14.2	14.15	20.1	17.00	27.9	10.68	32	34.1	10.68	27
	Autism symptoms: qualitative impairment in social	SCQ: C, social subscale, parents					5.28	3.85	29	4.13	3.85	23
	Autism symptoms: qualitative impairment in communication	SCQ: C, communication subscale, parents					4.1	2.16	29	4.52	2.16	23
Kasari et al. (2010)	Joint engagement	Joint engagement	30.26	14.91	24.98	10.74	42.85	19.96	19	27.87	14.01	19
	Initiating joint attention	Frequency of joint attention initiation	3	2.77	3.62	5.92	3.11	3.41	19	3.77	3.76	19
	Responding to joint attention	Frequency of joint attention responses	0.42	0.69	0.63	0.23	0.79	0.23	19	0.05	0.23	19
	Functional play	Type of functional play acts	3	2.38	5.29	2.37	5.29	2.37	19	3.29	2.3	19
	Symbolic play	Type of symbolic play acts	0.11	0.46	0.42	0.84	0.26	0.65	19	0.53	1.43	19
Kim et al. (2008)	Initiating joint attention	ESCS, initiation joint attention	2.4	3.78	5.8	4.87	5.8	6.22	5	9	6.28	5
	Responding to joint attention	ESCS, responding to joint attention	5.2	5.59	7.2	4.82	7.2	6.46	5	9.4	3.78	5
Landa et al. (2011)	Imitation	Socially engaged imitation	0.17	0.19	0.25	0.24	0.44	0.22	24	0.28	0.21	24
	Initiating joint attention	ESCS, initiation joint attention	2.4	3.78	5.8	4.87	5.8	6.22	24	9	6.28	24
	Responding to joint attention	ESCS, responding to joint attention	5.2	5.59	7.2	4.82	7.2	6.46	24	9.4	3.78	24
	Joint engagement	CSBCDP, shared positive affect	2.42	2.93	3.54	3.56	4.96	5.48	24	7.57	9.76	24
	Expressive language	MSEL, expressive language	23.92	5.5	25.92	8.12	31.36	12.12	24	34.52	12.33	24
	Developmental quotient	MSEL, nonverbal cognition	27.5	8.27	31.12	9.86	30.28	16.62	24	34.44	16.67	24
Lawton et al. (2012)	Initiating joint attention	ESCS: frequency of initiating joint attentionl	11.89	10.01	13.29	7.34	15.33	10.89	9	9	7.23	7
	Joint engagement	Supported engagement	4.27	1.77	5.58	0.81	5.58	0.81	9	4.11	1.68	7
Pajareya et al. (2011)	Developmental quotient	FEDQ	40.69	15.34	44	12.92	44	12.88	16	44.8	13.4	44
	Autism symptoms: general	CARS	39.69	6.59	35.25	6.08	37.18	6.22	16	34.5	6.01	16
	Adaptive behavior	VABS, adaptive composite	59.3		64		62.7	6.8	7	63.3	5.7	4

Reitzel et al.<br>(2013)	Joint attention responding	FBSA, response to name when sitting	2		2		1.7	1.3	7	1.6	2.1	4
	Communication	VABS, communication	64.4	12.8	68.5	17	68.4	7.7	26	74.2	15.5	14
Roberts et al.<br>(2011)<br>Home-based	Socialization	VABS, socialization	68.7	7.3	70.8	9.9	66.4	7.7	27	73.1	10.8	14
	Receptive language	RDLS, comprehension	4.2	9.2	7.2	15.2	2.6	8.4	27	5.7	12.1	14
	Expressive language	RDLS, expression	3.4	8.3	6	10.9	2.8	7.5	26	4.4	8.7	14
	Adaptive behavior	DBC	44.7	19	43.9	21.9	52.9	29.3	22	42.9	24.3	13
	Parental stress	PSInd.	90.4	18.9	97.1	22	92.7	20.9	20	90.4	22.9	11
	Communication	VABS, communication	66.9	12.5	68.5	17	76.1	17.1	29	74.2	15.5	14
Roberts et al.<br>(2011)<br>Center-based	Socialization	VABS, socialization	70.1	7.3	70.8	9.9	72.6	11.2	29	73.1	10.8	14
	Receptive language	RDLS, comprehension	5.5	10.6	7.2	15.2	10.5	17.4	26	5.7	12.1	14
	Expressive language	RDLS, expression	8.2	16.6	6	10.9	7	15.1	26	4.4	8.7	14
	Adaptive behavior	DBC	58.5	20.4	43.9	21.9	55.7	19.5	22	42.9	24.3	13
	Parental stress	PSInd.	103.4	19.6	97.1	22	98.2	20.1	20	90.4	22.9	11
	Autism symptoms: qualitative impairments in social interaction	Modified ADOS: social affect	29.45	9.16	34.14	8.69	26.61	10.14	49	27.33	10.62	49
Rogers et al.<br>(2012)	Autism symptoms: restricted repetitve and stereotyped patterns of behavior, interests, and activities	ADOS, restrictive and repetitive	3.92	2.01	4.31	1.92	3.96	1.86	49	3.82	2.04	49
	Developmental quotient	MSEL, developmental quotient	64.88	17.22	63.08	15.93	69.82	17.9	49	67.92	17.93	49
	Receptive language	MCDI, phrases understood	8.22	7.02	9.38	7.95	12.73	9.11	49	14.77	8.14	49
	Expressive language	MCDI, vocabulary production	12.24	35.6	12.44	39.72	42.27	61.99	49	38.87	73.71	49
	Communication	VABS, communication	67.66	13.19	67.29	11.05	72.55	12.06	49	74.29	14.55	49
	Socialization	VABS, socialization	76.68	8.74	77.95	8.01	77.32	9.19	49	78.67	10.78	49
	Adaptive behavior	VABS, composite	76.76	10.3	78.22	8.88	77.43	9.59	49	80.33	11.34	49
	Imitation	Imitative sequences	3.78	3.12	2.53	2.6	4.58	3.45	49	3.76	3.44	49
	Responding to joint attention	Orient to joint attention	0.35	0.35	0.28	0.33	0.34	0.29	49	0.34	0.34	49
	Responding to joint attention	Observational, responding to initiating joint attention	0.06	0.13	0.25	0.32	6.29	5.53	11	0.58	0.68	12
	Initiating joint attention	Observational; initiating joint attention	0.14	0.26	0.19	0.33	3.27	3.17	11	0.86	0.77	12
	Communication	VABS, communication	75.9	13.51	68.08	19.77	75.9	13.51	11	68.08	19.77	12
	Receptive language	MSEL, receptive language	28.27	11.35	25.33	8.52	28.27	11.35	11	25.33	8.52	12
	Expressive language	MSEL, expressive language	33.27	15.79	27.17	11.21	33.27	15.79	11	27.17	11.21	12
	Maternal synchronization	Maternal synchronization	0.57	0.03	0.63	0.03	0.72	0.04	34	0.61	0.04	30

Siller et al. (2013)	Expressive language	MSEL, expressive language	3.7	0.16	3.75	0.16	4.02	0.16	34	3.9	0.17	30
	Joint engagement	mean of P/CSBS and ABS	3.47	1.67	3.83	1.8	3.81	1.62	31	3.05	1.54	28
	Developmental quotient	BSID, mental development index	50.53	11.18	50.69	13.88	66.49	24.08	15	49.67	19.74	13
Smith et al. (2000)	Receptive language	RDLS, comprehension	13.47	3.6	13.69	3.73	42.87	22.29	15	33	16.86	13
	Expressive language	RDLS, expressive	15.13	0.52	16.31	2.69	44.53	23.48	15	36.23	21.19	13
	Adaptive behavior	VABS, composite	63.44	9.35	65.17	9.44	61.19	29.72	15	58.5	16.58	13
	Stress thermometer ratings	Parenting stress	2.51	0.82	2.06	0.87	1.86	0.88	35	2.26	1.09	35
Tonge et al., (2006)	Developmental quotient	PEP-R, developmental quotient	64.74	27.41	63.31	28.52	72.18	24.77	35	67.72	28.14	35
Tonge et al. (2014)	Receptive language	RDLS, comprehension	9.8	15.72	14.47	21.32	14.06	19.67	35	19.18	22.17	35
Tonge et al. (2014)	Expressive language	RDLS, expression	14.77	16.83	14.41	18.24	17.17	17.07	35	18.24	20.65	35
	Autism symptoms: general	CARS	41.23	5.25	38.97	5.79	35.86	6.14	35	38.73	6.73	35
	Joint engagement	Communication acts	0.29	0.49	2	2.24	9.71	14.08	7	1.86	2.67	7
Venker et al. (2011)	Maternal synchronization	Parent redirecting behaviours	14	8.58	12.29	10	4.29	3.35	7	14.29	15.39	7
	Developmental quotient	MSEL, developmental quotient	57.1	15.6	53.7	24.5	63.7	17.4	10	58.1	25	10
Welterlin et al. (2012)	Parental stress	PSInd., total score	254.1	48.5	244.5	32.8	242.4	41.5	10	256.2	70	10
	Joint engagement	SIB-R, social interaction	11.8	6.1	12.1	4.2	18.4	7.3	10	16	5	10
	Receptive language	SIB-R, language comprehension	9.9	4.4	7.3	4.2	12	4.7	10	10.9	4.8	10
	Expressive language	SIB-R, language expression	11.4	5.7	10.2	7.5	16.2	7.1	10	14.2	7.1	10

ADOS indicates Autism Diasnostic Observation Schedule. MSEL indicates Mullen Scales of Early Learning. VABS indicates Vineland Adaptive Behavior Scale. ESCS indicates Early Social Communication Scale. PCFP indicates Parent-Child Free Play Procedur. PIA-CV indicates Parent Interview for Autism-Clinincal Version. CBRS indicates Child Behavior Rating Scale. RBS indicates Repetitive Behavior Scale. GSMD indicates Griffiths Scale of Mental Development. MCDI indicates MacArthur-Bates Communicative Developmental Inventory. ADI indicates Autism Diagnostic Interview. PSInv. indicates Parental Stress Inventory. RDLS indicates Reynell Developmental Language Scales. PLS indicates Preschool Language Scale IV. PSInd. indicates Parental Stress Index. SDQ indicates Strengths and Difficulties Questionnaire. IRS indicates Inteaction Rating Scale. UIA indicates Unstructured Imitation Assessment. SES indicates Social Emotion Scale. SCQ: C indicates Social Communication Questionnaire: Current Form. DAS indicates Differential Abilities Scale. CSBDP indicates Communication and Symbolic Behavior Scales Developmental Profile. CARS indicates Childhood Autism Rating Scale. FEDQ indicates Functional Emotional Developmental Questionnaire. FBSA indicates Functional Behavior Skills Assessment. DBC indicates Developmental Behaviour Checklist. BSID indicates Bayley Scales of Infant Development. PEP-R indicates Psychoeducational Profile-Revised. SIB-R indicates Scales of Independent Behavior-Revised.
