# Supplementary material for: A systematic review and meta-analysis of comprehensive interventions for pre-school children with autism spectrum disorder (ASD)
Source: PLoS One. 2017 Dec 6;12(12):e0186502. doi: 10.1371/journal.pone.0186502 (PMC5718481; doi:10.1371/journal.pone.0186502)
Supplement: S4 Table — (PDF) [file pone.0186502.s006.pdf]

S4 Table. Comparisons of the results of the meta-analyses with those of the sensitivity analyses

			Random effects model		Fixed effects model			
			Analysis II		Analyses III		Analyses III	
	Outcome		p value	SMD (95%CI)	p value	SMD (95%CI)	p value	SMD (95%CI)
Primary outcome	Autism general symptoms	○	<0.01**	-0.30[-0.50,-0.09]	0.04*	-0.27[-0.53,-0.01]	<0.01**	-0.30[-0.50,-0.09]
Secondary outcomes	Developmental quotient	◎	0.02*	0.22[0.04,0.41]	0.01*	0.33[0.07,0.52]	0.02*	0.22[0.04,0.41]
	Developmental quotient (sensitivity analysis)		0.04*	0.20[0.00,0.39]	0.04*	0.29[0.01,0.56]	0.04*	0.20[0.00,0.39]
	Expressive language	○	0.03*	0.16[0.02,0.31]	0.23	0.11[-0.07,0.30]	0.03*	0.16[0.02,0.31]
	Expressive language (sensitivity analysis)	○	0.02*	0.18[0.03,0.33]	0.18	0.13[-0.06,0.33]	0.02*	0.18[0.03,0.33]
	Receptive language		0.50	0.06[-0.11,0.23]	0.33	0.09[-0.09,0.28]	0.55	0.05[-0.11,0.23]
	Receptive language (sensitivity analysis)		0.25	0.10[-0.07,0.28]	0.33	0.09[-0.09,0.28]	0.27	0.09[-0.07,0.26]
	Reciprocity of social intercation towards others	◎	<0.001***	0.44[0.24,0.64]	<0.001***	0.51[0.31,0.72]	<0.001***	0.43[0.26,0.61]
	Reciprocity of social intercation towards others (Sensitivity analysis)		<0.001***	0.53[0.29,0.78]	<0.001***	0.51[0.31,0.72]	<0.001***	0.51[0.31,0.72]
	Adaptive behaviour		0.24	0.15[-01.0,0.39]	0.69	-0.04[-0.23,0.15]	1.00	0.17[-0.08,0.43]
	Adaptive behaviour (Sensitivity analysis)		0.45	0.09[-0.15,0.34]	0.69	-0.04[-0.23,0.15]	0.78	0.03[-0.16,0.22]
Other outcomes	Qualitative impairment in social interaction		0.63	-0.07[-0.33,0.20]	0.24	-0.15[-0.40,0.10]	0.45	-0.08[-0.30,0.14]
	Qualitative impairment in communication		0.60	-0.07[-0.33,0.19]	0.85	-0.03[-0.35,0.29]	0.85	-0.03[-0.35,0.29]
	Restricted repetitive and stereotyped patterns of behaviour, interests, and activities		0.11	-0.18[-0.40,0.04]	0.22	-0.15[-0.40,0.09]	0.11	-0.18[-0.40,0.04]
	Initiating joint attention	○	<0.01*	0.35[0.10,0.61]	<0.01**	0.32[0.08,0.55]	<0.01**	0.32[0.12,0.51]
	Responding to joint attention		0.11	0.58[-0.12,1.28]	<0.001***	0.32[0.22,0.41]	<0.001***	0.32[0.22,0.41]
	Imitation	○	0.02*	0.56[0.09,1.03]	0.05	0.37[-0.00,0.73]	<0.01**	0.47[0.16,0.78]
	Symbolic play		0.86	0.08[-0.80,0.95]		N/A	0.89	-0.04[-0.61,0.53]
	Functional play	○	<0.01*	0.81[0.22,1.40]		N/A	<0.01**	0.81[0.22,1.40]
	Parental synchrony	◎	<0.01*	0.98[0.30,1.66]	<0.001***	0.99[0.70,1.29]	<0.001***	1.06[0.82,1.30]
	Parenting stress		0.15	-0.30[-0.69,0.10]	0.15	-0.30[-0.69,0.10]	0.15	-0.30[-0.69,0.10]

p value indicates the value of the test of overall synthesis. SMD indicates standard mean difference of the overall synthesis effect. 95% CI indicates the 95% confidence interval of the standard mean difference of the overall synthesis. *, **, and *** indicate statistically significant effectiveness ($p<0.05$, $p<0.01$, and $P<0.001$, respectively) in the analysis. ○ indicates the outcome did not show significant effectiveness in the overall synthesis of Analysis I, but showed significant effectiveness in the sensitivity analysis. ◎ indicates the outcome showed significant effectiveness in both the overall synthesis of Analysis I and its sensitivity analyses; Analysis II, III, and IV. N/A indicates the analysis with overall synthesis could not be performed because only one study measured the outcome.
