# Supplementary material for: A systematic review and meta-analysis of comprehensive interventions for pre-school children with autism spectrum disorder (ASD)
Source: PLoS One. 2017 Dec 6;12(12):e0186502. doi: 10.1371/journal.pone.0186502 (PMC5718481; doi:10.1371/journal.pone.0186502)
Supplement: S5 Table — (PDF) [file pone.0186502.s007.pdf]

S5 Table. Comparisons of each model condition versus control condition

Outcomes		Study ID	Results: Analysis I SMD	Analysis I 95%CI	Analysis I Subtotal SMD	Analysis I Subtotal 95%CI	Analysis I p value	Analysis II SMD	Analysis II 95%CI	Analysis II Subtotal SMD	Analysis II Subtotal 95%CI	Analysis II p value
Autism general symtom	Behavioural model	N/A	N/A	N/A	N/A	N/A	N/A	N/A	N/A	N/A	N/A	N/A
	Social-communication focused model	Aldred 2004	-0.77	[-1.54,0.00]	-0.28	[-0.62,0.05]	0.10	-0.76	[-1.53,0.01]	-0.35	[-0.92,0.23]	0.24
		Drew 2002	-0.38	[-1.19,0.43]				N/A	N/A			
		Green 2010	-0.14	[-0.45,0.18]				-0.14	[-0.45,0.18]			
	Multimodal developmental model	Dawson 2010	-0.16	[-0.75,0.43]	-0.35	[-0.65,0.06]	0.02	-0.43	[-1.01,0.15]	-0.43	[-1.01,015]	0.14
		Jocelyn1998	-0.40	[-1.08,0.27]				N/A	N/A			
		Pajareya 2011	-0.38	[-1.08,0.32]				N/A	N/A			
		Tonge 2006	-0.44	[-0.92,0.03]				N/A	N/A			
Developmental quotient	Behavioural model	Smith 2000	0.74	[-0.04,1.51]	0.74	[-0.04,1.51]	0.06	0.74	[-0.04,1.51]	0.74	[-0.04,1.51]	0.06
	Social-communication focused model	Carter 2011	-0.06	[-0.61,0.49]	0.22	[-0.18,0.61]	0.28	N/A	N/A	0.71	[-0.12,1.54]	0.09
		Drew 2002	0.71	[-0.12,1.54]				0.71	[-0.12,1.54]			
		Landa 2011	0.25	[-0.32,0.81]				N/A	N/A			
	Multimodal developmental intervention	Dawson 2010	0.59	[-0.01,1.19]	0.18	[-0.04,0.41]	0.11	0.63	[0.04,1.22]	0.23	[-0.11,0.58]	0.19
		Jocelyn1998	0.02	[-0.65,0.68]				0.02	[-0.65,0.68]			
		Pajareya 2011	0.06	[-0.63,0.75]				N/A	N/A			
		Rogers 2012	0.11	[-0.29,0.50]				0.11	[-0.29,0.50]			
		Tonge 2006	0.17	[-0.30,0.64]				N/A	N/A			
		Welterlin 2012	0.25	[-0.63,1.13]				N/A	N/A			
Developmental quotient (Sensitivity analysis)	Behavioural model	Smith 2000	0.74	[-0.04,1.51]	0.74	[-0.04,1.51]	0.06	0.74	[-0.04,1.51]	0.74	[-0.04,1.51]	0.06
	Social-communication focused model	Carter 2011	-0.06	[-0.61,0.49]	0.09	[-0.31,0.49]	0.66	N/A	N/A	N/A	N/A	N/A
		Drew 2002	0.71	[-0.12,1.54]				0.71	[-0.12,1.54]			
		Landa 2011	0.25	[-0.32,0.81]				N/A	N/A			
	Multimodal developmental intervention	Dawson 2010	0.59	[-0.01,1.19]	0.18	[-0.04,0.41]	0.11	0.63	[0.04,1.22]	0.23	[-0.11,0.58]	0.19
		Jocelyn1998	0.02	[-0.65.068]				0.02	[-0.65,0.68]			
		Pajareya 2011	0.06	[-0.63,0.75]				N/A	N/A			
		Rogers 2012	0.11	[-0.29,0.50]				0.11	[-0.29,0.50]			
		Tonge 2006	0.17	[-0.30,0.64]				N/A	N/A			
		Welterlin 2012	0.25	[-0.63,1.13]				N/A	N/A			

Expressive language	Behavioural model	Smith 2000	0.36	[-0.39,1.11]	0.36	[-0.39,1.11]	0.35	0.36	[-0.39,1.11]	0.36	[-0.39,1.11]	0.35
	Social-communication focused model	Aldred 2004	0.01	[-0.73,0.75]	0.17	[-0.01,0.35]	0.07	0.33	[-0.41,1.08]	0.09	[-0.14,0.33]	0.44
		Carter 2011	-0.06	[-0.62,0.49]				N/A	N/A			
		Drew 2002	0.56	[-0.26,1.38]				0.56	[-0.26,1.38]			
		Goods 2013	0.65	[-058,1.89]				N/A	N/A			
		Green 2010	0.00	[-0.32,0.32]				0.00	[-0.32,0.32]			
		Kaale 2012	-0.03	[-0.54,0.48]				-0.03	[-0.54,0.48]			
		Landa 2011	0.15	[-0.42,0.72]				N/A	N/A			
		Schertz 2013	0.43	[-0.40,1.26]				N/A	N/A			
		Siller 2013	0.72	[0.20,1.24]				N/A	N/A			
		Thompson2012	0.27	[-0.59,1.13]				0.27	[-0.59,1.13]			
	Multimodal developmental intervention	Dawson 2010	0.55	[-0.05,1.15]	0.13	[-0.13,0.39]	0.32	0.24	[-0.34,0.81]	0.11	[-0.22,0.44]	0.51
		Roberts 2011 Center -based	0.19	[-0.46,0.84]				N/A	N/A			
		Roberts 2011 Home-based	-0.11	[-0.76,0.54]				N/A	N/A			
		Rogers 2012	0.05	[-0.35,0.45]				0.05	[-0.35,0.45]			
		Tonge 2006	-0.06	[-0.52,0.41]				N/A	N/A			
		Welterlin 2012	0.27	[-0.61,1.15]				N/A	N/A			
Expressive language (Sensitivity analysis)	Behavioural model	Smith 2000	0.36	[-0.39,1.11]	0.36	[-0.39,1.11]	0.35	0.36	[-0.39,1.11]	0.36	[-0.39,1.11]	0.35
	Social-communication focused model	Aldred 2004	0.01	[-0.73,0.75]	0.19	[0.00,0.39]	0.05	0.33	[-0.41,1.08]	0.12	[-0.14,0.39]	0.36
		Carter 2011	-0.06	[-0.62,0.49]				N/A	N/A			
		Drew 2002	0.56	[-0.26,1.38]				0.56	[-0.26,1.38]			
		Goods 2013	0.65	[-058,1.89]				N/A	N/A			
		Green 2010	0.00	[-0.32,0.32]				0.00	[-0.32,0.32]			
		Landa 2011	0.15	[-0.42,0.72]				N/A	N/A			
		Schertz 2013	0.43	[-0.40,1.26]				N/A	N/A			
		Siller 2013	0.72	[0.20,1.24]				N/A	N/A			
		Thompson2012	0.27	[-0.59,1.13]				0.27	[-0.59,1.13]			
	Multimodal developmental intervention	Dawson 2010	0.55	[-0.05,1.15]	0.18	[0.03,0.33]	0.32	0.24	[-0.34,0.81]	0.11	[-0.22,0.44]	0.51
		Rogers 2012	0.05	[-0.35,0.45]				0.05	[-0.35,0.45]			
		Tonge 2006	-0.06	[-0.52,0.41]				N/A	N/A			
		Welterlin 2012	0.27	[-0.61,1.15]				N/A	N/A			

Receptive language	Behavioural model	Smith 2000	0.48	[-0.28,1.23]	0.48	[-0.28,1.23]	0.21	0.48	[-0.28,1.23]	0.48	[-0.28,1.23]	0.21
	Social-communication focused model	Aldred 2004	0.00	[-0.74,0.74]				0.00	[-0.74,0.74]			
		Carter 2011	-0.25	[-0.81,0.30]				N/A	N/A			
		Drew 2002	0.71	[-0.12,1.54]				0.71	[-0.12,1.54]			
		Goods 2013	0.65	[-0.59,1.88]	0.07	[-0.13,0.28]	0.49	N/A	N/A	0.09	[-0.14,0.33]	0.44
		Green 2010	0.09	[-0.23,0.41]				0.09	[-0.23,0.41]			
		Kaale 2012	-0.14	[-0.65,0.37]				-0.14	[-0.65,0.37]			
		Schertz 2013	0.28	[-0.54,1.11]				N/A	N/A			
		Thompson2012	0.20	[-0.66,1.06]				0.20	[-0.66,1.06]			
	Multimodal developmental intervention	Dawson 2010	0.60	[-0.00,1.20]				0.57	[-0.02,1.15]			
		Roberts 2011 Center -based	0.30	[-0.36,0.95]				N/A	N/A			
		Roberts 2011 Home-based	-0.31	[-0.96,0.34]	0	[-0.29,0.30]	0.98	N/A	N/A	0.14	[-0.65,0.92]	0.73
		Rogers 2012	-0.23	[-0.63,0.16]				-0.23	[-0.63,0.16]			
		Tonge 2006	-0.24	[-0.71,0.23]				N/A	N/A			
		Welterlin 2012	0.22	[-0.66,1.10]				N/A	N/A			
Receptive language (Sensitivity analysis)	Behavioural model	Smith 2000	0.48	[-0.28,1.23]	0.48	[-0.28,1.23]	0.21	0.48	[-0.28,1.23]	0.48	[-0.28,1.23]	0.21
	Social-communication focused model	Aldred 2004	0.00	[-0.74,0.74]				0.00	[-0.74,0.74]			
		Carter 2011	-0.25	[-0.81,0.30]				N/A	N/A			
		Drew 2002	0.71	[-0.12,1.54]				0.71	[-0.12,1.54]			
		Goods 2013	0.65	[-0.59,1.88]	0.07	[-0.13,0.28]	0.49	N/A	N/A	0.09	[-0.14,0.33]	0.44
		Green 2010	0.09	[-0.23,0.41]				0.09	[-0.23,0.41]			
		Kaale 2012	-0.14	[-0.65,0.37]				-0.14	[-0.65,0.37]			
		Schertz 2013	0.28	[-0.54,1.11]				N/A	N/A			
		Thompson2012	0.20	[-0.66,1.06]				0.20	[-0.66,1.06]			
	Multimodal developmental intervention	Dawson 2010	0.60	[-0.00,1.20]				0.57	[-0.02,1.15]			
		Roberts 2011 Center -based	0.30	[-0.36,0.95]	0.08	[-0.220.37]	0.61	N/A	N/A	0.14	[-0.65,0.92]	0.73
		Rogers 2012	-0.23	[-0.63,0.16]				-0.23	[-0.63,0.16]			
Reciprocity of social interaction towards others	Behavioural model	N/A	N/A	N/A	N/A	N/A	N/A	N/A	N/A	N/A	N/A	N/A
	Social-communication [illegible]	Aldred 2004	0.71	[-0.06,1.48]				0.71	[-0.06,1.48]			
		Green 2010	0.47	[0.15,0.80]				0.47	[0.15,0.80]			

	focused model	Ichikawa 2013	0.08	[-1.11,1.27]				0.08	[-1.11,1.27]			
		Kaale 2012	0.37	[-0.14,0.88]				0.37	[-0.14,0.88]			
		Kasari2010	0.85	[0.18,1.52]				0.85	[0.18,1.52]			
		Kim 2008	0.54	[-0.73,1.82]	0.5	[0.24,0.75]	0.0001	N/A	N/A	0.58	[0.36,0.81]	<0.00001
		Landa 2011	-0.32	[-0.89,0.25]				N/A	N/A			
		Lawton 2012	1.10	[0.02,2.19]				1.10	[0.02,2.19]			
		Siller2013	0.47	[-0.04,0.99]				N/A	N/A			
		Thompson2012	1.36	[0.44,2.29]				1.36	[0.44,2.29]			
		Venker 2012	0.73	[-0.37,1.82]				N/A	N/A			
	Multimodal developmental intervention	Casenhiser 2011	0.11	[-0.62,0.84]				0.11	[-0.44,0.66]			
		Welterlin 2012	0.37	[-0.52,1.25]	0.24	[-0.20,0.68]	0.28	N/A	N/A	0.11	[-0.44,0.66]	0.69
		Pajareya 2011	0.28	[-0.42,0.99]				N/A	N/A			
Reciprocity of social interaction towards others(Joint engagement) (Sensitivity analysis)	Behavioural model	N/A	N/A	N/A	N/A	N/A	N/A	N/A	N/A	N/A	N/A	N/A
	Social-communication focused model	Aldred 2004	0.71	[-0.06,1.48]				0.71	[-0.06,1.48]			
		Green 2010	0.47	[0.15,0.80]				0.47	[0.15,0.80]			
		Ichikawa 2013	0.08	[-1.11,1.27]				0.08	[-1.11,1.27]			
		Kaale 2012	0.37	[-0.14,0.88]				0.37	[-0.14,0.88]			
		Kasari2010	0.85	[0.18,1.52]	0.56	[0.36,0.76]	<0.001	0.85	[0.18,1.52]	0.58	[0.36,0.81]	<0.00001
		Kim 2008	0.54	[-0.73,1.82]				N/A	N/A			
		Lawton 2012	1.10	[0.02,2.19]				1.10	[0.02,2.19]			
		Siller2013	0.47	[-0.04,0.99]				N/A	N/A			
		Thompson2012	1.36	[0.44,2.29]				1.36	[0.44,2.29]			
	Multimodal developmental intervention	Casenhiser 2011	0.11	[-0.62,0.84]				0.11	[-0.44,0.66]			
		Welterlin 2012	0.37	[-0.52,1.25]	0.24	[-0.20,0.68]	0.28	N/A	N/A	0.11	[-0.44,0.66]	0.69
		Pajareya 2011	0.28	[-0.42,0.99]				N/A	N/A			
Adaptive behavior	Behavioural model	Reitzel 2013	-0.08	[-1.47,1.30]	0.06	[-0.59,0.72]	0.85	N/A	N/A	0.11	[-0.64,0.85]	0.78
		Smith 2000	0.11	[-0.64,0.85]				0.11	[-0.64,0.85]			
	Social-communication focused model	Green 2010	-0.17	[-0.48,0.15]	0.01	[-0.51,0.53]	0.97	-0.17	[-0.48,0.15]	0.01	[-0.51,0.53]	0.97
		Ingersoll 2012	0.41	[-0.36,1.17]				0.41	[-0.36,1.17]			
	Multimodal	Casenhiser 2011	0.14	[-0.59,0.87]				0.15	[-0.40,0.69]			
		Dawson 2010	0.72	[0.11,1.33]				0.21	[-0.36,0.78]			

	Multimodal developmental intervention	Roberts 2011 Center -based	0.59	[-0.12,1.29]	0.27	[-0.11,0.65]	0.16	N/A	N/A	-0.02	[-0.30,0.26]	0.90
		Roberts 2011 Home-based	0.35	[-0.34,1.05]				N/A	N/A			
		Ichikawa 2013	0.44	[-0.77,1.65]				0.44	[-0.77,1.65]			
		Rogers 2012	-0.27	[-0.67,0.12]				-0.27	[-0.67,0.12]			
Adaptive behavior (Sensitivity analysis)	Behavioural model	Reitzel 2013	-0.08	[-1.47,1.30]	0.06	[-0.59,0.72]	0.85	N/A	N/A	0.11	[-0.64,0.85]	0.78
		Smith 2000	0.11	[-0.64,0.85]				0.11	[-0.64,0.85]			
	Social-communication focused model	Green 2010	-0.17	[-0.48,0.15]	0.01	[-0.51,0.53]	0.97	-0.17	[-0.48,0.15]	0.01	[-0.51,0.53]	0.97
		Ingersoll 2012	0.41	[-0.36,1.17]				0.41	[-0.36,1.17]			
	Multimodal developmental intervention	Casenhiser 2011	0.14	[-0.59,0.87]	0.22	[-0.21,0.64]	0.32	0.15	[-0.40,0.69]	-0.02	[-0.30,0.26]	0.90
		Dawson 2010	0.72	[0.11,1.33]				0.21	[-0.36,0.78]			
		Roberts 2011 Home-based	0.35	[-0.34,1.05]				N/A	N/A			
		Ichikawa 2013	0.44	[-0.77,1.65]				0.44	[-0.77,1.65]			
		Rogers 2012	-0.27	[-0.67,0.12]				-0.27	[-0.67,0.12]			
Autism symptom :qualitative impairments in social interaction	Behavioural model	N/A	N/A	N/A	N/A	N/A	N/A	N/A	N/A	N/A	N/A	N/A
	Social-communication focused model	Carter 2011	0.41	[-0.15,0.97]	-0.05	[-0.48,0.39]	0.84	0.41	[-0.15,0.97]	-0.23	[-0.52,0.07]	0.14
		Drew 2002	-0.38	[-1.19,0.43]				-0.38	[-1.19,0.43]			
		Green 2010	-0.20	[-0.52,0.12]				-0.20	[-0.52,0.12]			
	Multimodal developmental intervention	Rogers 2012	-0.07	[-0.46,0.33]	-0.07	[-0.46,0.33]	0.73	-0.07	[-0.46,0.33]	-0.07	[-0.46,0.33]	0.73
Autism symptom :qualitative impairments in communication	Behavioural model	N/A	N/A	N/A	N/A	N/A	N/A	N/A	N/A	N/A	N/A	N/A
	Social-communication focused model	Carter 2011	-0.04	[-0.62,0.53]	-0.07	[-0.33,0.19]	0.60	N/A	N/A	-0.08	[-0.37,0.22]	0.61
		Drew 2002	-0.37	[-1.18,0.44]				-0.37	[-1.18,0.44]			
		Green 2010	-0.03	[-0.35,0.29]				-0.03	[-0.35,0.29]			
	Multimodal developmental intervention	N/A	N/A	N/A	N/A	N/A	N/A	N/A	N/A	N/A	N/A	N/A
Autism symptom :restricted repetitive and stereotyped [illegible]	Behavioural model	N/A	N/A	N/A	N/A	N/A	N/A	N/A	N/A	N/A	N/A	N/A

patterns of behavior, interests, and activities	Social-communication focused model	Drew 2002	-0.15	[-0.95,0.65]	-0.28	[-0.58,0.02]	0.06	-0.15	[-0.95,0.65]	-0.28	[-0.58,0.02]	0.06
		Green 2010	-0.30	[-0.62,0.02]				-0.30	[-0.62,0.02]			
	Multimodal developmental intervention	Dawson 2010	-0.35	[-0.95,0.24]	-0.08	[-0.48,0.32]	0.68	-0.51	[-1.09,0.07]	-0.18	[-0.74,0.38]	0.54
		Rogers 2012	0.07	[-0.32,0.47]				0.07	[-0.32,0.47]			
Initiating joint attention	Behavioural model	N/A	N/A	N/A	N/A	N/A	N/A	N/A	N/A	N/A	N/A	N/A
	Social-communication focused model	Carter 2011	0.17	[-0.39,0.73]	0.29	[0.01,0.56]	0.04	N/A	N/A	0.23	[-0.23,0.70]	0.33
		Goods 2013	-0.39	[-1.59,0.82]				N/A	N/A			
		Ingersoll 2012	0.86	[0.06,1.65]				0.86	[0.06,1.65]			
		Kaale 2012	0.00	[-0.51,0.51]				0.00	[-0.51,0.51]			
		Kasari 2010	-0.18	[-0.82,0.46]				-0.18	[-0.82,0.46]			
		Kim 2008	0.46	[-0.80,1.73]				N/A	N/A			
		Landa 2011	0.44	[-0.14,1.01]				N/A	N/A			
		Lawton 2012	0.63	[-0.39,1.65]				0.63	[-0.39,1.65]			
		Schertz 2013	1.03	[0.15,1.91]				N/A	N/A			
	Multimodal developmental intervention	Casenhiser 2011	1.19	[0.38,1.99]	0.64	[-0.28,1.57]	0.17	1.22	[0.62,1.82]	0.65	[-0.42,1.72]	0.24
		Rogers 2012	0.24	[-0.16,0.63]				0.13	[-0.27,0.52]			
Responding to joint attention	Behavioural model	Reitzel 2013	0.10	[-1.73,1.93]	0.10	[-1.73,1.93]	0.91	N/A	N/A	N/A	N/A	N/A
	Social-communication focused model	Kasari2010	0.74	[0.59,0.89]	2.72	[-1.02,6.47]	0.15	0.74	[0.59,0.89]	2.20	[-1.07,5.48]	0.19
		Kim 2008	2.20	[-4.36,8.76]				0.45	[-4.05,4.95]			
		Schertz 2013	5.71	[2.42,9.00]				5.71	[2.42,9.00]			
	Multimodal developmental intervention	Rogers 2012	0.00	[-0.13,0.13]	0.00	[-0.13,0.13]	1.00	0.00	[-0.13,0.13]	0.00	[-0.13,0.13]	1.00
Imitation	Behavioural model	N/A	N/A	N/A	N/A	N/A	N/A	N/A	N/A	N/A	N/A	N/A
	Social-communication focused model	Ingersoll 2010	1.07	[0.14,2.00]	0.83	[0.33,1.32]	0.001	1.07	[0.14,2.00]	1.07	[0.14,2.00]	0.02
		Landa 2011	0.73	[0.15,1.32]				N/A	N/A			
	multimodal developmental ntervention	Rogers 2012	0.24	[-0.16,0.63]	0.24	[-0.16,0.63]	0.24	0.24	[-0.16,0.63]	0.24	[-0.16,0.63]	0.24

Symbolic play	Behavioural model	N/A	N/A	N/A	N/A	N/A	N/A	N/A	N/A	N/A	N/A	N/A
	Social-communication focused model	Goods 2013	0.71	[-0.54,1.95]	0.08	[-0.80,0.95]	0.86	N/A	N/A	-0.24	[-0.88,0.40]	0.46
		Kasari 2010	-0.24	[0.88,0.40]				-0.24	[0.88,0.40]			
	Multimodal developmental intervention	N/A	N/A	N/A	N/A	N/A	N/A	N/A	N/A	N/A	N/A	N/A
Functional play	Behavioural model	N/A	N/A	N/A	N/A	N/A	N/A	N/A	N/A	N/A	N/A	N/A
	Social-communication focused model	Goods 2013	0.71	[-0.54,1.95]	0.81	[0.22,1.40]	0.007	N/A	N/A	0.84	[0.17,1.50]	0.01
		Kasari 2010	0.84	[0.17,1.50]				0.84	[0.17,1.50]			
	Multimodal developmental intervention	N/A	N/A	N/A	N/A	N/A	N/A	N/A	N/A	N/A	N/A	N/A
Parental synchrony	Behavioural model	N/A	N/A	N/A	N/A	N/A	N/A	N/A	N/A	N/A	N/A	N/A
	Social-communication focused model	Aldred 2004	0.90	[0.12,1.69]	0.98	[0.30,1.66]	0.005	0.90	[0.12,1.69]	0.99	[0.70,1.29]	<0.00001
		Carter 2011	0.45	[-0.13,1.03]				N/A	N/A			
	Multimodal developmental intervention	Green 2010	1.09	[0.75,1.43]				1.09	[0.75,1.43]			
		Siller 2013	2.72	[2.03,3.41]				N/A	N/A			
		Thompson2012	0.42	[-0.50,1.33]				0.42	[-0.50,1.33]			
		Venker 2012	0.04	[-1.00,1.09]				N/A	N/A			
		N/A	N/A	N/A	N/A	N/A	N/A	N/A	N/A	N/A	N/A	N/A
Parenting stress	Behavioural model	N/A	N/A	N/A	N/A	N/A	N/A	N/A	N/A	N/A	N/A	N/A
	Social-communication focused model	Drew 2002	-0.37	[-1.26,0.51]	-0.37	[-1.26,0.51]	0.41	-0.37	[-1.26,0.51]	-0.37	[-1.26,0.51]	0.41
	Multimodal developmental intervention	Ichikawa 2013	0.22	[-0.97,1.42]	-0.06	[-0.40,0.28]	0.72	0.22	[-0.97,1.42]	-0.28	[-0.72,0.17]	0.22
		Roberts 2011 Center -based	0.36	[-0.38,1.10]				N/A	N/A			
		Roberts 2011 Home-based	0.10	[-0.63,0.84]				N/A	N/A			
		Tonge 2006	-0.41	[-0.99,0.16]				-0.41	[-0.99,0.16]			

		Welterlin 2012	-0.23	[-1.11,0.65]				-0.23	[-1.11,0.65]			
