# Supplementary material for: A systematic review and meta-analysis of comprehensive interventions for pre-school children with autism spectrum disorder (ASD)
Source: PLoS One. 2017 Dec 6;12(12):e0186502. doi: 10.1371/journal.pone.0186502 (PMC5718481; doi:10.1371/journal.pone.0186502)
Supplement: S6 Table — (PDF) [file pone.0186502.s008.pdf]

S6 Table. Sensitivity analyses on the outcomes that were with significant baseline imbalances

		Random effects model						Fixed effects model			
		Analysis I			Analysis II			Analyses III		Analyses IV	
Outcome		p value	SMD (95%CI)	I^2(%)	p value	SMD (95%CI)	I^2(%)	p value	SMD (95%CI)	p value	SMD (95%CI)
Developmental quotient (sensitivity analysis)	○	0.07	0.31[-0.02,0.65]	26	0.04*	0.20[0.00,0.39]	0	0.04*	0.29[0.01,0.56]	0.04*	0.20[0.00,0.39]
Expressive language (sensitivity analysis)	○	0.18	0.13[-0.06,0.33]	0	0.02*	0.18[0.03,0.33]	0	0.18	0.13[-0.06,0.33]	0.02*	0.18[0.03,0.33]
Receptive language (sensitivity analysis)		0.30	0.12[-0.11,034]	24	0.25	0.10[-0.07,0.28]	10	0.33	0.09[-0.09,0.28]	0.27	0.09[-0.07,0.26]
Reciprocity of social intercation towards others (Sensitivity analysis)	◎	<0.01*	0.53[0.29,0.78]	18	<0.001***	0.53[0.29,0.78]	18	<0.001***	0.51[0.31,0.72]	<0.001***	0.51[0.31,0.72]
Adaptive behaviour (Sensitivity analysis)		0.69	-0.04[-0.23,0.15]	0	0.45	0.09[-0.15,0.34]	28	0.69	-0.04[-0.23,0.15]	0.78	0.03[-0.16,0.22]

p value indicates the value of the test of overall synthesis. SMD indicates standard mean difference of the overall synthesis effect. 95% CI indicates the 95% confidence interval of the standard mean difference of the overall synthesis. *, **, and *** indicate statistically significant effectiveness (p<0.05, p<0.01, and P<0.001, respectively) in the analysis. ○ indicates the outcome did not show significant effectiveness in the overall synthesis of Analysis I, but showed significant effectiveness in the sensitivity analysis. ◎ indicates the outcome showed significant effectiveness in both the overall synthesis of Analysis I and its sensitivity analyses; Analysis II, III, and IV. N/A indicates the analysis with overall synthesis could not be performed because only one study measured the outcome.
