# Supplementary material for: A systematic review and meta-analysis of comprehensive interventions for pre-school children with autism spectrum disorder (ASD)
Source: PLoS One. 2017 Dec 6;12(12):e0186502. doi: 10.1371/journal.pone.0186502 (PMC5718481; doi:10.1371/journal.pone.0186502)
Supplement: S7 Table — (PDF) [file pone.0186502.s009.pdf]

S7 Table. Comparison of the effect among Analysis I, II, III, and IV on each outcome in terms of statistical significanc

	Outcome	Analysis I	Analysis II	Analyses III	Analyses III
Primary outcome	Autism general symptoms	—	**	*	**
Secondary outcomes	Developmental quotient	*	*	*	*
	Developmental quotient (sensitivity analysis)	—	*	*	*
	Expressive language	—	*	—	*
	Expressive language (sensitivity analysis)	—	*	—	*
	Receptive language	—	—	—	—
	Receptive language (sensitivity analysis)	—	—	—	—
	Reciprocity of social interaction towards others	**	***	***	***
	Reciprocity of social interaction towards others (Sensitivity analysis)	**	***	***	***
	Adaptive behaviour	—	—	—	—
	Adaptive behaviour (Sensitivity analysis)	—	—	—	—
Other outcomes	Qualitative impairment in social interaction	—	—	—	—
	Qualitative impairment in communication	—	N/A	—	—
	Restricted repetitive and stereotyped patterns of behaviour, interests, and activities	—	—	—	—
	Initiating joint attention	—	**	**	**
	Responding to joint attention	—	—	***	***
	Imitation	—	*	—	**
	Symbolic play	N/A	—	N/A	—
	Functional play	N/A	**	N/A	**
	Parental synchrony	**	**	***	***
	Parenting stress	—	—	—	—

p value indicates the value of the test of overall synthesis. SMD indicates standard mean difference of the overall synthesis effect. 95% CI indicates the 95% confidence interval of the standard mean difference of the overall synthesis. *, **, and *** indicates statistically significant effectiveness (p<0.05, p<0.01, and P<0.001, respectively) in the analysis. — indicates the outcome did not show significant effectiveness in the overall synthesis. N/A means the analysis with overall synthesis could not be performed because only one study measured the outcome. “Sensitivity analysis” indicates the results of sensitivity analyses in which the studies with significant baseline imbalance between the intervention and the control groups with the outcome were excluded.
