# Supplementary material for: A systematic review and meta-analysis of comprehensive interventions for pre-school children with autism spectrum disorder (ASD)
Source: PLoS One. 2017 Dec 6;12(12):e0186502. doi: 10.1371/journal.pone.0186502 (PMC5718481; doi:10.1371/journal.pone.0186502)

S2 Appendix. Study protocol

A systematic review with meta-analysis of comprehensive interventions for pre-school children with autism spectrum disorder (ASD): study protocol

Yoshiyuki Tachibana M.D., Ph.D.[1, 2], Jonathan Green M.D.[1], Yeonhee Hwang Ph.D.[3], Richard Emsley Ph.D.[4]

[1] Department of Child and Adolescent Psychiatry, University of Manchester and Manchester Academic Health Sciences Centre, Manchester, UK

[2] Smart Aging International Research Centre, IDAC, Tohoku University, Sendai, Japan

[3] Special Support Education Research Centre, Tohoku Fukushi University, Sendai, Japan

[4] Health Methodology Research Group, Department of Biostatistics, University of Manchester and Manchester Academic Health Sciences Centre, Manchester, UK

Correspondence to

Yoshiyuki Tachibana M.D., Ph.D.

Department of Child and Adolescent Psychiatry, University of Manchester and Manchester Academic Health Science Centre

Room 4.321 (East), Jean McFarlane Building, University Place, University of Manchester and Manchester Academic Health Sciences Centre, Oxford Road, Manchester, M13 9PL, UK

Tel/Fax: +44 (0) 161 306 7941; E-mail: tatibana@idac.tohoku.ac.jp

Ver. 8.5

Date: 02/25/2012

ABSTRACT

Introduction: The aims of our study are to: i) conduct a systematic review of the intervention literature in preschool children with autism spectrum disorder (ASD), including types of interventions that are tested and the classification of outcome measures used; ii) to undertake a meta-analysis of the studies, allowing for the first time the comparison of different approaches to intervention using comparative outcomes. There are a number of alternative modalities of intervention for preschool children with ASD in use with different theoretical background and orientation, each of which tend to use different trial designs and outcome measures. There is at this time an urgent need for comprehensive systematic review and meta-analyses of intervention studies for preschool children with ASD, covering studies of adequate quality across different intervention types and measurement methods, with a view to identifying the best current evidence for preschool interventions in the disorder.

Methods and analysis: We will perform a systematic review of RCTs for preschool children with ASD aged 0 to 6, along with a meta-analysis of qualifying studies across intervention modality. We will classify the interventions for preschool children with ASD under three models; behaviour, multi-modal developmental, and communication-focused. Firstly, we will perform a systematic review. Then, we will conduct a meta-analysis by comparing the three models with various outcomes using an inverse variance method in a random effect model. We will synthesise each outcome of the studies for the three models using standardised mean differences.

Dissemination and ethics: This study will identify each intervention's strengths and weaknesses. This study may also suggest what kinds of elements future intervention programmes for children with ASD should have. We strongly believe those findings will be able to translated into the clinical practices and patients and their family benefits.

Trial registration:

http://www.crd.york.ac.uk/prospero/register_new_review.asp?RecordID=1349&UserID=230 (Registration No. CRD42011001349)

INTRODUCTION

Recent epidemiological studies estimate a prevalence of 1:100 for autism spectrum disorder (ASD) [1], an increase over reported rates in the past [2]. There has been increasing interest in developing effective interventions for young children with ASD, since the evidence suggests that early intervention programmes are indeed beneficial for children with ASD, often improving developmental functioning and decreasing maladaptive behaviours and symptom severity [3], and also can improve outcomes in later years for many individuals [4].

An increasing volume of published trials of psychosocial intervention programmes for preschool children with ASD have been seen in recent years. These programmes tend to fall into three models; i) those based on behaviour change which use applied behavioural analysis (ABA) (e.g. [5]); ii) those focused on therapies targeted at improving the social communication impairment, the core symptom of autism (e.g. [6]); iii) multimodal interventions targeted across areas of autistic children's development (e.g. [7]). In addition, an increasing number of these studies have followed CONSORT guidelines [8], and some meta-analyses and systematic reviews about intervention programmes for preschool children with ASD have been published; e.g. [9-11]. These meta-analyses and systematic reviews focused exclusively on one or the others of these groups of intervention styles; there has been no systematic review or meta-analysis of studies comparing results from different types of intervention approach from the viewpoint of the three models. For clinicians and commissioners this poses a difficulty in making general choices in a field containing often strong and partisan claims of effect from different traditions of intervention. Related to this, there has been great variation in endpoint measures used in these reported studies, making comparison of effects between studies difficult. Specifically, there has been variation in whether endpoints have been framed in terms of specific autism symptom outcomes, non autism-specific

outcomes that are not specific to autism (such as for instance IQ), or ‘intermediate’ endpoints relating to aspects of development that may have some relationship to later autism symptoms – examples would be changes in joint attention or parent-child interaction. These latter two kinds of outcome are often reported, without necessarily strong justification, as if they were the equivalent of change in autism symptoms (i.e. as ‘surrogate’ endpoints); and this can cause real confusion. We think that these considerations indicate the need for a more comprehensive review of intervention studies for preschool children with ASD, covering studies of adequate quality across different intervention types and measurement methods, with a view to identifying the best current evidence for preschool interventions in the disorder. In this study, we will investigate it by comparing three major types of interventions with various outcomes.

We will undertake a systematic review and a meta-analysis of RCTs for preschool children with ASD. Recently, many RCTs for children with ASD have emerged sufficient enough to perform meta-analyses. RCT methodology has been identified as the gold standard in efficacy research [12]. In addition, meta-analyses of RCTs is at the top of the evidence based medicine hierarchy [13]. Thus, the findings of this study will provide strong evidence about interventions for children with ASD. Howlin et al. are asserting that there are three main strands of early interventions for children with ASD): programmes with a particular emphasis on the use of behavioural principle to improve learning and behaviour; those that have a specific focus on communication; and those in which developmental/educational strategies have been employed [14]. In this study, we named those strands as behavioural, communication-focused, and multimodal developmental interventions, respectively. Understanding the mechanisms that underlie this attenuation of treatment effects and how these can be overcome is one current challenge [15]. This study may reveal each type of the intervention’s strong and weak points to various kinds of treatment factors respectively. Its findings will guide us to

develop new types of interventions to overcome the attenuation of treatment effects in the core symptoms of autism. It will contribute to the appropriate choices of the interventions for children with ASD for their families, clinicians, and the policymakers.

The objective of our study is to: i) conduct a systematic review of all the preschool intervention literature in ASD, including the type of intervention that is being tested and classification of outcome measures used; ii) to undertake a meta-analysis of methodologically adequate studies using the Cochrane tool, which will allow for the first time comparison of different approaches to intervention on comparative outcome measures.

METHODS

Types of studies

We will include randomised controlled trials and subject these to a rating on the Cochrane Collaboration tool for assessing risk of bias.

Types of participants

Participants comprise preschool children aged 0 to 6 with a diagnosis of ASD as below.

Diagnostic and statistical Manual of Mental Disorders-Ⅳ-Text Revision (DSM-Ⅳ-TR) [16]

- Autistic disorder
- Asperger disorder
- Pervasive developmental disorder not otherwise specified (PDD-NOS)

International Classification of Diseases-10 (ICD-10) [17]

- Childhood autism
- Asperger syndrome, atypical autism

- Other pervasive developmental disorders
- Pervasive developmental disorders, unspecified.

Types of interventions

We classify interventions for preschool children with ASD in three groups; i) behavioural interventions – based essentially on learning theory and on applied behaviour analysis; ii) communication-focused interventions, targeting social communication impairment, as the core symptom of autism; iii) multimodal developmental interventions targeting a range of aspects of children's development.

Types of outcome measures

A feature of this review is that we will systematically classify the various outcome measures used within recent intervention trials into the following categories:

Primary outcomes

Autism behavioural symptoms: qualitative impairment in social interaction; qualitative impairment in communication; restricted repetitive and stereotyped patterns of behaviour, interests, and activities. These are the triad of diagnostic criteria for autism in DSM-Ⅳ-TR and the definitional symptoms of the disorder and key indicators of psychopathology (e.g. the autism Diagnostic Observation Schedule-Generic [18] will be used for these outcomes.).

Secondary outcomes

Non-specific developmental outcomes. These are not directly related by definition to autism diagnosis but are used in some studies as substitute outcomes – examples are adaptive behaviour (e.g. the Vineland Adaptive Behaviour Scale [19] will be used for this

outcome), and IQ and cognitive development (e.g. the Wechsler Preschool and Primary Scale of Intelligence third edition [20] will be used for these outcomes.).

Intermediate outcomes relevant to the known development of autism – which might be candidates for surrogate endpoints. These outcomes are often defined as the proximal targets of intervention approaches from a developmental perspective. Examples (along with appropriate measures) are: measures of joint attention (the Early Social Communication Scales [21]), imitation ability (the Imitation Battery [22]), symbolic play (the Communication and Symbolic Behaviour Scales Developmental Profile [23]), parent-child interaction (the Dyadic Communication Measure for Autism [24]), receptive language (the MacArthur-Bates Communicative Development Inventory (MCDI [25])), expressive language (MCDI [25]).

Electronic searches

We will do a systematic review of the published work according to the PRISMA statement [26] (Figure 1). Relevant studies will be identified by searching the following data sources: PsycINFO (from 1956 to January, 2011), Medline via Ovid (from 1950 to January, 2011), ERIC (from 1950 to January, 2011) and the Cochrane database.

We will use the following search terms to search all trials registers and databases: “autism” , “autism spectrum disorder”, “ASD”, “high function autism”, “high function ASD”, “Asperger syndrome”, “pervasive developmental disorder”, “PDDNOS”, “intervention”, “treatment”, “therapy”, “communication”, “interpersonal”, “speech”, “interaction”, “synchrony”, “relationship”, “language”, “social”, “development”, “behavior”, “intensive behavioral intervention”, “trial”, and “outcome”. Their search will be limited by age group from 0 to 6 years old and “randomized controlled trial.” This search strategy has been peer-reviewed by a librarian of University of Manchester.

Validity assessment

Two of the authors, Y.T., Y.H. will independently review the abstracts of the potentially relevant studies. This will be followed by a consensus discussion with J.G. The quality of the RCTs will be coded independently by Y.T. and Y.H. and disagreements will be resolved by consensus discussions.

Searching other resources

Reference lists from identified trials and review articles will be manually scanned to identify any other relevant studies. The clinicalTials.gov and the Cochrane Library website will be also searched for randomised trials that were registered as completed but not yet published.

Data collection and analysis

Selection of studies

Inclusion:

1. Participants comprise preschool children with a diagnosis of ASD or pervasive developmental disorder (PDD).

2. Randomised controlled trials

3. Interventions delivered to the parents/guardians and/or directly to the child, by special educators, teachers, speech pathologists, psychologists, or other allied health professional students will be included.

4. Studies carried out while the children were at a preschool age between 0 and 6 years.

Exclusion:

1. The study was not primary research on preschool children with ASD.

2. The study did not assess a cognitive/behavioural intervention for preschool children with ASD.

3. The study design was not a randomised controlled trial.

4. Alternative or complementary medicine was used as the main intervention of the study.

5. The intervention was a pharmacological one.

6. The intervention was not classified into behavioural, multimodal developmental or communication-focused model.

7. The control group received a specific early intervention programme for children with autism which was not a usual treatment provided by their local services.

8. The study was judged to be in high risk of bias by the Cochrane Collaboration tool for assessing risk of bias.

All citations sourced from the search strategy will be transferred to EndNote, a reference management database software. Initial screening of titles and abstracts by an experienced research fellow (YT) will eliminate all those citations obviously irrelevant to the topic, for example, prevalence studies, studies not relating to autism spectrum disorders, single case studies. Thereafter, two review authors (YT and YH) will assess and select studies for inclusion from the group of superficially relevant studies. In the event of a disagreement, resolution will be reached in discussion with the third author (JG), if necessary following inspection of the full paper.

Data extraction and management

YT and YH will independently extract data from selected trials using a specially designed data extraction form. Extracted data will consist of methods (dose and frequency of intervention); diagnostic description of participants, and type of

intervention, including target, intensity, duration and method of application (parent-mediated, therapist, school-based etc.). Data will be extracted independently by two review authors (YT and YH) and disagreements will be resolved by negotiation with a third author (JG).

Assessment of risk of bias in the studies

Risk of bias will be assessed by two independent review authors (YT and YH) and disagreements will be resolved by negotiation with a third review author (JG). We will use the Cochrane Collaboration tool for assessing risk of bias in these areas [27]. The assessed risk of bias in studies will include in the following domains: sequence generation; allocation concealment; blinding; incomplete outcome data; selective outcome reporting; other sources of bias. The process will involve recording the appropriate information for each study (for example describing the method used to conceal allocation in detail) and evaluating whether there is risk of bias in that area (for example, was allocation adequately concealed). We will allocate studies to the three categories according to our judgment of each area or potential risk of bias: A. Low risk of bias; B. Moderate (or unclear) risk of bias; C. High risk of bias. Whether the studies should be included for the analyses or not will be judged individually based on the results of the risk of bias assessments.

Measures of treatment effect

Continuous data

Continuous data will be analysed on the basis that the means and standard deviations are available and that there is no clear evidence of skew in the distribution.

Dealing with missing data

Missing data will be assessed for each individual study. Where a loss of significant quantities of participant data is reported such that the review authors agree that the conclusions of the study are compromised, trial authors will be contacted. If no reply is forthcoming or full data are not made available, these studies will not be included in the final analysis. For included studies reporting drop-out, we will report the number of participants included in the final analysis as a proportion of those participants who began the intervention. Reasons for missing data will be reported. The extent to which the results of the review could be altered by the missing data will be assessed and discussed. If summary data are missing, trial authors will be contacted. If no reply is forthcoming or the required summaries are not made available, the authors will include the study in the review and assess and discuss the extent to which its absence from meta-analysis affects the review results.

Assessment of heterogeneity

Consistency of results will be assessed visually and by chi-square tests [28]. In addition, since chi-square can have low power when only few studies or studies of a small sample size are available [29], we will use the I^2 statistic to calculate the degree to which heterogeneity is having an impact on the analysis [27].

Assessment of reporting biases

If sufficient studies are found, funnel plots will be drawn to investigate any relationship between effect size and sample size. Such a relationship could be due to publication or related biases, or due to systematic differences between small and large studies. If a

relationship is identified, clinical diversity of the studies will be further examined as a possible explanation. Every attempt will be made to obtain unpublished data and data from conference proceedings.

Data synthesis

Data synthesis will be performed using Review Manager version 5.1 (Cochrane Collaboration software). We will assess continuous and binary data. Assuming that two or more studies that are suitable for inclusion are found, and that the studies are considered to be homogenous, a meta-analysis will be performed on the results. The categories of outcome measure mentioned above differ conceptually in important ways, and have been used in a systematic different way across trials of the different intervention types identified above. Our review aims to make comparison across these different types of intervention study, thus we will standardise and synthesise the various categories of outcome measure using an inverse variance method in a random effect model [27]. We will compare the types of intervention model effectiveness for each outcome using a standardised mean difference.

Subgroup analysis and investigation of heterogeneity

We will undertake subgroup analyses and meta-regression. These will be pooled to calculate a final effect size. While these analyses may enable us to hypothesise as to possible causes of differences between studies' findings, some heterogeneity is likely to remain, and any statistical analysis will be accompanied by a narrative synthesis.

Subgroup analysis will be undertaken if clinically different interventions are identified, or there are clinically relevant differences between participant groups. Anticipated clinically relevant differences are:

1. intervention delivery type (e.g. therapist, parent-mediated, school-based) and length
2. intervention target skill (e.g. Theory of Mind as a whole, joint attention, emotion recognition, false belief understanding)
3. participant age (e.g. preschool, young children), IQ (low versus normal or high), specific diagnosis and verbal ability.

Relevant subgroup analyses will also include:

·Severity of autism at baseline

·Social economic status and other demographic variables

·Age of child

·Type of intervention (our 3 groups as above)

·Parent-mediated (directing parents to train their children, not training the children directly) vs. child-mediated (training the children directly) intervention delivery

·Cognitive ability at baseline

Sensitivity analysis

Sensitivity analysis will be conducted to assess the impact of study quality on the results of the meta-analyses. For example, we will test to see if studies with high rates of loss to follow up or inadequate blinding are more likely to show positive outcomes and also to assess the impact of imputing missing data.

DISCUSSION

Meta-analysis of RCTs across types of intervention for preschool children with ASD is an important step in providing a reliable basis for implementation decisions. Since previous analyses have been essentially restricted to specific intervention types, and often with different outcome criteria, a study across three representative models:

behavioural, multimodal developmental or communication-focused models will guide future clinical practice and research trials for children with ASD. This study will provide information about which kind of intervention has strong points and weak points, and what are those strong points and weak points are. This study may also suggest what kinds of elements future intervention programmes for children with ASD should have. We strongly believe those findings will be able to translated into the clinical practices and patients and their family benefits.

Anticipated challenges in synthesise the literature exist. The measures used for outcome are varied between studies and the standardised data will be heterogeneous. We do not assume that each study is estimating exactly the same quantity. Thus, we will use random effect models for the analyses [27]. In addition, the durations of the interventions will be different among the studies included in this study. We will synthesise the data regardless of the durations of the interventions, and will discuss the diversity of the durations in our paper.

Authors' contribution YT and JG contributed to draft the protocol and develop a search strategy. YT also drafted this manuscript. RE contributed to provide statistical advice for the design and the analysis. All authors read and approved the final manuscript.

Acknowledgement We thank Claire Hodkinson for the peer-reviewing of this study's search strategy.

Competing interests None.

Funding This study was supported by JSPS titled 'Institutional Program for Young Researcher Overseas Visits'.

References

1. Baird G, Simonoff E, Pickles A, Chandler S, Loucas T, Meldrum D, et al. Prevalence of disorders of the autism spectrum in a population cohort of children in South Thames: the Special Needs and Autism Project (SNAP). *The Lancet* 2006;368(9531):210-15.
2. Newschaffer CJ, Croen LA, Daniels J, Giarelli E, Grether JK, Levy SE, et al. The epidemiology of autism spectrum disorders*. *Annu. Rev. Public Health* 2007;28:235-58.
3. Rogers SJ, Vismara LA. Evidence-based comprehensive treatments for early autism. *Journal of clinical child and adolescent psychology: the official journal for the Society of Clinical Child and Adolescent Psychology, American Psychological Association, Division 53* 2008;37(1):8.
4. United States Government Accountability Office. *Special education: children with autism; report to the Chairman and Ranking Minority Member, Subcommittee on Human Rights and Wellness, Committee on Government Reform, House of Representatives*. Darby, PA: DIANE Publishing, 2005.
5. Smith T, Groen AD, Wynn JW. Randomized trial of intensive early intervention for children with pervasive developmental disorder. *American Journal on Mental Retardation* 2000;105(4):269-85.
6. Green J, Charman T, McConachie H, Aldred C, Slonims V, Howlin P, et al. Parent-mediated communication-focused treatment in children with autism (PACT): a randomised controlled trial. *The Lancet* 2010;375(9732):2152-60.
7. Dawson G, Rogers S, Munson J, Smith M, Winter J, Greenson J, et al. Randomized, controlled trial of an intervention for toddlers with autism: the Early Start Denver Model. *Pediatrics* 2010;125(1):e17.
8. Campbell MK, Elbourne DR, Altman DG. CONSORT statement: extension to cluster

randomised trials. *BMJ* 2004;328(7441):702.

9. Eldevik S, Hastings RP, Hughes JC, Jahr E, Eikeseth S, Cross S. Meta-analysis of early intensive behavioral intervention for children with Autism. *Journal of Clinical Child and Adolescent Psychology* 2009;38(3):12.

10. Spreckley M, Boyd R. Efficacy of applied behavioral intervention in preschool children with autism for improving cognitive, language, and adaptive behavior: a systematic review and meta-analysis. *The Journal of pediatrics* 2009;154(3):338-44.

11. Makrygianni MK, Reed P. A meta-analytic review of the effectiveness of behavioural early intervention programs for children with autistic spectrum disorders. *Research in Autism Spectrum Disorders* 2010;4(4):577-93.

12. Smith T, Scahill L, Dawson G, Guthrie D, Lord C, Odom S, et al. Designing research studies on psychosocial interventions in autism. *Journal of autism and developmental Disorders* 2007;37(2):354-66.

13. Summerskill WSM. Hierarchy of evidence. *Key Topics in Evidence Based Medicine. Oxford: Bios Scientific Publishers* 2001.

14. Howlin P, Magiati I, Charman T. Systematic review of early intensive behavioral interventions for children with autism. *Journal Information* 2009;114(1).

15. Charman T. Commentary: Glass half full or half empty? Testing social communication interventions for young children with autism-reflections on Landa, Holman, O'Neill, and Stuart (2011). *Journal of Child Psychology and Psychiatry* 2011;52(1):22-23.

16. American Psychiatric Association. *Diagnostic and statistical manual of mental disorders: DSM-IV-TR*. Arlington, VA: American Psychiatric Publishing, Inc., 2000.

17. World Health Organization. *The ICD-10 classification of mental and behavioural*

disorders: diagnostic criteria for research. Geneva: World Health Organization, 1993.

18. Lord C, Risi S, Lambrecht L, Cook EH, Leventhal BL, DiLavore PC, et al. The Autism Diagnostic Observation Schedule—Generic: A standard measure of social and communication deficits associated with the spectrum of autism. *Journal of autism and developmental Disorders* 2000;30(3):205-23.
19. Sparrow SS, Cicchetti DV. *The Vineland Adaptive Behavior Scales*. Boston, MA: Allyn & Bacon, 1989.
20. Wechsler D. *Wechsler Preschool and Primary Scale of intelligence third edition*: San Antonio, TX: Harcourt Assessment, Inc, 2002.
21. Mundy P, Delgado C, Block J, Venezia M, Hogan A, Seibert J. Early Social Communikation Scales (ESCS). *Coral Gables, FL: University of Miami* 2003.
22. Rogers SJ, Hepburn SL, Stackhouse T, Wehner E. Imitation performance in toddlers with autism and those with other developmental disorders. *Journal of Child Psychology and Psychiatry* 2003;44(5):763-81.
23. Wetherby AM, Prizant BM. *Communication and Symbolic Behavior Scales: Developmental Profile*. Baltimore, MD: Paul H Brookes Publishing, 2002.
24. Aldred C, Green J, Adams C. A new social communication intervention for children with autism: pilot randomised controlled treatment study suggesting effectiveness. *Journal of Child Psychology and Psychiatry* 2004;45(8):1420-30.
25. Fenson L. *MacArthur-Bates Communicative Development Inventories: user's guide and technical manual*. Baltimore, MD: Paul H. Brookes Pub. Co., 2007.
26. Moher D, Liberati A, Tetzlaff J, Altman DG. Preferred reporting items for systematic reviews and meta-analyses: the PRISMA statement. *PLoS Medicine* 2009;6(7):e1000097.
27. Higgins J, Green S. *Cochrane Handbook for Systematic Reviews of Interventions*

Version 5.1. 0 [updated March 2011]. Oxford: The Cochrane Collaboration, 2011.

28. Egger M, Smith GD, Altman DG. Statistical Methods for Examining Heterogeneity and Combining Results from Several Studies in Meta-Analysis. *Systematic Reviews in Health Care: Meta-Analysis in Context, Second Edition*. Oxford: Blackwell Publishing Ltd., 2001:285-312.
29. Higgins J, Thompson SG, Deeks JJ, Altman DG. Measuring inconsistency in meta-analyses. *BMJ* 2003;327(7414):557.

Figure 1. Flow diagram of this study

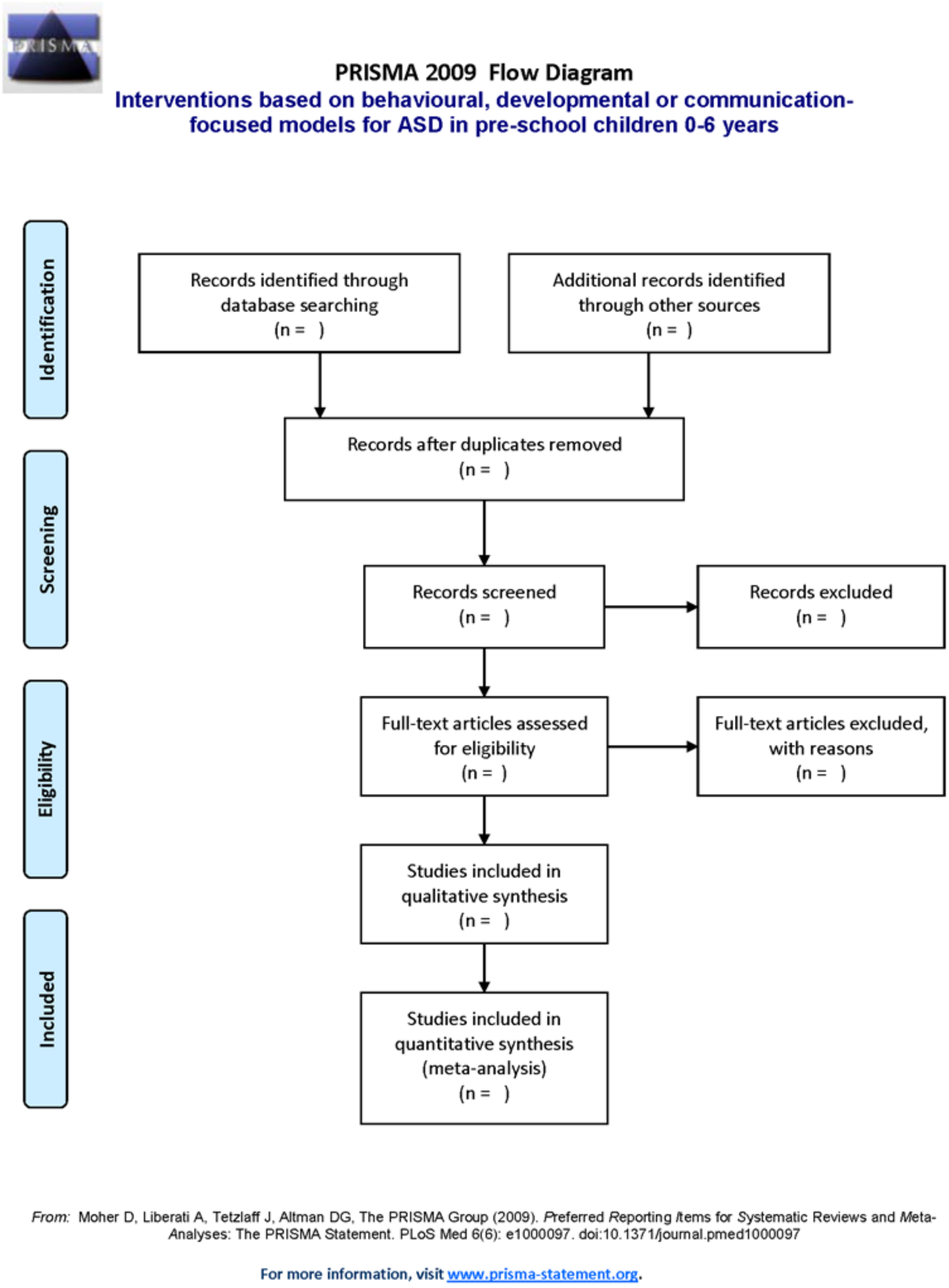

Supplement: S2 Appendix — (PDF) [file pone.0186502.s012.pdf]
